# A comparison of temporal trends in United States autism prevalence to trends in suspected environmental factors

**DOI:** 10.1186/1476-069X-13-73

**Published:** 2014-09-05

**Authors:** Cynthia D Nevison

**Affiliations:** Institute for Arctic and Alpine Research, University of Colorado, Boulder, Boulder, CO 80309-0450 USA

**Keywords:** Autism, Temporal trends, Air pollution, Mercury, Vaccines, Organophosphates, PBDEs

## Abstract

**Background:**

The prevalence of diagnosed autism has increased rapidly over the last several decades among U.S. children. Environmental factors are thought to be driving this increase and a list of the top ten suspected environmental toxins was published recently.

**Methods:**

Temporal trends in autism for birth years 1970–2005 were derived from a combination of data from the California Department of Developmental Services (CDDS) and the United States Individuals with Disabilities Education Act (IDEA). Temporal trends in suspected toxins were derived from data compiled during an extensive literature survey. Toxin and autism trends were compared by visual inspection and computed correlation coefficients. Using IDEA data, autism prevalence vs. birth year trends were calculated independently from snapshots of data from the most recent annual report, and by tracking prevalence at a constant age over many years of reports. The ratio of the snapshot:tracking trend slopes was used to estimate the "real" fraction of the increase in autism.

**Results:**

The CDDS and IDEA data sets are qualitatively consistent in suggesting a strong increase in autism prevalence over recent decades. The quantitative comparison of IDEA snapshot and constant-age tracking trend slopes suggests that ~75-80% of the tracked increase in autism since 1988 is due to an actual increase in the disorder rather than to changing diagnostic criteria. Most of the suspected environmental toxins examined have flat or decreasing temporal trends that correlate poorly to the rise in autism. Some, including lead, organochlorine pesticides and vehicular emissions, have strongly decreasing trends. Among the suspected toxins surveyed, polybrominated diphenyl ethers, aluminum adjuvants, and the herbicide glyphosate have increasing trends that correlate positively to the rise in autism.

**Conclusions:**

Diagnosed autism prevalence has risen dramatically in the U.S over the last several decades and continued to trend upward as of birth year 2005. The increase is mainly real and has occurred mostly since the late 1980s. In contrast, children’s exposure to most of the top ten toxic compounds has remained flat or decreased over this same time frame. Environmental factors with increasing temporal trends can help suggest hypotheses for drivers of autism that merit further investigation.

**Electronic supplementary material:**

The online version of this article (doi:10.1186/1476-069X-13-73) contains supplementary material, which is available to authorized users.

## Introduction

Autism was first described in the 1930s as a novel clinical disorder characterized by impairment in social interaction and communication, and restricted and stereotyped patterns of interests and behaviors [1]. Today, one in every 68 children in the United States, including 1 in every 42 boys, is diagnosed with an autism spectrum disorder (ASD) [2]. In comparison, the prevalence of diagnosed autism was about 1 in 2,500 in the early 1970s [3]. While these numbers suggest a dramatic increase in ASD prevalence over the past few decades, there is ongoing debate and uncertainty over how much of the rise in autism is due to a true increase in the condition as opposed to better and expanding diagnosis [4, 5].

At the biological level, autism is characterized by a number of increasingly well-documented biochemical imbalances. These include redox imbalance, oxidative stress and associated mitochondrial dysfunction and deficiency in glutathione [6–10]. Imbalances in gut microflora are common and the likelihood of suffering from a gastrointestinal disorder is greatly enhanced relative to neurotypical control populations [11, 12]. Autistic individuals also display proinflammatory cytokine imbalances and may suffer from overactive or dysfunctional immune systems, with chronic neuroinflammation, including neuroglial activation in the brain, and the presence of autoantibodies to brain proteins [13–15]. Two recent reviews have converged on immune system dysregulation as the core biological feature of autism [16, 17], although oxidative stress and immune function are interrelated, with the one influencing the other in a two-way interaction [18].

Some have argued that autism is primarily genetically based and has always been present in the human population at current levels [4]. From this genetic perspective, temporal trends in autism and toxins are largely irrelevant. Rather, the rise in autism diagnosis reflects the successful efforts of the public health community to better identify children who went undiagnosed in previous generations, to promote inclusion and acceptance of those children and to provide them with early intervention services. Others have argued that autism is caused primarily by environmental triggers acting on a genetically susceptible subset of children and that epigenetics play an important role in mediating how environmental toxins affect gene expression [19]. A recent comprehensive study of ASD concordance rates among dizygotic and monozygotic twins supports the dominant influence of environmental factors, while also confirming the importance of genetic susceptibility [20]. From this alternative perspective, since genes alone do not mutate rapidly, temporal trends in environmental toxins are relevant and can provide valuable clues into the causes of autism [3].

Recently, a list of the top ten environmental compounds suspected of causing autism and learning disabilities was published [21]. The list includes lead, methylmercury, polychorinated biphenyls, organophosphate pesticides, organochlorine pesticides, endocrine disruptors, automotive exhaust, polycyclic aromatic hydrocarbons, polybrominated diphenyl ethers, and perfluorinated compounds. The list was based largely on epidemiological studies showing an increased risk of autism or related pervasive developmental delay (PDD) with increased exposure to the compounds [22–24]. While the top ten compounds comprise only a small subset of the > 80,000 synthetic chemicals developed over the past 50 years, many of which have never been assessed for potential toxicity, they were intended as a short list for which concentrated study has a high potential to generate actionable results in the near future [21].

Here, the temporal trends in the top ten environmental compounds list are systematically reviewed with the goal of identifying those that are most and least consistent with the temporal trends in autism. The analysis is focused on autism and does not address other, in many cases well-established harmful effects of the compounds. The investigation is guided primarily by the top ten list, but is expanded to include general air pollution indices and a broad range of mercury exposures, including fish, high fructose corn syrup, atmospheric mercury, and vaccine-administered thimerosal. In addition, trends in other vaccine-related indices, autoimmune disorders, and lifestyle factors such as obesity are examined. Some implicit assumptions are 1) that environmental exposures around the time of birth (± ~1.5 years) are the most important, since autism by definition is either present from birth or develops within the first few years of life [1, 4, 21], and 2) that the rise in autism is driven by one or more environmental influences whose collective temporal trend resembles the trend in autism [3].

Before embarking on the trend analysis of the suspected environmental compounds, the temporal trend in autism itself is examined. The trend is defined and visualized by plotting autism prevalence vs. birth year, which permits direct comparison with trends in environmental factors, given assumption 1 above. A new, empirical approach is applied to test the hypothesis that autism is a constant-prevalence condition that simply has been underdiagnosed in the past. This approach is based on the use of "constant-age tracking" and "age-resolved snapshots" as two independent methods for estimating the temporal trend in autism. The "constant-age tracking" method is the most common and straightforward way to quantify the trend. It involves tracking children of a specific age over multiple, successive years of reports, e.g., 8 year-olds in the biannual Autism and Developmental Disabilities Monitoring (ADDM) Network reports [2]. The constant-age tracking method suggests a strong increase in the prevalence of diagnosed autism in the U.S. over the last few decades, both in the ADDM network and in data from the Individuals with Disabilities Education Act (IDEA), which are used in the current study.

IDEA data have the advantage that each individual year’s report gives separate autism counts for each age between 5 and 17 years old, effectively providing a snapshot, resolved by age, for that year. Thus, with simple algebra, a prevalence vs. birth year curve can be constructed from any individual IDEA report, providing an independent, alternative approach to constant-age tracking for estimating the temporal trend in autism. This alternative approach is referred to here as the "age-resolved snapshot" method. A key point is that the temporal trend derived from an age-resolved snapshot should be more or less immune to the biasing influences of better and expanding diagnosis, since these influences in principle will affect all ages in the snapshot equally as one moves from the IDEA reports of the early 1990s to the most recent one in 2010. In other words, if the hypothesis that autism is a constant-prevalence condition is correct, a snapshot-based prevalence vs. birth year plot should be essentially a flat line with equal prevalence at all ages – albeit a line whose absolute value rises with each new report as diagnosis continues to improve.

A caveat to the above statement is that some of the older children in the snapshot may remain undiagnosed despite the increased awareness of autism in recent years. This is possible despite the fact that all school children with an autism diagnosis are entitled to valuable, publicly-funded IDEA services [4], and that an older child is eligible for reevaluation for autism even if he was overlooked or misdiagnosed in earlier years. However, if the constant-prevalence hypothesis is correct, even if some of the older children remain undiagnosed and the snapshot-based prevalence vs. birth year plot is not a perfect flat line, it still should have a substantially flatter slope than a constant-age-tracking-based prevalence vs. birth year plot covering the same time interval. This hypothesis is tested empirically below using a self-consistent dataset and the results and their implications are discussed.

## Methods

### Autism prevalence

#### IDEA data: constant-age tracking vs. age-resolved snapshots

Autism counts were obtained from the Individuals with Disabilities Education Act (IDEA) database (http://www.ideadata.org) for each of the 50 U.S. states plus the District of Columbia. Autism counts for children age 6 through 17 are available in age-resolved annual reports for 1991 through 2010, while autism counts for 5 year-olds are available starting in the 2000 report. Prevalence was calculated by dividing the IDEA autism counts by total statewide public school populations from the National Center for Education Statistics (NCES) (http://nces.ed.gov/ccd/bat/). The NCES data are resolved by grade from kindergarten (age 5) to 12^th^ grade (age 17) and available in annual reports from 1991 to 2010.

The temporal trend in autism for each state was derived by plotting prevalence vs. birth year using both the "constant-age tracking" and "age-resolved snapshot" methods. Birth year was calculated according to Equation (1).
1

In the tracking method, *Age* was held constant while *Report Year* was varied. In the snapshot method, *Report Year* was held constant while *Age* was varied from 5 to 17 years. Constant-age tracking trends were calculated using the twenty available years of IDEA reports (1991–2010) for each of the following ages: 8, 9, 10, and 11 years old. In addition, the trend among 5 year-olds was tracked over the eleven available years of reports (2000–2010), which enabled the examination of trends through as late as birth year 2005. The trend in autism also was calculated independently from age-resolved snapshots using IDEA reports from the following individual years: 2002, 2005 and 2010.

**Figure 1:**
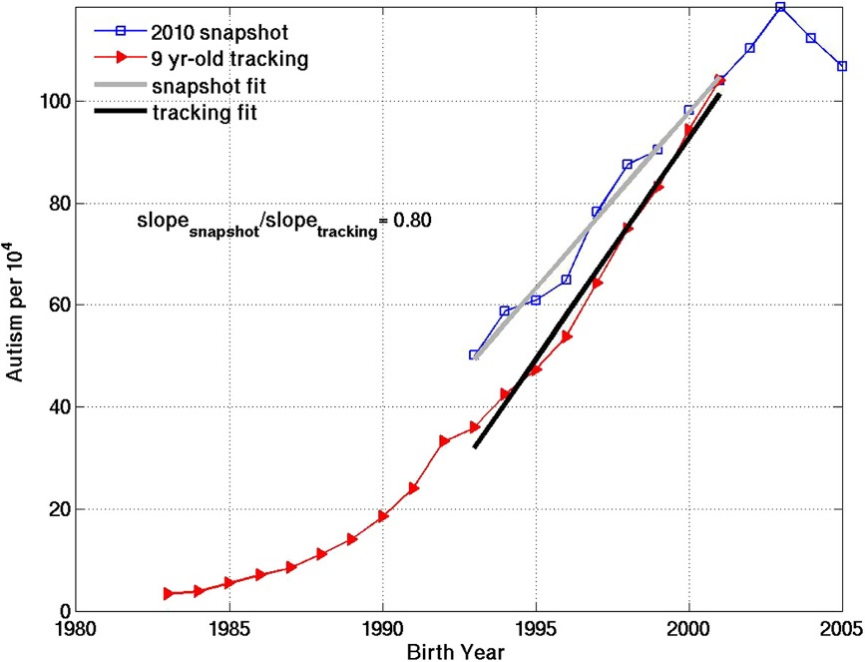
**Autism prevalence vs. birth year for California IDEA data, derived using two independent methods: 1) Constant-age tracking of 9 year-olds over 20 years of annual reports from 1991–2010 (red) and 2) Age-resolved snapshot from the most recent report in 2010 (blue).** The slope of each curve over the same birth year interval, 1993–2001, is estimated with a least squares linear fit. The snapshot fit (grey) spans ages 9–17 in the 2010 report. The constant-age tracking fit spans report years 2002–2010. The snapshot:tracking slope ratio over the 1993–2001 birth year interval is 0.80.

The slopes of the temporal trends were quantified over 7–10 year intervals for each of the 50 states + D.C. by least squares linear regression. The errors in the trend slopes were taken from the covariance matrix of the regression. The linear regression approach assumes that the autism prevalence vs. birth year relationship can be represented more or less as a linear increase over short intervals of data. The assumption of linearity is generally not appropriate for the younger end of the age-resolved snapshots, when the prevalence vs. birth year curves tend to flatten out and decline due to under-ascertainment in younger children (Figure 1 and Additional file 1: Figure S1). To avoid bias due to under-ascertainment, the youngest ages: 5, 6 and 7, were discarded in calculating the snapshot trend slopes. To account for uncertainty in the age at which under-ascertainment ceases to bias the snapshot trend slope, a range of start ages from 8 to 11 years old was considered. Using Equation (1), with the age 8 to 11 range in start age and the end age held constant at 17, the 2005 snapshot corresponds to the birth year interval 1988 through 1994–1997, while the 2010 snapshot corresponds to the birth year interval 1993 through 1999–2002. Constant-age-tracking trend slopes were calculated over these same birth year intervals for ages 8, 9, 10 and 11.

The ratio of the snapshot slope to the tracking slope was calculated over exactly overlapping birth year intervals, such that the tracked age was the same as the start age of the snapshot slope. For example, 9 year-old tracking slopes were compared to snapshot slopes beginning at age 9, while 10 year-old tracking slopes were compared to snapshot slopes beginning at age 10 (illustrated in Figure 1 and Additional file 1: Figure S2a for California for the 2010 snapshot). The snapshot:tracking slope ratio calculations were motivated by the following considerations: If the snapshot-based trend is a flat line with zero slope (i.e., if autism is truly a constant-prevalence condition, fully diagnosed at all ages in the snapshot), the slope ratio will be 0. Conversely, if the snapshot and tracking trends agree perfectly in showing a strong increase over time, the slope ratio will be 1. If the truth lies somewhere in between, the ratio of the snapshot slope to the tracking slope can provide a quantitative measure of the fraction of the constant-age tracking-based increase in autism that is "real" rather than attributable to better or expanded diagnosis.

#### CDDS data

In addition to IDEA data, autism prevalence data for birth years 1970–1997 were obtained from the California Department of Developmental Services (CDDS) [3]. The CDDS data are effectively a 2002 age-resolved snapshot of individuals 5 years of age or older receiving services for autism [25]. The CDDS data are one of the most reliable, long-term U.S. autism records available, although they are limited to California. CDDS data include only verified cases of full-syndrome autistic disorder (AD), the most severe and unambiguous ASD.

Additional CDDS autism prevalence data tracking 5 year-olds from 1995–2006, corresponding to birth years 1990–2001, were obtained [26]. The CDDS tracking data set overlaps with California IDEA data tracking 5 year-olds for birth years 1995–2001, providing a means for assessing whether the IDEA definition of autism has expanded from the CDDS definition to include milder ASDs. Since children in private schools are included in IDEA autism counts but not in NCES total public school population data, the IDEA/NCES ratio will tend to overestimate autism prevalence, by underestimating the denominator. To correct for this effect in all figures in which CDDS and California IDEA data are combined, the NCES total populations for California were revised upward by 14% based on available U.S. census data.

#### California IDEA and CDDS composite trend

**Figure 2:**
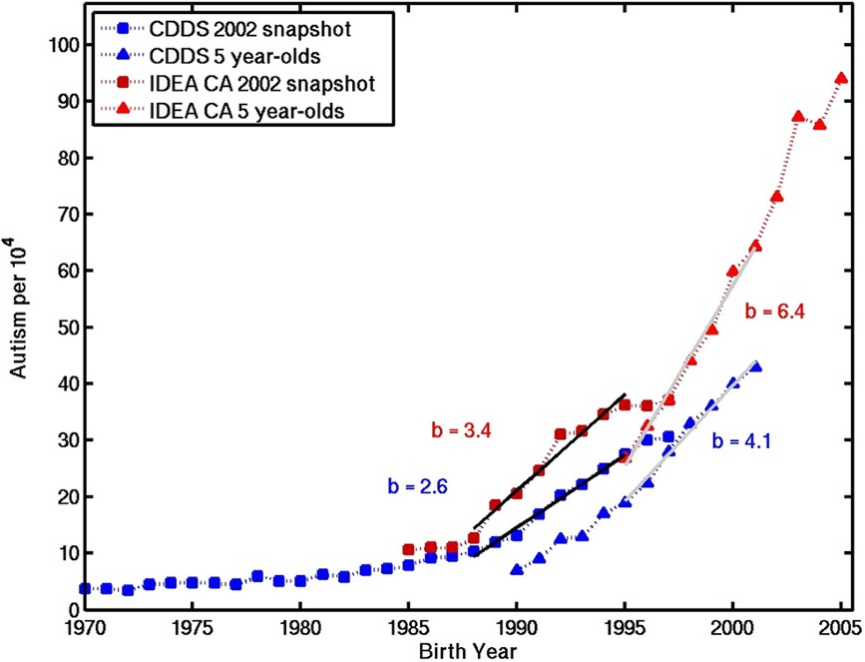
**Temporal trends in CDDS autistic disorder (blue) and California IDEA autism data (red).** For both CDDS and IDEA, age-resolved snapshots for 2002 (squares) and constant-age tracking data for 5 year-olds (triangles) are shown. Trends slopes (symbol b, in units of autism count per 10^4^ per year) are approximated using least squares linear regression over birth year intervals 1988–1995 (for 2002 snapshots) and 1995–2001 (for 5 year-old tracking).

To guide the eye in evaluating trends in suspected environmental toxins, a monotonic "composite" curve was constructed by combining CDDS 2002 snapshot data for birth years 1970–1994 with California IDEA 5 year-old tracking data for birth years 1995–2005. These datasets blend continuously, albeit for somewhat fortuitous reasons, described in the Results section below. The primary motive for combining them is to allow examination of concurrent trends in toxins and autism over the longest birth year window possible, 1970–2005 (Figure 2). The California data were assumed to be broadly representative of the rest of the United States, an assumption supported below by the results in Table 1.

**Table 1:** **Comparison of snapshot and constant-age tracking trends in IDEA autism prevalence for 7 to 10-year intervals beginning in birth years 1988 (interval 1) and 1993 (interval 2)**

Interval 1 (snapshot based on 2005 report)					Interval 2 (snapshot based on 2010 report)						
***b***_***snap***_	***b***_***track***_		Birth	N	***b***_***snap***_	***b***_***track***_		Birth	N	Track	Min.
***10*** ^***-4***^ ***y*** ^***-1***^	***10*** ^***-4***^ ***y*** ^***-1***^		Year		***10*** ^***-4***^ ***y*** ^***-1***^	***10*** ^***-4***^ ***y*** ^***-1***^		Year		Age	Snap
			Span					Span			Age
4.1 ± 1.5	5.3 ± 2.1	78 ± 8	1988-1997	24	6.3 ± 2.1	8.6 ± 2.6	73 ± 8	1993-2002	19	8	8
4.4	5.4	82		1	6.8	8.7	78		1		
4.2 ± 1.6	5.6 ± 2.2	77 ± 8	1988-1996	22	6.2 ± 2.2	8.5 ± 2.7	73 ± 8	1993-2001	22	9	9
4.5	5.5	81		1	6.9	8.7	80		1		
4.6 ± 1.6	6.0 ± 2.2	78 ± 7	1988-1995	21	6.6 ± 2.3	9.0 ± 2.9	74 ± 8	1993-2000	18	10	10
4.7	5.7	81		1	7.0	8.8	80		1		
5.2 ± 1.8	6.7 ± 2.4	77 ± 8	1988-1994	17	7.5 ± 2.9	9.8 ± 3.4	76 ± 8	1993-1999	11	11	11
4.8	6.0	81		1	7.0	8.6	81		1		

Snapshot slopes for interval 1 and 2 are based on the 2005 and 2010 IDEA reports, respectively. Slopes (symbol *b*, in units of # 10^-4^ y^-1^) are estimated based on least squares linear regression. The bottom line (N = 1) in each age pair shows results for California. The top line shows the mean and standard deviation for all states (N = 11 to 24) for which the error in the constant-age tracking slope is < 10% and the error in the snapshot slope is < 15%. The snapshot:tracking slope ratio is reported as a quantitative estimate of the fraction (in%) of the constant-age tracking slope that is "real" rather than due to better and expanding diagnosis. A range of tracking ages from 8 to 11 is considered.

The composite curve and the temporal trend in each of the suspected causal agents were compared by visual inspection. The correlation coefficient also was computed between the temporal trend in each suspected agent and the composite autism prevalence curve using the longest overlapping time period possible (Additional file 1: Table S1). However, these quantitative statistics are not emphasized in the Results and Discussion, due to the multiple uncertainties involved in defining the long-term trend in autism, discussed below.

### Suspected environmental factors

Guided by the recently published top ten list, an extensive literature and data search was conducted of available trends in each suspected environmental agent over the relevant time period overlapping with the CDDS and IDEA autism data. Priority was given to datasets with high temporal resolution that measured levels of suspect chemicals directly in bodily fluids or tissue (blood, urine, breastmilk and adipose tissue) of American women or young children. Data from the National Health and Nutrition Examination Survey (NHANES) were favored in particular. NHANES is an ongoing survey of chemicals in bodily tissues and fluids that is designed to reflect a nationally representative sample of the U.S. population over a number of years using a consistent sample design and consistent methods of measurement. NHANES data were found for lead, total blood Hg, BPA, phthalates and PFCs [27–30]. Bodily fluid or tissue data from other North American surveys and literature compilations were found for the organochlorine pesticide DDT and for dioxins, whose trends may offer some insight into PCB trends since dioxins were created largely as a byproduct of PCB manufacture [31, 32]. In cases where human bodily fluids were not available or applicable, other data sources were used, including concentrations in fish, product consumption records, pollutant emission estimates, atmospheric measurements, and historical vaccine schedules (http://www.cdc.gov/vaccines/schedules/past.html#prior-childhood) cross-matched to data on the mercury and aluminum content of each vaccine [33–35]. In the case of PFCs and endocrine disruptors, the limited available U.S. data were supplemented with more extensive data from Germany and Sweden [36–38], although the European trends were not necessarily assumed to represent U.S. trends. Finally, while not strictly speaking a toxin related to the top 10 list, obesity among American women of childbearing age [39] was also included in the trend analysis. All data sources are described in detail in the Additional file 1.

## Results

### Temporal trend in U.S. autism

The slope ratios for the California IDEA age-resolved snapshot and constant-age tracking trends suggest that about 80% of the tracked increase in California autism is "real" as opposed to due to better diagnosis, both for birth year intervals 1988 to 1994–1997 and 1993 to 1999–2002 (Figure 1). These results are relatively insensitive to the choice of tracking age, within the range of 8 to 11 (Additional file 1: Figure S2a, Table 1). The California results are similar to and within the standard deviation of the mean results across the United States (Table 1). The latter suggest that 77 ± 8% of the tracked increase over birth years 1988 to 1994–1997 is real and that about 74 ± 8% of the tracked increase over birth years 1993 to 1999–2002 is real.

In the above calculations, the birth year period from 1988 to 1994–1997 was the earliest interval used to derive a linear trend. Attempts were made to calculate linear tracking slopes beginning as early as 1985, but the error in those slopes exceeded 10% for all but a few states, suggesting that temporal trend could not be approximated well with a linear fit. This problem was traced back to an upward inflection in the IDEA constant-age tracking data around birth year 1988 (evident in Figure 1 for California and in many other states in Additional file 1: Figure S1). This inflection is also evident in the 2002 age-resolved snapshot for California and a number of states (Additional file 1: Figure S1). However, the slope errors of the snapshot-based trends for most states were less sensitive to the use of 1985 instead of 1988 as a start year than were the slope errors of the constant-age tracking trends. The snapshot data were more variable in general, and for this reason a slightly looser standard (slope error < 15%) was applied to them for inclusion in Table 1. The slope errors of < 10% (tracking) and < 15% (snapshot) were used as criteria to filter out states with erratic data and to identify those for which the temporal trend could be approximated well as a linear slope.

Comparison of the slopes of the 2002 age-resolved snapshots for CDDS and California IDEA data shows that IDEA has a steeper trend than CDDS (3.4 vs. 2.6 per 10^4^ yr^-1^) over the overlapping 1988–1995 birth year interval (Figure 2). The absolute IDEA numbers are also about 40% higher on average than CDDS. Comparison of the CDDS and IDEA 5 year-old tracking slopes over the overlapping1995-2001 birth year interval shows that the IDEA trend is more than 50% larger than the CDDS trend (6.4 vs. 4.1 per 10^4^ per year) and that the absolute IDEA prevalence is again about 40% higher on average than CDDS prevalence. Comparison of the two CDDS curves in Figure 2 for the overlapping 1990–1995 birth year interval shows that the CDDS 2002 snapshot data are about 40% higher on average than the CDDS 5 year-old tracking data, suggesting under-ascertainment among the 5 year-olds. Under-ascertainment among 5 year-olds relative to 10 year-olds is also evident in IDEA constant-age tracking data for all states in Additional file 1: Figure S1.

### Trends in suspected environmental factors

Most of the suspected environmental factors examined have flat, decreasing, or mixed but recently decreasing trends. A relative few stand out as having increasing trends that are positively correlated to varying degrees to the rise in autism. A summary of the temporal trends over the 1970–2005 time frame of the autism data is as follows:

#### Trends decreasing

Lead, PCBs, dioxins, organochlorine pesticides, vehicular emissions, air pollution, PAHs.

#### Trends mixed but recently decreasing

Organophosphate pesticides, PFCs, postnatal vaccine thimerosal. These compounds increased during the early part of the 1970–2005 period but began decreasing at some point during the later period.

#### Trends flat

Phthalates, atmospheric Hg, total blood Hg. These compounds have relatively flat temporal trends over the time period of available data (dating back only to the early to late 1990s for the U.S.).

#### Trends inconclusive

BPA. For BPA, German data suggest a decreasing trend since about 1997 but U.S. data are only available since 2003 and were therefore considered inconclusive (Additional file 1: Figure S15).

#### Trends increasing

Polybrominated diphenyl ethers (PBDEs), cumulative aluminum adjuvants, cumulative total immunizations, glyphosate, maternal obesity.

**Figure 3:**
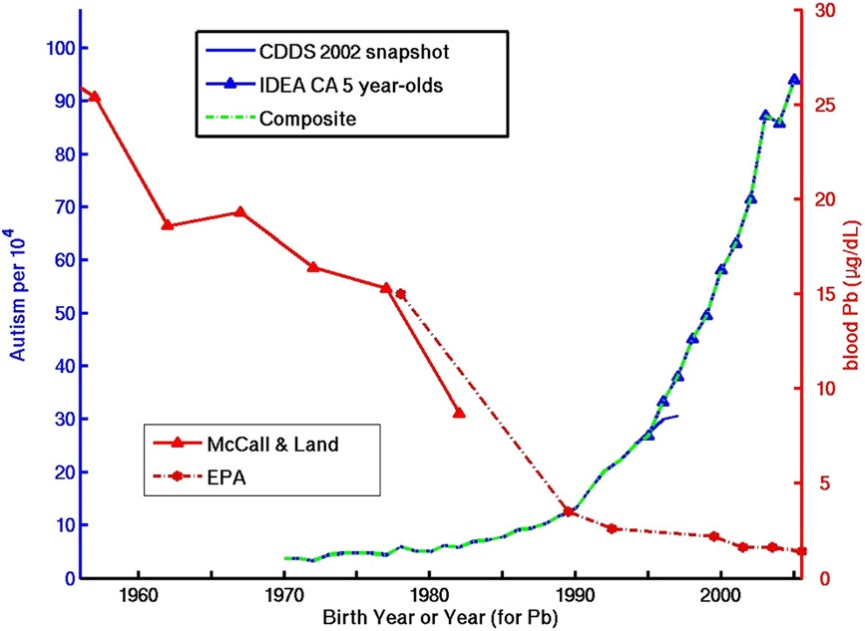
**Temporal trend in blood lead (Pb) concentration in U.S. children compared to the temporal trend in autism, constructed from a composite of CDDS 2002 snapshot data (covering birth years 1970–1997) and California IDEA 5 year-old tracking data for birth years 1995–2005 (see Section** **Method** **s for details).**

**Figure 4:**
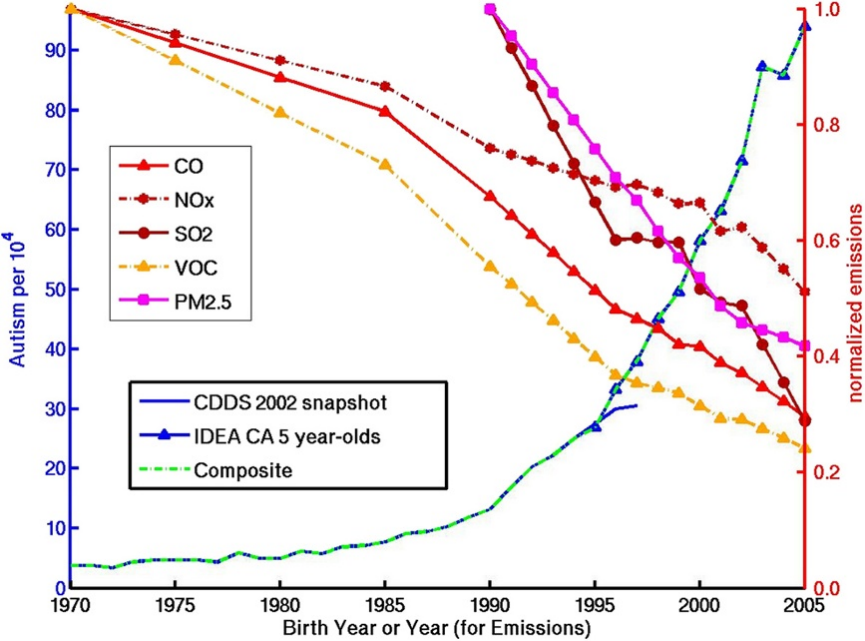
**Temporal trend in autism compared to trends in highway sector emissions of direct PM2.5 and indirect PM2.5 and ozone precursor species.** Emissions are normalized to the 1970 values for CO (=163 Mtons), NOx (=13 Mtons), and VOCs (=17 Mtons) and to the 1990 values for SO2 (=0.5 Mtons), and direct PM2.5 (=0.3 Mtons).

**Figure 5:**
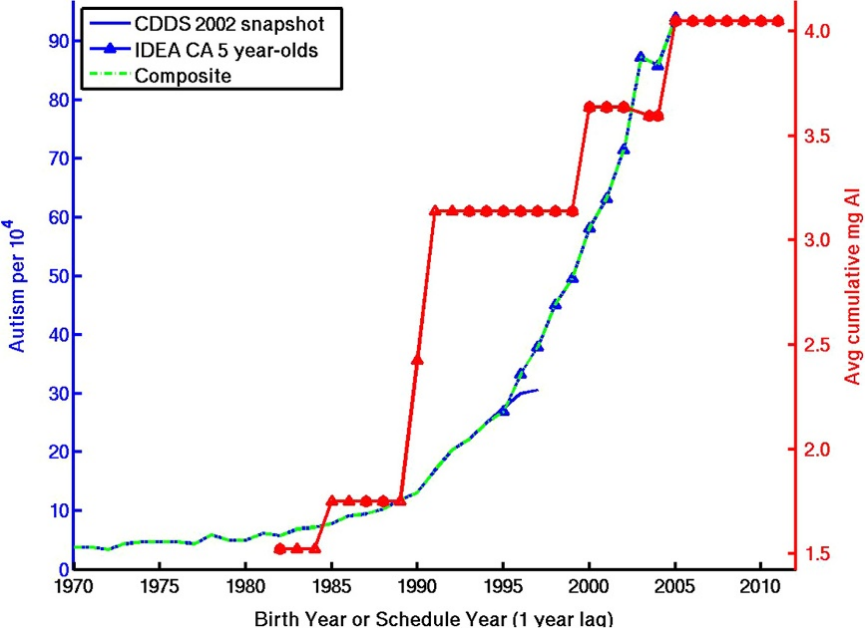
**Temporal trend in autism compared to temporal trend in cumulative amount of postnatal aluminum adjuvant administered to U.S. children by 18 months of age.** Red circles are years with published immunization schedules. Red triangles reflect educated guesses (see Additional file 1 **)** for details) in gap years without published schedules. The red curve is lagged 1 year because 18 month-olds born, e.g., in 1994 will likely follow the 1995 schedule.

**Figure 6:**
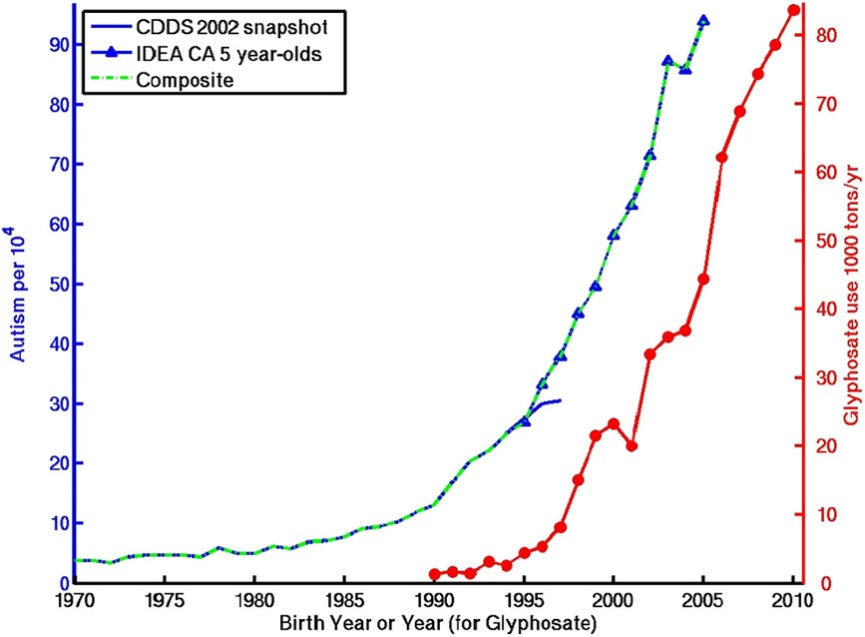
**Temporal trend in autism compared to temporal trend in U.S. application of glyphosate to genetically-modified corn and soy crops, as estimated from US Department of Agriculture data (see Additional file** 1 **).**

Additional file 1: Table S1 presents the correlation coefficients for the temporal trends in the suspect environmental factors vs. the composite 1970–2005 CDDS + IDEA record as well as the 1970–1997 CDDS-only record. Figures 3, 4, 5, 6 show dual Y-axis plots of the temporal trends in autism juxtaposed against the trends in lead, highway emissions, aluminum adjuvants and glyphosate, respectively. Plots for all the remaining environmental factors are shown in the Additional file 1. Several of the suspected environmental factors, including vehicular emissions/air pollution, mercury and vaccines, and organophosphate pesticides, are discussed below in more detail.

## Discussion

### Temporal trend in autism

This paper is built around the premise that the sharp increase in autism seen in constant-age tracking data over recent decades logically must be driven by a corresponding increase either in a single environmental exposure or in the collective influence of multiple environmental exposures. Critical to this whole premise is the following question: is the tracked increase in autism prevalence real or is it simply the result of better and expanding diagnosis? This question was addressed empirically by comparing autism trend slopes derived by tracking children of a constant age across multiple, successive annual IDEA reports to trend slopes derived from age-resolved snapshots from individual, recent IDEA reports in 2005 and 2010. It was assumed that by the time the recent reports were published, the greater awareness and expanding diagnosis of autism would have been retroactively applied to older children, who are still entitled until age 21 under IDEA to valuable educational services [40]. Given this assumption, it was hypothesized that a snapshot-based prevalence vs. birth year curve would have a flatter slope than a constant-age tracking curve and conceivably would be a completely flat line if autism is truly a constant-prevalence condition.

The comparison of snapshot and constant-age tracking slopes in the Results section provides partial support for the above hypothesis. In nearly all states, the snapshot slopes are flatter than the tracking slopes, indicating an ongoing, retroactive expansion in the diagnosis of autism among older students who were not identified as younger children (Figure 1, Additional file 1: Figure S1). This ongoing expansion is also evident in plots following specific birth year cohorts as they age (Additional file 1: Figure S2b). Overall, however, the IDEA data do not support the hypothesis that autism is a constant-prevalence condition, since both the age-resolved snapshots and the constant-age tracking data show a strong and largely consistent upward trend (Figure 1). On average across the United States, the snapshot:tracking slope ratios suggest that about 75-80% of the tracked increase in autism starting in the late 1980s is real (Table 1).

Another important result, derived by comparing California IDEA and CDDS data, is that California probably includes at least some milder ASDs in its IDEA autism category, despite reports to the contrary [40]. This result indicates that the IDEA definition of autism in California has expanded from full syndrome Autistic Disorder (the only ASD covered by CDDS) to include some milder ASDs and that the expanded definition leads to a stronger temporal trend. This finding for California raises questions about whether some of the other 30 states that in principle are ASD-exclusive also in practice may include milder ASDs under IDEA.

On the other hand, the IDEA data do not appear to include all ASDs. This can be inferred by comparing IDEA autism prevalence for 8 year-olds born in 2002 to recently published ADDM autism prevalence data for the same birth cohort [2]. ADDM data include the full spectrum of ASDs, including Asperger’s. Among the 11 ADDM sites reporting, the ADDM prevalence [2] is on average 74% higher than the IDEA prevalence calculated here (Additional file 1: Figure S2c). For example, the prevalence values for Utah and New Jersey are 0.7% and 1.3%, respectively, for IDEA compared to 1.9% and 2.2% for ADDM [2]. While these differences may occur in part because ADDM sampled only selected urban counties within these states whereas IDEA surveyed the entire state, it is also probable that the IDEA counts exclude some of the milder ASD cases. Due to the uncertainty over which milder ASDs are included in IDEA data and how this varies in different states, this paper is deliberately vague in its use of the term "autism" when discussing IDEA data.

The quantitative analysis of trends in IDEA autism presented here can be compared to two other published analyses, both of which were based on CDDS data. First, a recent examination of CDDS constant-age tracking data concluded that the upward trend in autistic disorder (AD) for birth years 1990–2003 was at least partly real, although likely also due in part to changing diagnostic criteria and younger age at diagnosis [5]. The quantitative details of that study imply that more than half (i.e., a fraction 4.2/7 to 4.2/8 or 52-60%) of the tracked increase in AD in CDDS data may not be "real" but rather due to changes in diagnostic criteria, the inclusion of milder cases, and an earlier age at diagnosis. Those factors were found to account for 2.2, 1.56, and 1.24-fold increases, respectively, or a combined 4.2-fold increase, which in turn was divided by the 7 to 8-fold tracked increase in AD from 1990–2003. However, the factor 1.56 due to inclusion of milder cases was, in that study’s own words, a "worst case" scenario that actually may be closer to 1 [5]. Similarly, the factor 2.2 ascribed to changes in diagnostic criteria was based on a Finnish study comparing diagnoses using the Diagnostic and Statistical Manual of Mental Disorders (DSM) IV versus the original Kanner definition [5], whereas the relevant comparison for the time frame in question arguably is between DSM IV and DSM III-R. DSM IV, published in 1994, introduced Asperger’s syndrome and the concept of autism as "spectrum" of disorders (ASDs), which include AD, PPD-NOS and Asperger's, but actually restricted the definition of AD relative to DSM III-R (published in 1987) [4]. From this perspective, the factor 2.2 might actually be closer to 1 or even less than 1. Thus, the remaining "non-real" fraction of the increase in AD, due to younger age at diagnosis, may be only 1.24/7 to 1.24/8 (16% to 18%), suggesting that up to 82-84% of the increase is real. This latter value is consistent with the analysis of California IDEA data presented here, which suggests that about 80% of the tracked increase among 8 to 11 year-olds over a similar time frame (1988–2002) is probably real. It also should be noted that the 80% real fraction deduced here for California may be an upper limit, since it is possible that some of the older children in the 2005 and 2010 IDEA age-resolved snapshots were never reevaluated for autism and thus remained undiagnosed despite the increased awareness in recent years.

Second, a recent mathematical analysis of the CDDS 2002 snapshot data identified 1988–1989 as the inflection point in the curve when autism prevalence started its sharp rise [3]. (Notably, CDDS prevalence already had been rising more gradually since about 1980, doubling from 5 to 10 per 10,000 by 1988 [3].) The 1988–1989 inflection point is consistent with the current analysis of IDEA data, which found relatively large slope errors when trying to fit a linear trend to IDEA data beginning prior to about birth year 1988. While proving the 1988–1989 change point is beyond the scope of the current study, the existence of an identifiable inflection point in the autism trend data is important, because it would tend to argue against diffuse intergenerational epigenetic explanations and would suggest instead that the temporal drivers of autism may be fairly specific. Although many different toxic exposures may contribute to oxidative stress and inflammation and thus may be identified as statistically significant risk factors for autism in epidemiological studies, the existence of an inflection point would suggest the value of considering which environmental factors could be driving a steep and ongoing increase in autism prevalence beginning circa 1988–1989.

### Air pollution

Recent epidemiological studies have found that autism is associated with ambient exposure to ozone and PM2.5 during pregnancy [41] as well as with birth residence proximity to freeways but not major roads [23]. This latter result suggests a connection to large diesel trucks, which travel more often on freeways than surface streets. It may also implicate ultrafine or nanoparticles, whose number concentration is high near freeway traffic, but falls off exponentially away from the freeway due to atmospheric dilution, coagulation and other loss mechanisms [42, 43]. While large diesel truck miles traveled have increased 4-fold from 1970 to 2005 [44], the increase in miles appears to be overwhelmed by larger reductions in emissions per mile for key pollutants [45]. Estimated vehicular emissions of the carcinogenic PAH benzene α pyrene (BaP) show a strongly decreasing trend that is anticorrelated to trends in autism (Additional file 1: Figure S16a). The emission factors for 8 other PAHs, as well as for CO, VOCs and particulate organic carbon, show a similar decreasing temporal trend [45]. These decreases are supported by United States Environmental Protection Agency (USEPA) estimates of highway emissions of 5 major pollutants contributing either directly or indirectly to PM2.5 and ozone formation (Figure 4), which have decreased by ~50-75% from their reference values, available from either 1970 or 1990 [46]. The trends in highway emissions parallel decreasing trends in total emissions of these pollutants from all sectors [46, 47].

The large drop in vehicular emissions occurred mainly by the 1980s and is attributed to the introduction of catalytic converters in the 1970s and ongoing improvements in fuel and emissions technology. Emissions of black carbon, which are closely associated with diesel fuel combustion and large trucks, also appear to be dropping significantly, thanks to improved technology such as diesel particle filters [48]. However, a counter trend toward increasing emissions of nanoparticles, a subset of PM2.5 that generally is not resolved by routine measurement techniques [42], cannot be ruled out, although a literature search turned up no articles indicating such a trend.

Direct measurements of air pollution provide an integrated metric of the effect of vehicular and other emissions on the atmosphere. Ozone and PM2.5 are two of the most widely monitored air pollutants and both recently have been linked to higher rates of autism in Los Angeles [41]. However, EPA 8-hour ozone standard violations in Los Angeles as well as 10 other major U.S. cities show flat or downward trends that correlate poorly to the rise in autism (Additional file 1: Figure S17). Similarly, PM2.5 levels in Los Angeles and 3 other major cities in states with some of the highest ASD prevalence also show flat or downward trends (Additional file 1: Figure S18). While the ozone violation and PM2.5 time series shown in these figures are available only from 1995 and 2000, respectively, studies taking a longer view confirm that the U.S. has achieved significant reductions in ozone since the U.S. Clean Air Act was established in 1970. Across the United States on average, ozone has decreased by 28% since 1980 [47]. In the Los Angeles basin, maximum 8-hour average ozone levels have decreased by a factor of 3 between 1973 and 2010 [49].

In summary, there is no obvious evidence to suggest that trends in estimated vehicular emissions or directly measured air pollution are consistent with the sharp temporal increase in U.S. autism. It is therefore intriguing that vehicular emissions and air pollution have been associated with autism in multiple epidemiological or ecological studies [23, 41, 50–52]. While air pollution, and nanoparticles in particular, can create metabolic conditions that are consistent with some of the biochemical imbalances seen in autism [53–55], the inverse trend relationship suggests the need for a coherent theory of how air pollution may interact with as yet unidentified temporal drivers to explain the increase in U.S. autism.

### Mercury and vaccines

It has been hypothesized that autism is a form of mercury poisoning, based on the similarities between known symptoms of mercury poisoning and the behavior traits and biological abnormalities of autistic children [56–58]. In the original hypothesis, the vaccine preservative thimerosal was suggested as the main relevant route of exposure [56]. Additional file 1: Figure S6 shows that the expansion of thimerosal exposure in the late 1980s and early 1990s coincides closely with the rise in autism around that time. However, as noted by others [26], the temporal trends in autism and thimerosal following the childhood vaccine thimerosal phaseout are incompatible. Postnatal thimerosal therefore seems unlikely to be driving the ongoing increase in autism in the 2000s, although a recent reported decrease in the severity of ASD among younger birth cohorts may coincide with the thimerosal phaseout [2].

A possible confounding factor in the postnatal thimerosal analysis is the administration of flu shots to pregnant women, which increased in the late 1990s/early 2000s around the same time that thimerosal was being phased out of children’s vaccines. Many flu shots still contain 25 μg Hg and thus may be leading to increased prenatal exposure. Anti-D Immune Globulin products, which contained up to 65 μg Hg per dose in the 1990s, were another prenatal source of thimerosal. Beginning in 1991, these shots were recommended routinely for RH- pregnant women (about 11% of the population), who often received two or more doses during their pregnancy [59]. However, thimerosal was removed from these immune globulin products around 2001, creating a competing trend in prenatal exposure from that due to flu shots. An additional complication is that the relative impact of prenatal and postnatal thimerosal is difficult to compare quantitatively, due to uncertainties in the degree of protection provided by the mother and in the sensitivity in the timing of fetal development to Hg [60].

Other vaccine indices, including cumulative aluminum adjuvants and cumulative total number of immunizations, continue to correlate strongly with autism trends (Figure 5, Additional file 1: Figure S7-S8). Aluminum is a demonstrated neurotoxin that can induce neuroimmune disorders and cellular oxidative stress [61, 62]. Several recent studies have described biological mechanisms by which aluminum could contribute to autism and have emphasized the need to consider the interaction of aluminum and vaccines with other pharmaceuticals, including antibiotics and the antipyretic acetaminophen [34, 63–66]. The upward trend in aluminum adjuvant exposure is also notable in that very young infants have experienced the largest relative increases from the early 1980s to 2005. Newborns have seen essentially an infinite increase due to the hepatitis B birth dose, the receipt of which has been linked epidemiologically to increased autism risk [67], while 2 month-olds have seen about a 3-fold increase in aluminum adjuvant exposure (range 2.5 to 5.7, depending on the Al content assumed for DPT and DTaP, which varies widely among different manufacturers [33]) (Additional file 1: Figure S7b). However, with the exception noted above, most epidemiological studies have found no correlation between vaccines and autism, although these studies have focused specifically on either thimerosal or the MMR vaccine rather than on aluminum [35, 68, 69].

The remaining Hg trend investigations below focus on prenatal exposure, since mercury is known to be particularly harmful to the developing fetus and to concentrate by about a factor of 2 in cord blood relative to maternal blood [70]. Total blood Hg provides a direct, integrated measure of recent mercury exposure from a variety of influences including diet, dental amalgams, thimerosal and atmospheric pollution. Within the time frame of available U.S. data (1999-present), the blood Hg trend is flat and shows little evidence of a sharp increase in recent years among women of reproductive age. At a mean value of 0.8 μg/L, U.S. women’s blood Hg levels are also relatively low compared to other countries such as Japan, South Korea and Sweden [71–73]. A final notable feature of the U.S. blood data is the tendency toward increasing Hg levels with advancing age (Additional file 1: Figure S3) [27].

Since the available blood Hg data were limited to the final seven years of the autism record, additional data sources were explored to try to reconstruct earlier trends. Consumption records of seafood and high fructose corn syrup provide some indication of trends in dietary Hg exposure. However, they are weaker indices than direct blood measurements, since exposure also depends on trends in the Hg content of these products, which this study was unable to resolve.

Seafood is one of the most important sources of human Hg exposure, since MeHg can bioaccumulate in higher trophic level fish. Fish are also a good source of poly-unsaturated fatty acids, selenium and Vitamin D, all of which have beneficial effects on neurological function that may help counter the harmful effects of mercury [74, 75]. Total U.S. seafood consumption has increased 40% since 1970, but consumption of pelagic fish, including tuna and large fish with highest MeHg content, has declined since 1990 (Additional file 1: Figure S4). The above results appear consistent with previous findings that women may be shifting away from high MeHg species even as their total fish intake increases [76], suggesting a relatively flat tend in MeHg exposure.

High fructose corn syrup (HFCS) is another source of dietary mercury, with an upward trend in consumption that was moderately well correlated to trends in autism during the 1980s and 1990s, although current Hg exposure through HFCS is declining (Additional file 1: Figure S5). Using high-end Hg content estimates, the mean consumption of 12 μg Hg/day via HFCS in 2005 corresponds to a substantial annual intake of 4400 μg Hg/year. This is comparable to the amount of MeHg ingested via seafood at the U.S. per capita consumption rate of about 24 kg/yr, assuming a mean content of ~ 0.2 ppm. Unlike fish, which contain mitigating nutrients, HFCS is associated with highly processed, nutrient-poor diets that can contribute to autism risk factors such as zinc deficiency and obesity [17, 75, 77]. However, the wide range of uncertainty in the Hg content of HFCS makes it difficult to quantify the exact temporal trend in mercury exposure.

Atmospheric Hg is an additional exposure that has been linked to autism [50–52] and is essentially a ubiquitous, unavoidable source. Gaseous Hg(0), the dominant form of atmospheric mercury, is considered toxic if inhaled because it can directly enter the blood stream from the lungs. However the concentration of Hg(0) in air is low [78] at about 1.5-2 ng/m^3^, such that the typical amount inhaled is about 0.02 μg Hg/day for U.S. adults. This a factor of 10^3^-10^4^ less than the MeHg ingested in a single serving of tuna. Further, in Europe and North America, improved emissions controls on coal plants and other major emitters have led to substantial declines in anthropogenic Hg emissions in recent years. In response, atmospheric Hg concentrations and deposition rates have stabilized over the U.S. in the last two decades, although they have not actively declined [79, 80] (Additional file 1: Figure S9a). Meanwhile, atmospheric concentrations appear to be declining at several remote monitoring sites [78] (Additional file 1: Figure S9b). These trends may reflect competing influences from the ongoing expansion of coal combustion in Asia, improved emissions controls in Europe and North America, and changes in natural and "legacy" emissions from the large reservoir of anthropogenically mobilized Hg now residing in the earth’s crust and surface ocean [81]. Considering the flat trends and small doses described above, it seems unlikely that atmospheric Hg can be driving the U.S. increase in autism.

### Organophosphate pesticides

Epidemiology has linked ASD and PDD in children to both prenatal and postnatal exposure to cholinesterase-inhibiting organophosphate (OP) insecticides [24, 82]. Further, the biological plausibility of these insecticides as a cause of autism has been described and wheat and corn have been identified as the most important sources of OP exposure among U.S. children [19]. However, the temporal trend in total OP insecticide use does not correlate well to the trend in autism. According to USEPA and USDA data, total agricultural use of OP insecticides on 5 major crops (including corn, wheat, potatoes, cotton and soy) declined about 30% between 1995 and 2005 (Additional file 1: Figure S12a) [83]. An important reason for the decline in OP insecticide application to corn, cotton and potatoes was the adoption of crops genetically modified to produce Bt toxin, which repels targeted insect pests, thus reducing the need for external insecticides. However, the combined 5-crop dataset does not resolve how the shift to GM crops has affected OP insecticide application specifically to wheat over the 1970–2005 time frame.

In addition to the 5 major crops, USEPA data showing declines of ~50-75% in organophosphate residues on apples, grapes, carrots and tomatoes from 1998–2000 to 2007–2009 suggest that use is also declining on fresh fruit and vegetable crops [29]. The reasons for the decline in fruit and vegetable residues are not stated in the USEPA report, and the substitution of other pesticides for OP cannot be ruled out. Along with the decline in agricultural use, chlorpyrifos, an OP insecticide commonly used in household applications, was banned for residential use by the USEPA in 2001. Chlorpyrifos concentrations have subsequently declined in urban streams and rivers in the northeastern and midwestern United States [84]. However, other OPs continue to be used in household applications, e.g., as pet flea products, with temporal trends that are not resolved by this study. Total insecticide use and herbicide use appear to have flat or slightly declining trends from about 1980 through 2006 [85] (Additional file 1: Figure S12b,c).

An exception to the overall modest decline in U.S. pesticide use is the rapidly increasing application of glyphosate, the active ingredient in the herbicide Roundup® (Figure 6). Glyphosate is applied widely to genetically modified crops, including corn, soybean, cotton, canola, sugar beets and alfalfa. While glyphosate has the basic chemical structure of an organophosphate pesticide, it is not a conventional cholinesterase-inhibiting insecticide. Rather, its mechanism of toxicity involves the disruption of the shikimate pathway needed in the synthesis of essential aromatic amino acids in plants. This pathway is used by human gut bacteria, which play an important role in the immune system and are often compromised in autistic children [86]. An additional biochemical connection is that the metabolism of glyphosphate depends on glutathione, which is significantly depleted in autistic individuals [87, 88].

From a temporal trends perspective, glyphosate was first created in the 1970s, whereas the first reported cases of autism occurred in the 1930s [1]. Further, its widespread use did not begin until the mid 1990s, well after the beginning of the surge in autism diagnoses in the late 1980s. In recent years, however, its temporal trend closely tracks the ongoing increase in autism. Based purely on these timing considerations, it appears that glyphosate cannot be responsible for the first autism cases in the 1930s and is unlikely to have caused the late 1980s uptick, but could be interacting in recent years with other toxins to drive up the prevalence of U.S. autism.

### Final thoughts and considerations

Correspondence between temporal trends in autism and environmental factors is a useful method for identifying possible triggers of autism to help focus future research. However, it must be emphasized that the correlation in temporal trends between autism and PBDEs, cumulative aluminum adjuvants, and glyphosate shown here is not proof of causation, especially given the ecological nature of this study, in which the exposure data were aggregated at the group level. Only application of a comprehensive set of criteria for assessing causation [89] combined with a deeper understanding of the underlying biology and epidemiological evidence correlating individual-level exposures and outcomes can prove whether a suspect compound or trigger is a likely cause. On the other hand, the strongly incompatible temporal trends in some named suspects, particularly those banned or sharply curtailed in the 1970s, such as lead, PCBs, and organochlorine pesticides, make these compounds less likely drivers of the rapid increase in autism since the late 1980s. However, this study only examined the trends in a small subset of the thousands of environmental chemicals in current use and cannot rule out that the sheer volume of all these toxins is converging to drive the autism increase.

It is also possible that the drivers of the temporal trend in autism are tied into the factors responsible for the rise in other autoimmune or hyperimmune system diseases such as asthma, Crohn’s disease, lupus, and type 1 diabetes. All of these diseases have increased in recent decades in the U.S as well as in many other countries. The rise in these autoimmune conditions has been attributed to increased systemic inflammation, driven in large part by changes in the intestinal biome in the postindustrial era and the loss of microorganisms that helped regulate the human immune system in our evolutionary past [90]. Modern, western-style, high calorie/low nutrient diets and related obesity also can alter gut microbiota and contribute to chronic inflammation and oxidative stress, creating an upward temporal trend in the metabolic conditions that increase vulnerability to immune/inflammatory response [91, 92]. These conditions can affect fetal development and, indeed, maternal obesity has been associated with increased risk of autism [77]. Notably, Additional file 1: Figure S21 shows that the time trend in obesity among U.S. women correlates well to that of autism, suggesting maternal obesity may be a direct influence or a comorbid consequence of the dietary factors contributing to autism, or both.

A literature survey of trends in other autoimmune conditions suggests that they do not appear to be rising at the same rapid rate as autism. For example, asthma prevalence among U.S. children increased more or less linearly by (only) about a factor of 2 from 3.6% in 1980–1981 to 6.9% in 1995–1996 [93] and (using an altered metric) from 8.7 to 9.4% from 2001 to 2010[29]. Similarly, the rate of hospitalization in the U.S. for Crohn’s disease increased by a factor of 2 from 1990 to 2003 [94]. Finally, type 1 diabetes incidence among children in Colorado stayed flat at around 14.8 per 100,000 from 1978–1988, then increased by less than a factor of 2 to 23.9 per 100,000 by 2002–2004 [95]. In comparison, the composite trend constructed from CDDS and California IDEA data, suggests a more than 20-fold increase in autism prevalence between birth years 1970 and 2005, most (~80%) of which is probably real. Thus, while lifestyle factors related to modern diet and hygiene may be contributing to the rise in prevalence, autism stands out from the above auto and hyperimmune conditions in the strength of its temporal trend.

## Summary

Temporal trends in autism were constructed both by tracking prevalence at a constant age in a series of historical IDEA reports and by computing prevalence from age-resolved snapshots in individual, recent IDEA reports. Both the snapshot and tracking approaches suggest a strong increase in autism that took off in the late 1980s and was ongoing as of birth year 2005. The ratio of the snapshot:tracking slopes suggests that among states with the most reliable data, about 75 to 80% of the tracked increase in IDEA autism since 1988 is due to a real increase in the disorder rather than just to better or expanded diagnosis. The trend in California IDEA autism prevalence was shown to be broadly representative of the mean United States trend and was extended to span birth years 1970–2005 using a composite CDDS plus IDEA dataset. The composite dataset, which shows that a more gradual increase in autism had begun already by 1980, was compared to the corresponding trends in a list of suspected toxins and environmental influences. Several of these influences, including polybrominated diphenyl ethers, aluminum adjuvants, the herbicide glyphosate, and obesity among U.S. women, have increasing trends that are positively correlated to the rise in autism. However, most of the toxins surveyed, including lead, PCBs, organochlorine pesticides, vehicular emissions and air pollution, have flat or declining trends, making it less likely that they can be driving the increase in diagnosed autism seen over the 35-year period of the composite data set.

## Authors’ information

CN is an atmospheric and environmental research scientist at the University of Colorado, Boulder. She conducted this study as a volunteer for SafeMinds. The views expressed herein are her own and do not necessarily represent those of the organization.

## Electronic supplementary material

Additional file 1: Figure S1: shows temporal trends in IDEA autism prevalence in all 50 states plus D.C. For each state, data tracking 5 and 10-year olds are compared to age-resolved snapshots for 2002 and 2010. **Figure S2.** shows auxiliary information on the IDEA trend analysis. A text description of each suspected environmental influence is provided and the temporal trend is shown in a dual Y-axis plot juxtaposed against the trend in autism in **Figures S3-S21**. **Table S1.** summarizes the data used to construct the temporal trend in each environmental factor and its correlation with the autism trend. (DOCX 5 MB)

## Abbreviations

AD: Autistic Disorder
ASD: Autism Spectrum Disorder
BaP: Benzene α Pyrene
BPA: Bisphenol A
CDDS: California Department of Developmental Services
DSM: Diagnostic and Statistical Manual of Mental Disorders
DDT: Dichlorodiphenyltrichloroethane
HFCS: High Fructose Corn Syrup
IDEA: Individuals with Disabilities Education Act
MeHg: Methylmercury
PM2.5: Particulate Matter < 2.5 microns
PFCs: Perfluorinated compounds
PDD-NOS: Pervasive developmental disorder - not otherwise specified
PBDEs: Polybrominated diphenyl ethers
PCBs: Polychlorinated biphenyls
PAHs: Polycyclic aromatic hydrocarbons
TCDD: Tetrachlorodibenzo-p-dioxin.
