# Supplementary material for: A comparison of temporal trends in United States autism prevalence to trends in suspected environmental factors
Source: Environ Health. 2014 Sep 5;13:73. doi: 10.1186/1476-069X-13-73 (PMC4177682; doi:10.1186/1476-069X-13-73)
Supplement: Supplementary file 1 — Additional file 1: Figure S1: shows temporal trends in IDEA autism prevalence in all 50 states plus D.C. For each state, data tracking 5 and 10-year olds are compared to age-resolved snapshots for 2002 and 2010. Figure S2. shows auxiliary information on the IDEA trend analysis. A text description of each suspected environmental influence is provided and the temporal trend is shown in a dual Y-axis plot juxtaposed against the trend in autism in Figures S3-S21. Table S1. summarizes the data used to construct the temporal trend in each environmental factor and its correlation with the autism trend. (DOCX 5 MB) [file 12940_2014_781_MOESM1_ESM.docx]

**Supplementary Information**

**A comparison of temporal trends in U.S. autism prevalence to trends in suspected environmental factors.**

**Cynthia D. Nevison**

Institute for Arctic and Alpine Research, Campus Box 450, University of Colorado, Boulder, Colorado 80309-0450, [Cynthia.Nevison@colorado.edu](mailto:Cynthia.Nevison@colorado.edu), Tel. 303 492-7924.

**Table of Contents**

S1. IDEA Autism Trends for All States: Tracking vs. Snapshots 1

S2. Description of Data Sources 2

S2.1 Lead 2

S2.2 Mercury 2

S2.2.1 U.S. Blood Mercury Levels 2

S2.2.2 Fish Consumption 2

S2.2.3 High Fructose Corn Syrup Consumption 2

S2.2.4 Thimerosal and Vaccines 3

S2.2.5 Atmospheric Mercury 4

S2.3 Polychlorinated Biphenyls (PCBs) and Dioxins 5

S2.4 Organophosphate Pesticides 5

S2.5 Organochlorine Pesticides 6

S2.6 Endocrine Disruptors 7

S2.61. Phthalates 7

S2.6.2 Bisphenol A (BPA) 7

S2.7 Automotive Exhaust and Air Pollution 7

S2.8 Polycyclic Aromatic Hydrocarbons (PAHs) 8

S2.9 Polybrominated Biphenyl Ethers (PBDEs) 8

S2.10 Perfluorinated Compounds (PFCs) 9

References 10

Figure S1 State Autism Trends from IDEA 14

Figure S2 Auxiliary Information on IDEA Analysis 17

Figure S3 Women’s Blood Mercury 20

Figure S4 Seafood Consumption 21

Figure S5 Hg in High Fructose Corn Syrup 23

Figure S6 Cumulative Postnatal Thimerosal 24

Figure S7 Cumulative Postnatal Aluminum Adjuvant 26

Figure S8 Cumulative Total Immunizations 28

Figure S9a Hg Wet Deposition over Great Lakes, Northeast 29

Figure S9b Total Gaseous Mercury (TGM) in Northern Hemisphere 30

Figure S10 PCBs in Great Lakes Fish 31

Figure S11 TCDD in Serum and Adipose Tissue 32

Figure S12 Organophosphate and Total Agricultural Pesticides 33

Figure S13 DDT in Breast Milk 36

Figure S14 Phthalathes in German and U.S. Urine 37

Figure S15 BPA in German and U.S. Urine 39

Figure S16 Polycyclic Aromatic Hydrocarbons (PAHs) 40

Figure S17 Ozone in 11 U.S. Cities 42

Figure S18 PM2.5 in 4 U.S. Cities 45

Figure S19 PBDEs in Great Lakes Trout vs. United States ASD 46

Figure S20 Perfluorinated Compounds (PFCs) 47

Figure S21 Obesity in U.S. Women 49

Table S1 Summary of Correlations to Autism Trend 50

**S1. IDEA Autism Trends for All States: Tracking vs. Snapshots**

Figure S1 shows autism trends from the Individuals with Disabilities Education Act (IDEA) database ([www.ideadata.org](http://www.ideadata.org)), tracked among 5 year-olds and 10 year-olds (light and heavy red curves, respectively) in all 50 states plus the District of Columbia. IDEA data for 5 year-olds are available from 2000 to 2010, corresponding to birth years 1995-2005. IDEA data for 10 year-olds are available from 1991 to 2010, corresponding to birth years 1981-2000.

Figure S1 also shows the temporal trend in autism calculated independently from age-resolved snapshots using IDEA reports from 2002 and 2010 (cyan and blue curves, respectively). In both the constant-age tracking and snapshot approaches, the temporal trend was derived by plotting prevalence against birth year, which was calculated as *Birth Year = Report Year - Age*. In the tracking approach, *Age* was held constant while *Report Year* was varied from 1991-2010. In the snapshot approach, *Report Year* was held constant while *Age* was varied from 5 to 17 years.

To calculate prevalence, IDEA autism counts were divided by total statewide public school age populations by grade, matching to the appropriate IDEA age (e.g, kindergartners = 5 year-olds) and fifth graders = 10 year-olds) from the National Center for Education Statistics (NCES) (<http://nces.ed.gov/ccd/bat/>). As noted in Section 2.1, children in private schools are included in IDEA data but not in NCES data, such that the uncorrected ratio probably overestimates the actual prevalence. However, due to lack of sufficient information on private:public school population ratios for all 50 states dating back to 1991, Figure S1 does not attempt to correct for the missing private school component of the denominator.

Figure S2 provides several pieces of auxiliary information about the IDEA trend analysis. Figure S2a expands on Figure 1 by illustrating the sensitivity of the age-resolved snapshot and constant-age tracking trend slopes to differences in tracking age. Figure S2b samples California IDEA/NCES data across 20 years of reports (1991-2010) to follow specific birth year cohorts as they age, i.e., marching forward 1 year in age with each annual report. Finally, Figure S2c compares IDEA/NCES prevalence reported in 2010 among 8 year-olds (birth year 2002) to the corresponding prevalence in the 11 available states from the Autism and Developmental Disabilities Monitoring (ADDM) Network (read from Table 2 of *CDC*, 2014). Some possible reasons for the differences between IDEA/NCES and ADDM prevalence shown in Figure S2c are: 1) ADDM only sampled selected regions within the 11 states, whereas the IDEA/NCES numbers include the entire state, 2) ADDM includes the full spectrum of ASD, whereas IDEA may exclude some of the milder cases in some states, and 3) the IDEA/NCES numbers may be slightly inflated by the neglect of the private school population in the denominator, as described above.

**S2. Temporal trends in Suspected Causal Agents: Background Information and Data Sources**

**S2.1 Lead**

Lead was widely used in the last century in paint, solder for drinking water pipes and tin cans, and as an anti-knocking additive to gasoline. These uses were largely phased out by the 1970s. In particular, lead was phased out of gasoline in the United States starting in the early 1970s, leading to a sharp reduction in human exposure. Although levels are still high enough in some U.S. children to cause neurodevelopmental toxicity (Jusko et al. 2008), mean U.S. children’s blood Pb has dropped dramatically since the middle of the 20^th^ century (Figure 3). A composite children’s blood Pb curve was constructed extending from 1932 to 1999 using U.S. National Health and Nutrition Examination Surveys (NHANES) data on childhood geometric mean Pb levels, as compiled and averaged into broad 5-year 1932-1982 birth year bin averages by McCall and Land (2004). These were combined with median blood Pb for American children ages 1-5 compiled from NHANES data from 1976/1980 to 2009/2010 (USEPA 2013, Table B1).

**S2.2 Mercury**

**S2.2.1 U.S. Blood Mercury Levels**

Geometric mean total blood Hg levels (Figure S3) were obtained for U.S. children age 1-5, girls age 16-19, and women age 20-29 and 30-39 from 4 NHANES surveys spanning 1999-2006 (*Caldwell et al.*, 2009). Additional NHANES data were obtained through 2009/2010 for children age 1-5 and adult women age 16-49 (USEPA 2013, Tables B3 and B3c). Blood Hg data for U.S. women and children prior to 1999 were not found, despite an extensive literature survey. In addition to total blood Hg, U.S. NHANES data from 1999-2006 were found on trends in U.S. women’s inorganic blood mercury levels, which may be a better measure of chronic Hg exposure than total blood Hg (*Laks,* 2009). However, the latter were presented in unexplained units of μ/L and the reported levels hovered close to the instrumental limit of detection.

**S2.2.2 Fish Consumption**

Consumption of MeHg in fish is the single greatest source of mercury exposure for many people and rates of fish consumption tend to correlate to blood mercury concentrations in the U.S. population (*Mahaffey et al.*, 2008, 2009). U.S. per capita fish consumption data in kg per capita/year for 1961-2007 were obtained from FAOSTAT Food Balance Sheets, partitioned into pelagic, demersal (i.e., bottom-dwelling), freshwater, crustaceans and mollusks (faostat.fao.org) (Figure S4).

**S2.2.3 High Fructose Corn Syrup (HFCS) Consumption**

HFCS is a common ingredient in many processed foods and has been used since the 1960s as a sweetener to stabilize food products and enhance shelf life. HFCS is produced during the wet-milling of corn using a variety of chemicals, including caustic soda from mercury-cell chlor-alkali plants. As in seafood, the Hg content of HFCS can vary by several orders of magnitude, with levels ranging from below the level of detection (< 0.005 mcgHg/gHFCS) to up to 0.57 mcgHg/gHFCS in 20 samples tested (*Dufault et al.,* 2009a). The nondetectible levels likely came from factories using cell membrane chlor-alkali technology to make their caustic soda, and most manufacturers may have switched to this technology by the mid 2000s. Nevertheless, it seems likely than many consumers were unwittingly exposed to low levels of mercury over time through consumption of HFCS (*Dufault et al.*, 2009b).

The trend in Hg consumption via HFCS was estimated using gHFCS/per capita/day consumption data from the USDA (2013) multiplied by the Hg content of HFCS in mcgHg/gHFCS reported by *Dufault et al.* (2009a) (Figure S5). The minimum line is ~ 0 and reflects the fact that HFCS produced with cell membrane technology is largely uncontaminated with Hg. The mean (solid red) line shows the average of the (9 out of 20) samples with detectible Hg levels, while the maximum line (dashed red) reflects the maximum Hg content measured by Dufault *et al.*

**S2.2.4 Thimerosal, Aluminum and Vaccines**

The vaccine preservative thimerosal (roughly 50% ethyl mercury by weight) was first used in children’s diptheria (later DTaP) vaccines in the 1930s and, beginning in the mid 1980s to early 1990s in the toddler Hib, infant Hib and Hepatitis B (HepB) vaccines. Thimerosal was gradually phased out of these vaccines as a “precautionary” measure after it was realized that children, including newborns, were receiving Hg at levels one to two orders of magnitude above the EPA standard of 0.1 mcg Hg per kg body weight per day. It is not well known when the phaseout was completed, and the results likely varied by state and may have extended through 2003 in some cases. However, most children’s Hib, HepB and DTaP vaccines were probably thimerosal free by the end of 2001 (*Schechter and Grether*, 2008).

The time history of the cumulative amount of Hg administered to children via thimerosal at 0,1,2,4,6,12,15,18, 30, 42 and 48 months was constructed based on the Centers for Disease Control (CDC) recommended childhoold immunization schedules. These were obtained from [http://www.cdc.gov/vaccines/schedules/past.html#prior-childhood](http://www.cdc.gov/vaccines/schedules/past.html), and are available for 1983, 1988-1989, and 1994-2012. (These years are shown as filled large circles in Figures 5 and S6-S8.) In the case where a range of ages was listed for a vaccine dose, the earliest recommended age was consistently used, e.g., 12-15 months was counted as 12 months. Nearly complete uptake was assumed more or less immediately after the vaccines were added to the recommended schedule, although this is probably not a good assumption for HepB, whose uptake has increased more slowly than other vaccines according to World Health Organization data (WHO, 2012). The following educated guesses were made for the missing years 1984-1987 and 1990-1993 (shown as triangles in Figures 5 and open small circles in S6-S8):

1. HepB was added to the schedule in 1992 at 0,1, and 6 months

2. infant Hib was added to the schedule in 1991 at 2,4,6, and 12 months

3. toddler Hib was added to the schedule in 1986 at 18 months

4. MMR was moved from 15 months to 12 months in 1991

5. the MMR booster at 4-6 years was added in 1991

6. OPV3 was moved from 15 months to 6 months in 1991

Standard thimerosal contents of 12.5, 25 and 25 mcg Hg were assumed for each HepB, Hib and DPT or DTaP dose, respectively (*Verstraeten et al.,* 2003). Thimerosal was assumed to be present at the above levels in these vaccines through the end of 2001. (The estimates in Figure S6 do not attempt to account, due to lack of specific information, for reduced exposures that may have occurred starting in 1993 and 1994, respectively, due to use in some cases of a combined Hib/DTP vaccine with half the Hg of the separate shots (*Verstraeten et al.*, 2003) or of an Hg-free variant of the Hib vaccine (*Schechter and Grether*, 2008).) After a brief period of no thimerosal exposure, an annual flu shot was added to the recommended schedule for babies 6 months and older beginning in July, 2004. While some mercury-free flu vaccines are available, most contain 25 mcg of Hg as thimerosal. An additional 25 mcg Hg exposure was therefore assumed at each of 6, 18, 30 and 42 months.

Since many vaccines contain aluminum adjuvants, which have come under recent scrutiny as a contributor to autism (*Tomljenovic and Shaw*, 2011; *Seneff et al.,* 2012), the same vaccine schedule reconstructions described above were used to estimate the cumulative vaccine Al administered to children at each of the milestone ages listed above. Results at 18 months age are shown in Figure 5 and at a range of other ages in Figure S7a. The following vaccines can contain Al adjuvants: DPT, DTaP, Hib, HepA, HepB, PCV and the pentavalent DTaP/HepB/IPV combination. Al contents for the first 6 vaccines were taken from Table 5 of Baylor et al., 2002. The use of the pentavalent combination vaccine was assume to start in 2003 and its Al content was taken from Tomljenovic and Shaw (2011). Since the Al content (and form) in the above vaccines can vary substantially by manufacturer, the mean Al content was assumed in the calculations shown in Figure 5 and Figure S7a, while the full range of Al content was considered in Figure S7b. For example, a mean value of 385 mcg Al was assumed for DPT, although different DPT vaccines may contain 170 or 600 mcg Al (*Baylor et al.*, 2002).

Finally, a quantity defined as cumulative diseases*doses was calculated (Supplementary Figure S8). This quantity was based on the fact that children receive multiple doses of many vaccines and some vaccines (DPT, DTaP, MMR) target more than 1 disease. The disease*dose calculation was prompted by the concession issued by the U.S. Vaccine Injury Compensation Program in the Hannah Poling case (Poling et al., 2006), which suggested a connection to the sheer number of vaccines (5 shots against 9 diseases) administered to the toddler in one day rather than specifically implicating Hg or Al. The VICP conceded that her seizure disorder and autism were caused by “underlying mitochondrial dysfunction, exacerbated by vaccine-induced fever and immune stimulation” (Olmsted and Blaxill, 2010). For the disease*dose calculation, DPT, DTaP and MMR were counted as 3 diseases each and all other vaccines (including PCV7 and PCV13) as a single disease.

**S2.2.5 Atmospheric Mercury**

Despite its very low concentrations (~2 ng Hg/m3) in air, several studies have reported an association between atmospheric Hg and autism prevalence (Windham et al., 2006; Palmer et al. 2009). Two separate time series relevant to atmospheric mercury concentration and deposition were therefore examined. The first was the regionally-averaged 2002-2008 record of atmospheric wet deposition in mcg Hg/m^2^/year measured over the U.S. Great Lakes by the National Atmospheric Deposition Program (NADP) (Risch et al., 2012). The Great Lakes record was supplemented with wet deposition data for 1996-2002 from the northeastern U.S. (Vanarsdale et al., 2005). The latter are records from individual observing sites and thus display high interannual variability compared to the Great Lakes regionally-averaged data (Figure S9a). The second time series was the 1996-2010 long term record of total gaseous mercury (TGM) in ng/m^3^ measured at Mace Head, a remote site on western Ireland considered broadly representative of the Northern Hemisphere mean value (Slemr et al. 2011) (Figure S9b).

**S2.3 Polychlorinated Biphenyls (PCBs) and Dioxins**

PCBs were manufactured starting in 1929 for use in power transformers, capacitors and a host of other industrial applications. Production of PCBs was curtailed in the U.S. in 1972 and banned entirely by 1977 (Spiro and Stigliani 2003). PCBs have low flammability and high chemical stability. This latter property made them both useful in industry and persistent in the environment, where they tend to bioaccumulate in fatty tissue of animals at the top of the food chain such as large fish. Mean concentrations (ng/g wet weight) of PCBs in fish composites (lake trout and walleye) from multiple sites in the 5 North American Great Lakes were obtained from the Great Lakes Fish Monitoring Program (Carlson et al. 2012). Figure S10 shows the downward trend in data from Lakes Michigan and Ontario, which had the longest time series (1972-2002) and/or highest levels of PCBs.

The time trend (Figure S11) in the related compound 2,3,7,8-tetrachlorodibenzo-p-dioxin (TCDD), measured directly in human blood serum and adipose tissue (in ppt), as compiled by Aylward and Hayes (2002). Here, only U.S. data with relatively large (N≥25) sample size from their Table 1 were used. TCDDs are more acutely toxic than PCBs and are produced as an unintended byproduct of PCB manufacture. Other TCDD sources include the manufacture of the herbicide Agent Orange, the combustion of chlorine-containing compounds, and the bleaching of paper pulp.

**S2.4 Organophosphate Pesticides**

Organophosphate pesticides share the same chemical structure as deadly nerve gases like sarin, first developed during World War II. Unlike the organochlorine pesticides discussed below, organophosphates are not environmentally persistent, although they are generally more acutely toxic than organochlorines. U.S. annual agricultural pesticide consumption data in million lbs/year, partitioned into total insecticides, total herbicides, etc., were obtained from EPA databases for 1980-2007 (Grube et al., 2011) (Supplementary Figures S12b, S12c). These were supplemented with USDA survey data on insecticide use dating back to 1964 for 5 major crops (corn, cotton, wheat, soybean and potatotes) plus various additional fruits and vegetables (Table 4.3.1, Osteen and Livingston, 2006). The total insecticide use data were multiplied by % share of organosphosphate insecticides (compiled for the 5 major crops only) from Table 4.3.2 (Osteen and Livingston, 2006) to roughly estimate the time trend in U.S. agricultural organophosphate consumption (Supplementary Figure S12a).

Glyphosate is the active ingredient in the herbicide Roundup®. It has the basic chemical structure of an organophosphate pesticide, but is not a cholinesterase inhibitor. Rather, its mechanism of toxicity is the disruption of the shikimate pathway involved in the synthesis of the essential aromatic amino acids, phenylalanine, tyrosine, and tryptophan, in plants, a pathway that is not important in humans and other mammals, but is relevant to human gut bacteria (Samsel and Seneff, 2013). Annual estimates of glyphosate consumption were obtained from Nancy Swanson (personal communication), who combined data on crop acreage and percentage of corn and soybean crops that are genetically engineered (USDA, 2012) with data on glyphosate application rates per acre (USDA, 2010) to estimate the total amount of glyphosate applied to U.S. corn and soybean crops from 1990-2010 (Figure 6). The USDA data are state-specific and cover only the main corn and soy producing states, typically accounting for around 90% of total production, so these calculations likely slightly underestimate the total amount of glyphosate applied to corn and soy, and further neglect additional application to the other genetically engineered crops, including cotton, canola, sugar beets and alfalfa. (Note: annual reports dating back to 1990 were used in the calculations. The USDA datasets referenced are only examples of the most recent year’s report.)

**S2.5 Organochlorine Pesticides**

Organochlorine pesticides are characterized by their low solubility and low volatility and consequent environmental persistence. Like PCBs, they tend to bioaccumulate in the fatty tissue of animals at the top of the food chain. DDT, the best known organochlorine pesticide, was introduced to control malaria during World War II and, after the war, became the first widely used agricultural pesticide. As evolving insect resistance weakened DDT’s efficacy, new types of organochlorines were developed to supplement and replace it. However, the use of organochlorines as a whole was severely restricted by many countries, including the U.S., by the 1970s. The restrictions were spurred in large part by the publication by Rachel Carson in 1962 of *Silent Spring*, which warned of the ecosystem and human health threats posed by persistent organic pesticides. DDT itself was banned by the US EPA in 1972 (Spiro and Stigliani 2003).

The time trend in the concentration of DDT in North American human breastmilk fat in ng/g was estimated based on the spline fit through data from a range of Canadian and U.S. studies analyzed by Smith (1999), who reported dramatic decreases in the DDT content of human breastmilk from the 1950s to the late 1990s (Figure S13). In addition, Carlson et al. (2012) measured mean levels of a wide variety of organochlorine pesticides, including DDT, Dieldrin, Oxychlordane, Trans Nonachlor and Toxaphene in Great Lakes trout and found general declines from the mid 1980s to 2002.

**S2.6 Endocrine Disruptors**

**S2.6.1 Phthalates**

Phthalates are a family of chemicals that came into large-scale use in the 1920s as plasticizers in polyvinyl chloride (PVC) plastics. Phthalates are also used in food packaging, intravenous tubing, children's toys and building materials, including flooring, roofing, wall coverings and paints. Low molecular weight phthalates are common constituents of personal care products such as cosmetics, shampoos, nail polish, and fragrances, and in the coating of certain medications. Phthalates are suspected endocrine disruptors that adversely affect thyroid hormone function, hormone-sensitive periods of neural development and male reproductive tract development (Wittassek et al. 2007; Miodovnik et al. 2011).

Phthalates are rapidly metabolized in the human body and excreted in urine, where they can be detected via their primary or secondary metabolites. Time trends were obtained of 10 different phthalate metabolites in μg/L as detected in 24-hour urine samples collected between 1988-2003 from young adults living in Germany (Wittassek et al. 2007). Of these, Figure S14 shows results for 4 of the most abundant, including the MnBP, MiBP, MBzP, and 5OH-MEHP, which are secondary metabolites of the phthalates DnBP, DiBP, BBzP and DEHP, respectively. In their Table 5, Wittassek *et al.* compared the German data to a limited number of U.S. NHANES data for 3 data points spanning 1991-2002, which are also shown in Figure S14. Additional data compiled by (USEPA 2013, Tables B9-B10) show flat to mixed trends in urinary phthalate metabolites among U.S. women and children age 6-17 from 1998 to 2008, similar to those indicated in Figure S14.

**S2.6.2 Bisphenol A (BPA)**

Bisphenol A (BPA) is widely used in the manufacture of plastic consumer products such as water containers and bottles and is also found in the resin linings of canned foods and dental sealants. It leaches readily from many of these products and has been widely detected in human urine samples. Like phthalates, BPA has a relatively short half life in the human body (about 6 hours) (Volkel et al. 2002), such that detection in urine implies ongoing exposure. Time trends were obtained of BPA in μg/L in urine samples collected between 1995-2009 from young adults living in Germany (Kolossa-Gehring et al. 2012). This study noted some inconsistencies between German BPA production records, which showed a continuous increase to 2006, and urine levels, which began a slow decrease in 1996. The German data were compared to some recent U.S. NHANES data spanning 2003-2010 (Figure S15), which also suggest a decrease in urinary BPA in U.S women and children (USEPA 2013).

**S2.7 Automotive Exhaust and Air Pollution**

Emissions of CO, NOx, VOCs and SO2, all of which are precursors of PM2.5 in the atmosphere, were obtained from 1970-2012 from USEPA, 2012 (Figure 4). The EPA report also estimated direct PM2.5 emissions and partitioned all species into emissions from various sectors, including highway vehicles. In addition to the EPA data, time trends in both U.S. total vehicle miles and large diesel combination truck miles traveled from 1970-2010 were obtained from U.S. Dept. of Transportation data (Davis et al. 2010). To convert total miles into pollution emissions, average fleet emission rates (in kg/mile or kg/km) are needed. Since polycyclic aromatic hydrocarbons (PAHs) are the only automotive pollutant specifically named as a suspected cause of autism by Landrigan et al. (2012), emission factors for the PAH Benzene α Pyrene derived from traffic tunnel measurements (Beyea et al., 2008) were convolved with the vehicle miles traveled data (Figure S16a).

To examine the temporal trends in air pollution from direct atmospheric monitoring, USEPA surface ozone and PM2.5 data were obtained from the USEPA’s Air Quality System (AQS) database, available for download at (<http://epa.gov/ttn/airs/airsaqs/detaildata/downloadaqsdata.htm>, John Wong, personal communication). The ozone data reflect the percentage of time between April and September that each of 11 U.S. cities spanning a wide geographical range was in violation of the EPA 8-hour 75 ppb ozone standard each year from 1995-2010 (Figure S17). The PM2.5 data reflect the average annual and winter (DJF) average values (in μg/m^3^) from 5 or more monitoring sites within 0.2 km of each of Los Angeles, CA, Salt Lake City, UT, Minneapolis, MN and Newark, NJ (Figure S18). The PM2.5 data are available from 2000 onward, overlapping with the autism prevalence data for a short period from 2000-2005. The ozone violation and PM2.5 data were compared to IDEA 5 year-old prevalence data, computed as described for California, from the U.S. state in which the city resides.

**S2.8 Polycyclic Aromatic Hydrocarbons (PAHs)**

PAHs are incomplete combustion products of fossil fuels, wood, and tobacco. They can be found in both gaseous and particulate forms and are important components of fine particulate matter (PM2.5). Major sources include primary vehicular emissions, indoor heating, power plants and wood smoke. A recent comprehensive review by Shen et al. (2013) found that PAH emissions from developed countries like the U.S. peaked in the early 1970s and had declined nearly 70% by 2008. Benzene α Pyrene (BaP) is considered one of the most carcinogenic of the PAHs, although it is generally not the most abundant PAH in vehicular emissions and ambient air (Beyea et al., 2008; Chuang et al. 1999). A time series was obtained of mean U.S. fleet BaP emission factors (in mcg/km) inferred from vehicular traffic tunnel measurements between 1961 and 2004. By fitting a 4^th^ order polynomial to the 1961-1989 data and assuming for 1989 and beyond a ~constant emission factor, i.e., the mean of the 1989-2004 data excluding the anomalously high 1999 point, a continuous estimate was derived of the BaP emission factor from 1961-2004. Multiplying by total vehicular miles traveled from section S2.7, and converting from miles to km, yielded total U.S. vehicular BaP emissions from 1961-2005 (Figure S16a). A shorter but more comprehensive time series of total PAH exposure was obtained from a New York City study in which pregnant women’s exposure was tracked with ambient mobile personal monitoring devices (Narvaez et al., 2008) between 1998 and 2006 (Figure S16b).

**S2.9 Polybrominated Diphenyl Ethers (PBDEs)**

Polybrominated diphenyl ethers (PBDEs) are widely used as flame retardant additives in consumer products including styrofoam, electronic equipment, building materials, textiles and carpet linings. Although their benzene rings are substituted with bromine rather than chlorine atoms, PBDEs are similar both chemically and toxicologically to the PCBs discussed above, with 209 different possible congeners (i.e., possible arrangements of Br or Cl substituents). Like PCBs, PBDEs are persistent organic chemicals that can bioaccumulate in fatty tissue. A key difference is that while PCBs were largely phased out in the late 1970s, PBDE manufacture and use has grown rapidly over the past few decades. However, concerns over the association of PBDEs with endocrine disruption, particularly with respect to thyroid hormones, as well as reproductive and developmental toxicity has led to restrictions on their use and manufacture, with many commercial BDE mixtures banned by the mid 2000s in the U.S. (Schechter et al. 2005; Herbstman et al. 2009).

The literature survey conducted here was unable to find a time series of PBDEs measured directly in American bodily fluids or tissue. However, a long-term time record was found of total PBDEs (sum of 47, 99, 100, 153) measured in Great Lakes trout in ng/g (Batterman et al. 2007). Data were presented for all 5 Lakes (Figure S19). Table S1 reports the correlation coefficients between AD and the 1979-2000 record from Lake Michigan, where trout have higher PBDE concentrations than the other lakes, and the 1984-2005 record from Lake Ontario.

**S2.10 Perfluorinated Compounds (PFCs)**

Perfluorinated compounds (PFCs), including Perfluorooctanoic acid (PFOA) and Perfluorooctane sulfonate (PFOS), are a family of persistent environmental compounds with half lives ranging from ~3-8 years in the human body. PFCs were first manufactured in the 1950s and have been used widely in nonstick cookware, coatings on paper and packaging, including in food contact papers, and as surface coatings and treatments for carpets, clothing and other fabrics. Animal toxicology studies have found that PFCs have adverse effects on fetal growth and development, while epidemiological studies have suggested a link to ADHD in children. In response to these concerns, the primary U.S. manufacturer of PFCs phased out most production in 2002 (Kato et al., 2011).

A time series extending from 1972 to 2008 of PFCs in pg/mL in human breastmilk from Sweden was obtained from Sundstrom et al. (2011). The time series covers the 3 most abundant PFCs detected in the breastmilk, including PFOS, PFOA and PFHxS (Figure S20a). A shorter time series, extending from 1999-2010, of PFCs in U.S. women’s blood (in ng/ml) was obtained from a combination of NHANES and American Red Cross data (Kato et al., 2011; Olsen et al., 20102). The U.S. data included PFNA in addition to the 3 PFCs listed above in the Swedish study (Figure S20b).

**References**

Aylward, L.L. and Hays, S.M., 2002, Temporal Trends in human TCDD body burden: Decreases over three decades and implications for exposure levels, *Journal of Exposure Analysis and Environmental Epidemiology, 12*, 319-328.

Batterman, S., Chernyak, S., Gwynn, E., Cantonwine, D., Jia, C., Begnoche, L., Hickey, J.P., 2007, Trends of brominated diphenyl ethers in fresh and archived Great Lakes fish (1979–2005), *Chemosphere, 69*, 444-457.

Baylor NW, Egan W, Richman P., 2002, Aluminum salts in vaccines--US perspective. *Vaccine* *20 Suppl 3*, S18–23.

Beyea, J., S.D. Stellan, M. Hatch and M.D. Gammon, 2008, Airborne Emissions from 1961 to 2004 of Benzo[a]pyrene from U.S. Vehicles per km of Travel Based on Tunnel Studies, *Environ. Sci. Technol*., 42, 7315-7320.

Caldwell, K.L., Mortensen, M.E., Jones, R.L., Caudill, S.P., Osterloh, J.D., 2009, Total blood mercury concentrations in the U.S. population: 1999-2006, *Int. J. Hyg. Environ. Health, 212*, 588-598.

California Health and Human Services Agency Dept of Developmental Services (CDDS): *Autistic Spectrum Disorders. Changes in California Caseload. An Update: 1999 Through 2002*. Sacramento, CA; 2003.

Carlson, D.L., De Vault, D.S. and Swackhamer, D.L., 2010, On the Rate of Decline of Persistent Organic Contaminants in Lake Trout (Salvelinus namaycush) from the Great Lakes, 1970-2003, *Environ. Sci. Technol., 44*, 2004-2010.

Centers for Disease Control and Prevention (CDC), 2014, Prevalence of autism spectrum disorder among children aged 8 years -Autism and Developmental Disabilities Monitoring Network, 11 sites, United States, 2010, *MMWR Surveill Summ 2014*, 63 Suppl2, 1–21.

Chuang, J.C., Callahan, P.J., Lyu, C.W. and Wilson, N.K., 1999, Polycyclic aromatic hydrocarbon exposures of children in low-income families, *Journal of Exposure Analysis and En*z*ironmental Epidemiology* 2, 85–98.

Davis, S.C., S.W. Diegel, and R.G. Boundy. 2010, Transportation Energy Data Book: Edition 29; ORNL-6985; Center for Transportation Analysis, Oak Ridge National Laboratory: Oak Ridge, 2010.

Dufault R, LeBlanc B, Schnoll R, Cornett C, Schweitzer L, Wallinga D, et al., 2009a: Mercury from chlor-alkali plants: measured concentrations in food product sugar. *Environ Health* 2009, 8:2.

Dufault, R., Schnoll, R., Lukiw, W.J., LeBlanc, B., Cornett, C., Patrick, L., et al., 2009b, Mercury exposure, nutritional deficiencies and metabolic disruptions may affect learning in children, *Behavioral and Brain Functions* 2009, 5:44 doi:10.1186/1744-9081-5-44.

Grube, A., Donaldson, D., Kiely, T., and Wu, L., 2011, Pesticides industry sales and usage, 2006 and 2007 market estimates, US EPA. Accessed through USDA website (<http://www.ers.usda.gov/topics/farm-practices-management/chemical-inputs/pesticide-use-markets.aspx>).

Herbstman JB, Sjödin A, Kurzon M, Lederman SA, Jones RS, Rauh V, et al. 2010. Prenatal exposure to PBDEs and neurodevelopment. *Environ Health Perspect* 118:712–719.

Jusko TA, Henderson CR Jr, Lanphear BP, Cory-Slechta DA, Parsons PJ, Canfield RL. 2008. Blood lead concentrations < 10 μg/dL and child intelligence at 6 years of age. Environ Health Perspect 116:243–248.

Kato, K., Wong, L.-Y., Jia, L.T., Kuklenyik, Z., Calafat, A.M., 2011, Trends in Exposure to Polyfluoroalkyl Chemicals in the U.S. Population: 1999-2008, *Environ. Sci. Technol., 45*, 8037–8045.

Kolossa-Gehring et al., 2012, Environmental surveys, specimen bank and health related environmental monitoring in Germany, *International Journal of Hygiene and Environmental Health, 215*, 120–126.

Laks, D.R., 2009, Assessment of chronic mercury exposure within the U.S. population, National Health and Nutrition Examination Survey, 1999–2006, *Biometals, 22*, 1103–1114.

Ljungvall, A. and F.J. Zimmerman, 2012, Bigger bodies: Long-term trends and disparities in obesity and body-mass index among U.S. adults, *Social Science & Medicine, 75*, 109-119.

Mahaffey, K.R., R.P. Clickner, and R.A. Jeffries, 2008, Methylmercury and omega-3 fatty acids: Co-occurrence of dietary sources with emphasis on fish and shellfish, Environmental Research 107, 20–29.

Mahaffey, K.R., R.P. Clickner, and R.A. Jeffries, 2009, Adult Women's Blood Mercury Concentrations Vary Regionally in the United States: Association with Patterns of Fish Consumption (NHANES 1999-2004), EHP, 117(1).

McCall, P.L. and Land, K.C., 2004, Trends in environmental lead exposure and troubled youth, 1960–1995: an age-period-cohort-characteristic analysis, *Social Sci Research, 33(2)*, 339-359.

Miodovnik, A., S.M. Engel, C. Zhu, X. Ye, L.V. Soorya, M.J. Silva, et al., 2011, Endocrine Disruptors and Childhood Social Impairment, *Neurotoxicology*, 32(2): 261–267. doi:10.1016/j.neuro.2010.12.009.

Narvaez, R.F., et al., 2008, Spatial and Temporal Trends of Polycyclic Aromatic Hydrocarbons and Other Traffic-Related Airborne Pollutants in New York City, *Environ. Sci. Technol.*, 42, 7330–7335.

Olmsted, D. and Blaxill, B, 2010, The age of autism: mercury, med[1]icine and a man-made epidemic, Thomas Dunne Books, 2010, 430 pp.

Olsen, G., et al., 2012, Temporal Trends of Perfluoroalkyl Concentrations in American Red Cross Adult Blood Donors, 2000−2010, *Environ. Sci. Technol., 46*, 6330−6338.

Osteen, C. and Livingston, M., 2006. Pest Management Practices. In: Agricultural Resources and Environmental Indicators 2006 Edition, Chapter 4.3: In (Wiebe K, Gollehon N, eds) EIB-16, USDA. Available: <http://www.ers.usda.gov/publications/eib-economic-information-bulletin/eib16.aspx>.

Palmer, R.F., S. Blanchard, and R. Wood, 2009. Proximity to point sources of environmental mercury release as a predictor of autism prevalence. Health and Place 15: 18–24.

Phillips, P.J., S.W. Ator and E.A. Nystrom, 2007, Temporal Changes in Surface-Water Insecticide Concentrations after the Phaseout of Diazinon and Chlorpyrifos, *Environ. Sci. Technol.* 41, 4246-4251.

Poling J, Frye R, Shoffner J, Zimmerman AW, 2006, Developmental regression and mitochondrial dysfunction in a child with autism. *J Child Neurol* 21: 170–172.

Risch, M.R., D.A. Gay, K.K. Fowler, G.J. Keeler, S.M. Backus, et al., 2012. Spatial patterns and temporal trends in mercury concentrations, precipitation depths, and mercury wet deposition in the North American Great Lakes region, 2002-2008, *Environmental Pollution* 161, 261-271.

Samsel, A. and S. Seneff, 2013, Glyphosate’s suppression of cytochrome P450 enzymes and amino acid biosynthesis by the gut microbiome: Pathways to modern diseases, Entropy, *15*, 1-x manuscripts; doi:10.3390/ e140x000x.

Schechter, R., and Grether, J, 2008, Continuing increases in autism reported to California’s developmental services system, Archives of General Psychiatry, 65(1), 19-24.

Schechter, A., Paepke, O., Tung, K.C., Joseph, J., Harris, R.T., Dahlgren, J., 2005, Polybrominated Diphenyl Ether Flame Retardants in the U.S. Population: Current Levels, Temporal Trends, and Comparison With Dioxins, Dibenzofurans, and Polychlorinated Biphenyls, *J. Occupational and Environ Medicine*, 47(3).

Seneff, S., R.M. Davidson, and J.Liu, 2012, Empirical Data Confirm Autism Symptoms Related to Aluminum and Acetaminophen Exposure, *Entropy*, *14*, 2227-2253; doi:10.3390/e14112227.

Shen, H., Y. Huang, R. Wang et al., 2013, Global atmospheric emissions of polycyclic aromatic hydrocarbons from 1960 to 2008 and future predictions, Environ Sci Tech, *Environ. Sci. Technol.*, Just Accepted Manuscript, doi: 10.1021/es400857z.

Silver, M.K., M.S. O’Neill, M.R. Sowers, S.K. Park, 2011, Urinary Bisphenol A and Type-2 diabetes in U.S. adults: Data from NHANES 2003-2008, PLoS one, 6(10).

Slemr, F.; Brunke, E.-G.; Ebinghaus, R.; Kuss, J., 2011, Worldwide trend of atmospheric mercury since 1995, *Atmos. Chem. Phys.* *11*, 4779– 4787.

Smith, D., 1999, Worldwide trends in DDT levels in human breast milk, *Int. J. of Epidemiology, 28*, 179-188.

Spiro, T.G. and W.M. Stigliani, Chemistry of the Environment, 2^nd^ Edition, Prentice Hall, NJ, 489pp, 2003.

Sundstrom, M., Ehresan, D.J., Bignert, A., Butenhoff, J.L., Olsen, G.W., Chang, S.-C., Bergman, A., 2011, A temporal trend study (1972–2008) of perfluorooctanesulfonate(PFOS), perfluorohexanesulfonate(PFHxS), and perfluorooctanoate(PFOA) in pooled human milk samples from Stockholm, Sweden, *Environment International, 37*, 178–183.

Tomljenovic, L. and C.A. Shaw, 2011, Do aluminum vaccine adjuvants contribute to the rising prevalence of autism?, *J. Inorganic Biochemistry*, *105*, 1489-1499.

USDA (United States Department of Agriculture/National Agricultural Statistics Service (NASS)), 2010, Agriculture Chemical Use Program, http://www.nass.usda.gov/Surveys/Guide_to_NASS_Surveys/Chemical_Use/index.asp

USDA (United States Department of Agriculture/National Agricultural Statistics Service (NASS)), 2012, Acreage, ISSN 1949-1522, http://usda01.library.cornell.edu/usda/nass/Acre//2010s/2012/Acre-06-29-2012.pdf

USEPA (United States Environmental Protection Agency): *National Emissions Inventory (NEI) Air Pollutant Emissions Trends Data*. 2012.

USEPA (United States Environmental Protection Agency), 2013, America's Children and the Environment, 3rd Edition, EPA 240-R-13-001, January 2013, 504pp.

Vanarsdale, A., J. Weiss, G. Keeler, E. Miller, G. Boulet, R. Brulotte, and L. Poissant, 2005, Patterns of mercury deposition and concentration in Northeastern North America (1996-2002), *Ecotoxicology, 14*, 37-52.

Verstraeten, T., R.L. Davis, F. DeStefano, T.A. Lieu, P.H. Rhodes, S.B. Black, et al., 2003, Safety of Thimerosal-Containing Vaccines: A Two-Phased Study of Computerized Health Maintenance Organization Databases, *Pediatrics,* 112;1039.

Volkel W, Colnot T, Csanady GA, Filser JG, Dekant W., 2002, Metabolism and kinetics of bisphenol A in humans at low doses following oral administration, *Chem Res Toxicol 15(10)*, 1281–1287.

Windham, G.C., L.X. Zhang, R. Gunier, L.A. Croen, and J.K. Grether. 2006. Autism spectrum disorders in relation to distribution of hazardous air pollutants in the San Francisco Bay area. *Environmental Health Perspectives 114*: 1438–44.

Wittassek, M., Wiesmueller, G.A., Koch, H.M, Eckard, R., Dobler, L., Mueller, J., Angerer, J., and Schlueter, C., 2007, Internal phthalate exposure over the last two decades –A retrospective human biomonitoring study, *Int. J. Hyg. Environ.-Health 210*, 319–333.

World Health Organization (WHO), 2012, Immunization Summary, available online as <http://www.childinfo.org/files/immunization_summary_en.pdf> and <http://apps.who.int/immunization_monitoring/en/globalsummary/ScheduleSelect.cfm>.


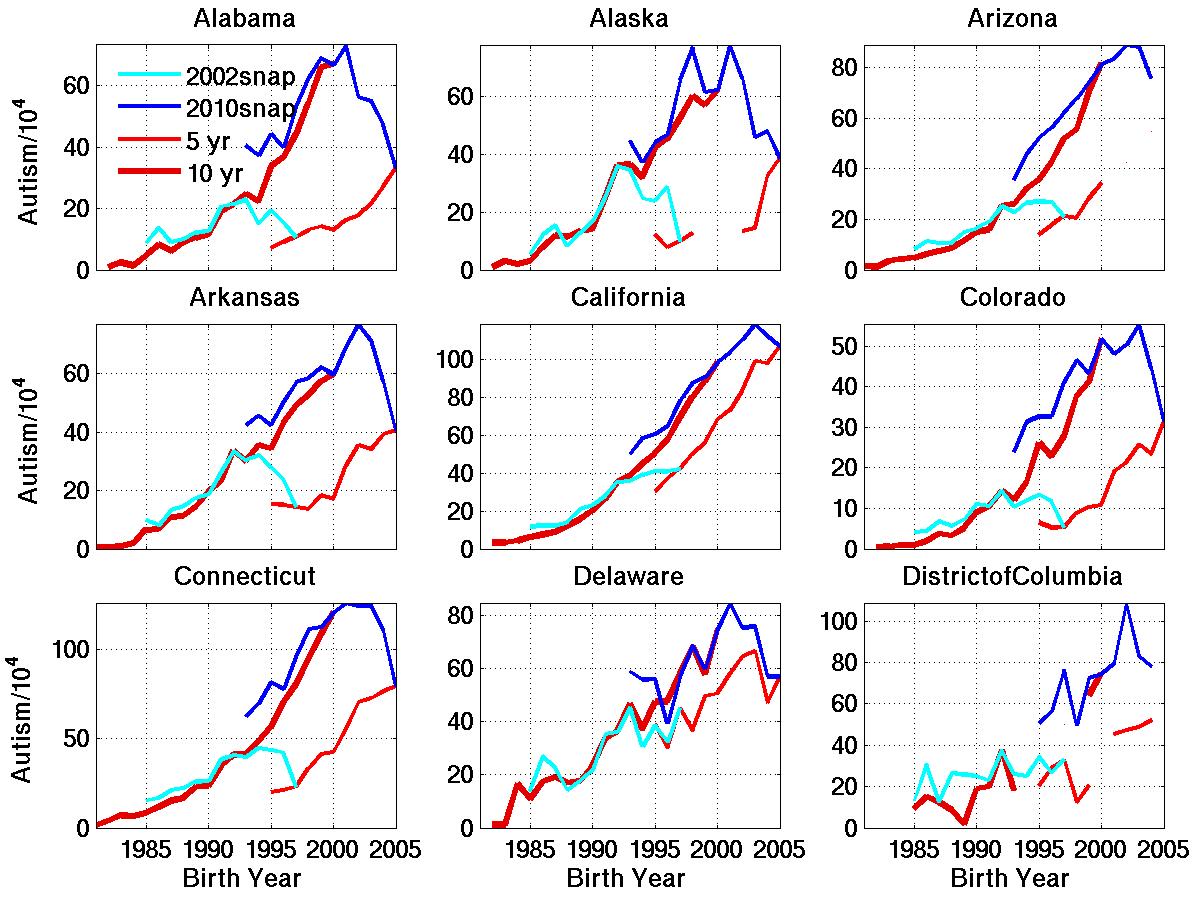

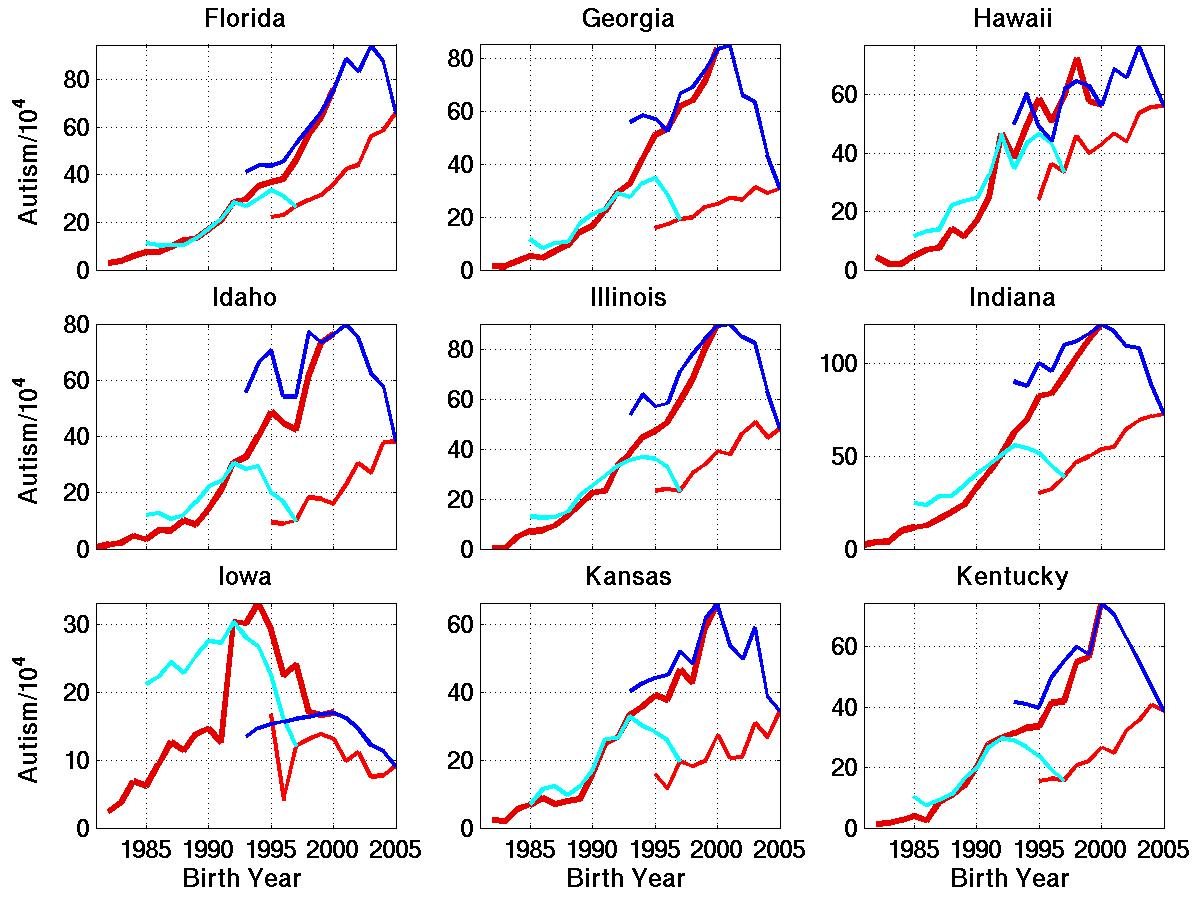

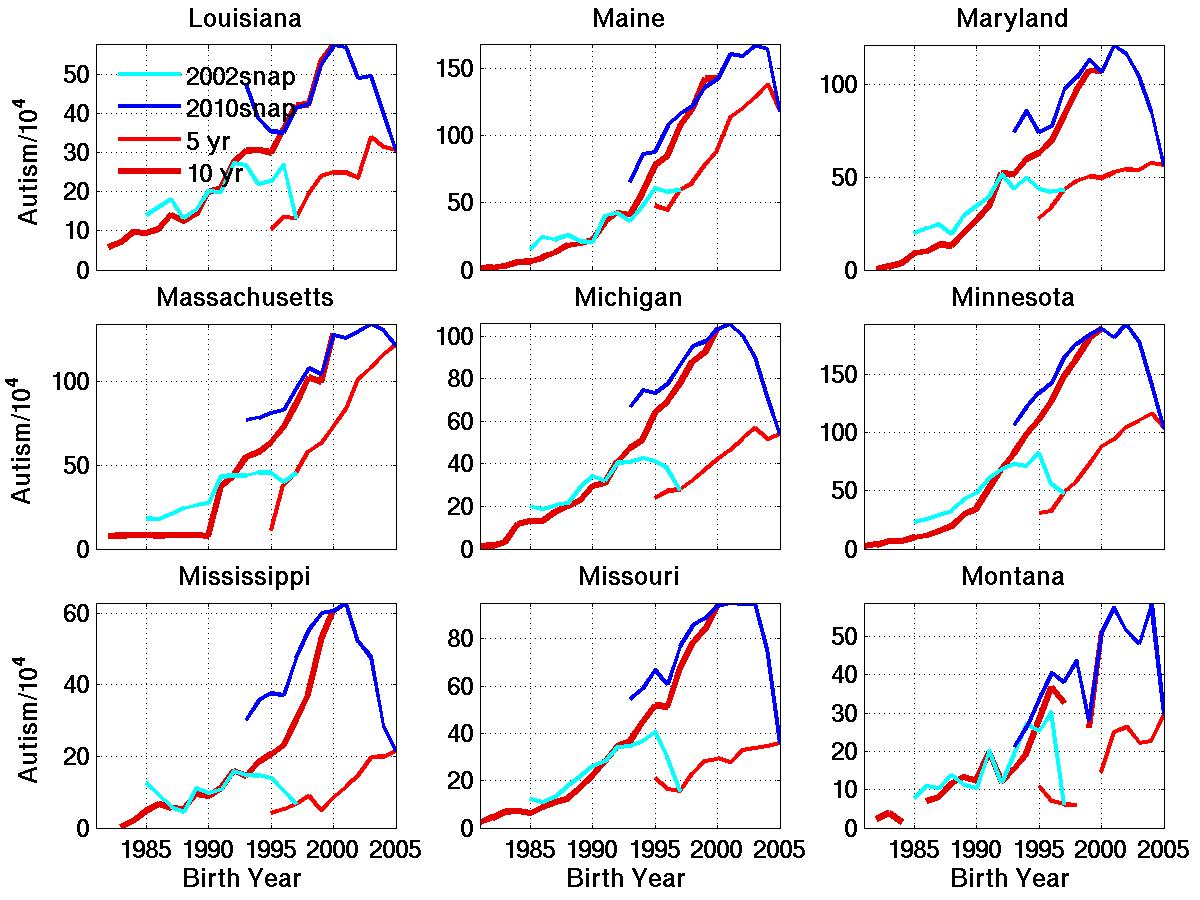

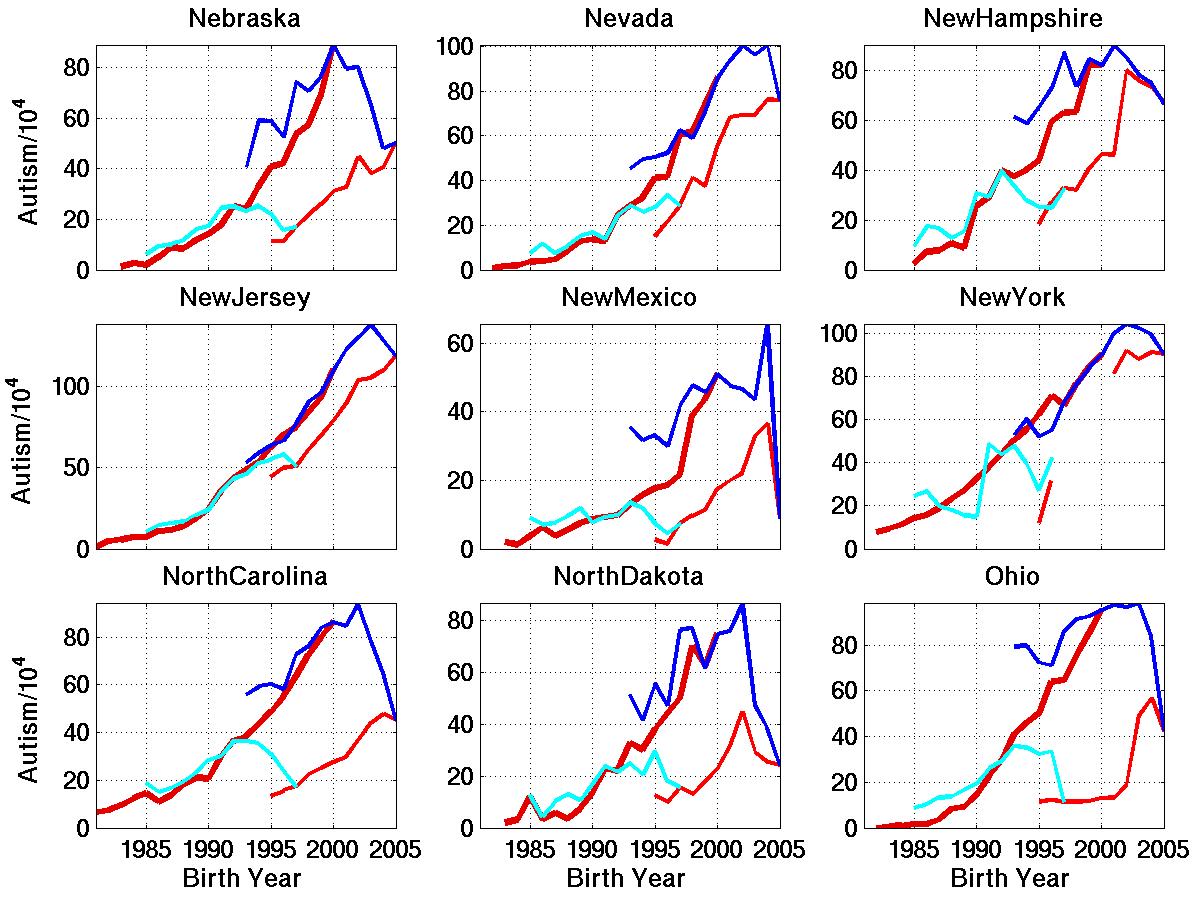

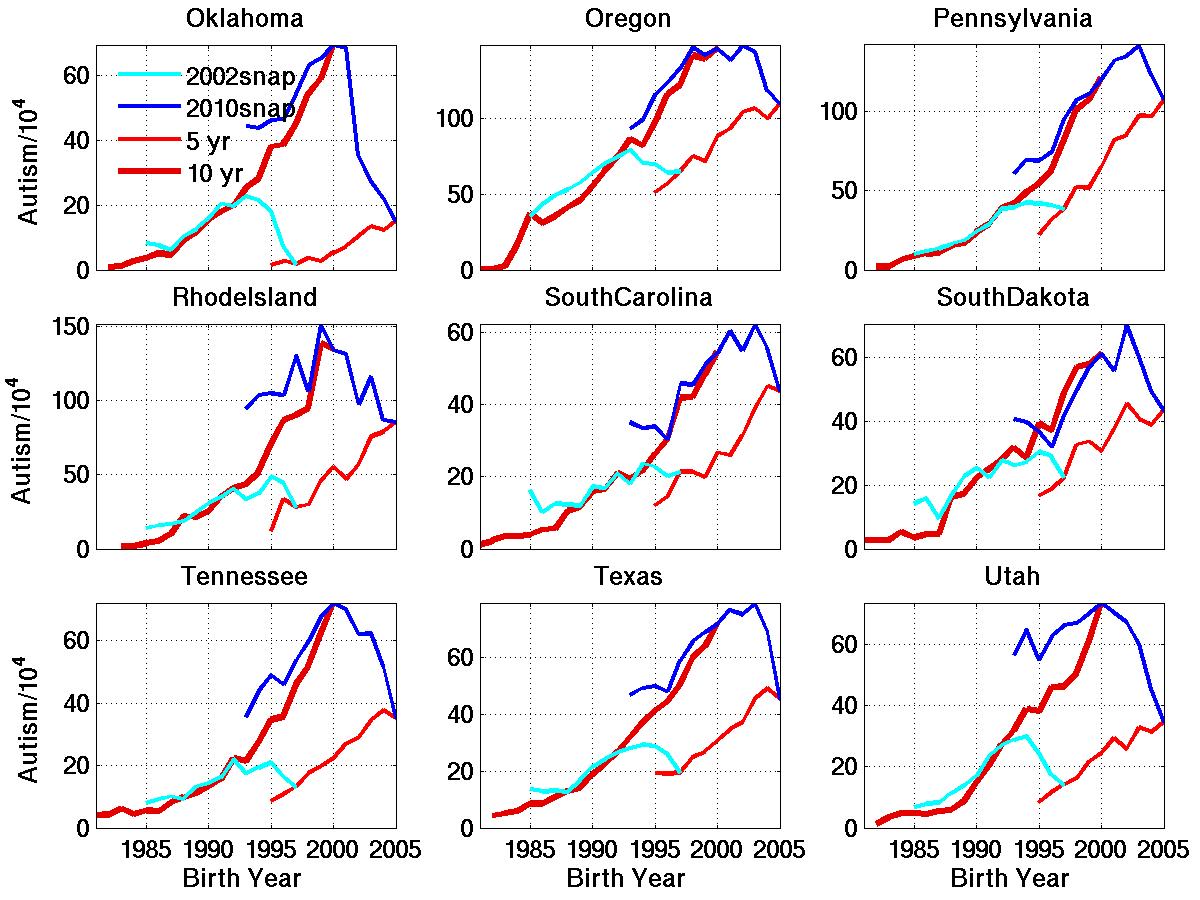


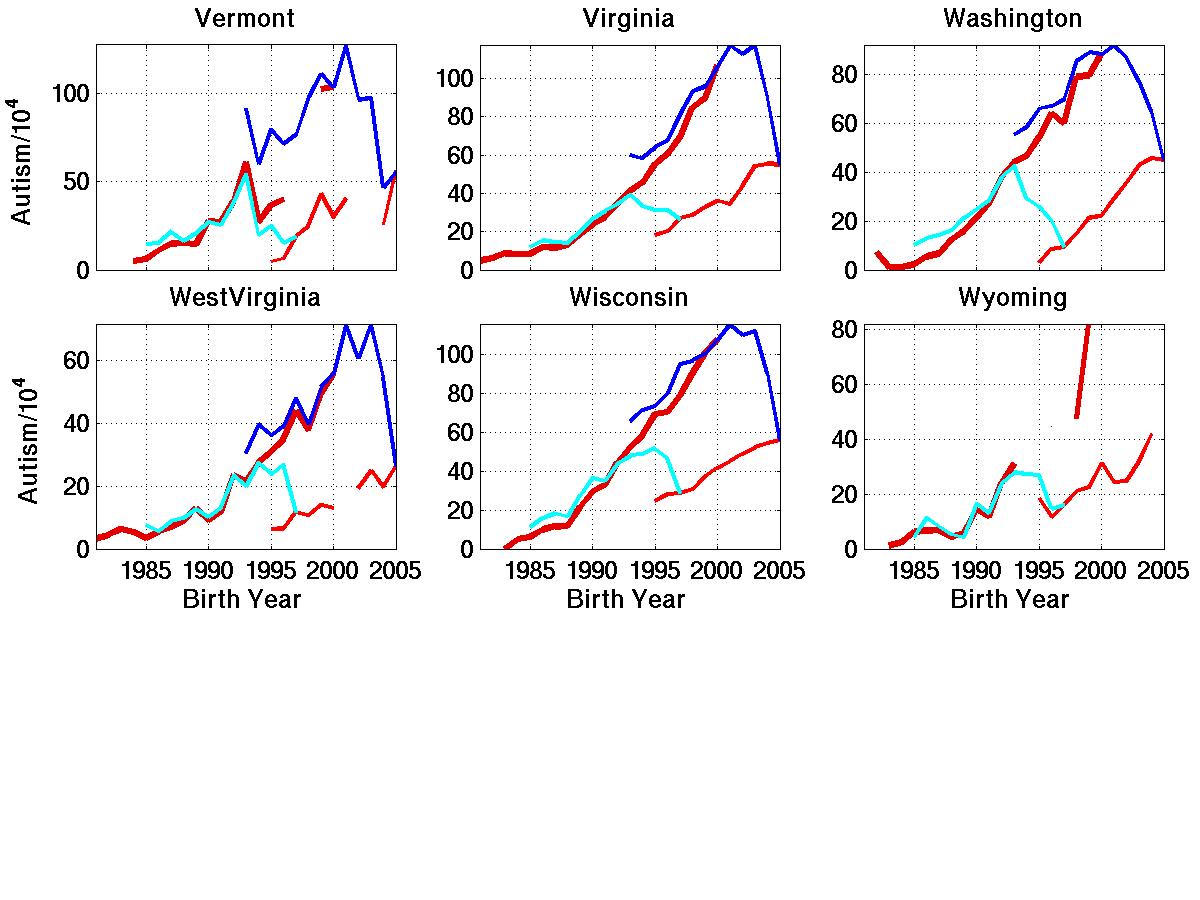


Figure S1. Temporal trends in autism in all 50 states and the District of Columbia calculated as described in Supplement S1 using IDEA data. Two different methods for estimating the trend are compared: Tracking of specific ages (5 and 10) over many years of IDEA reports vs. age-resolved snapshots from an individual year’s report (snapshots shown from the 2002 and 2010 reports).


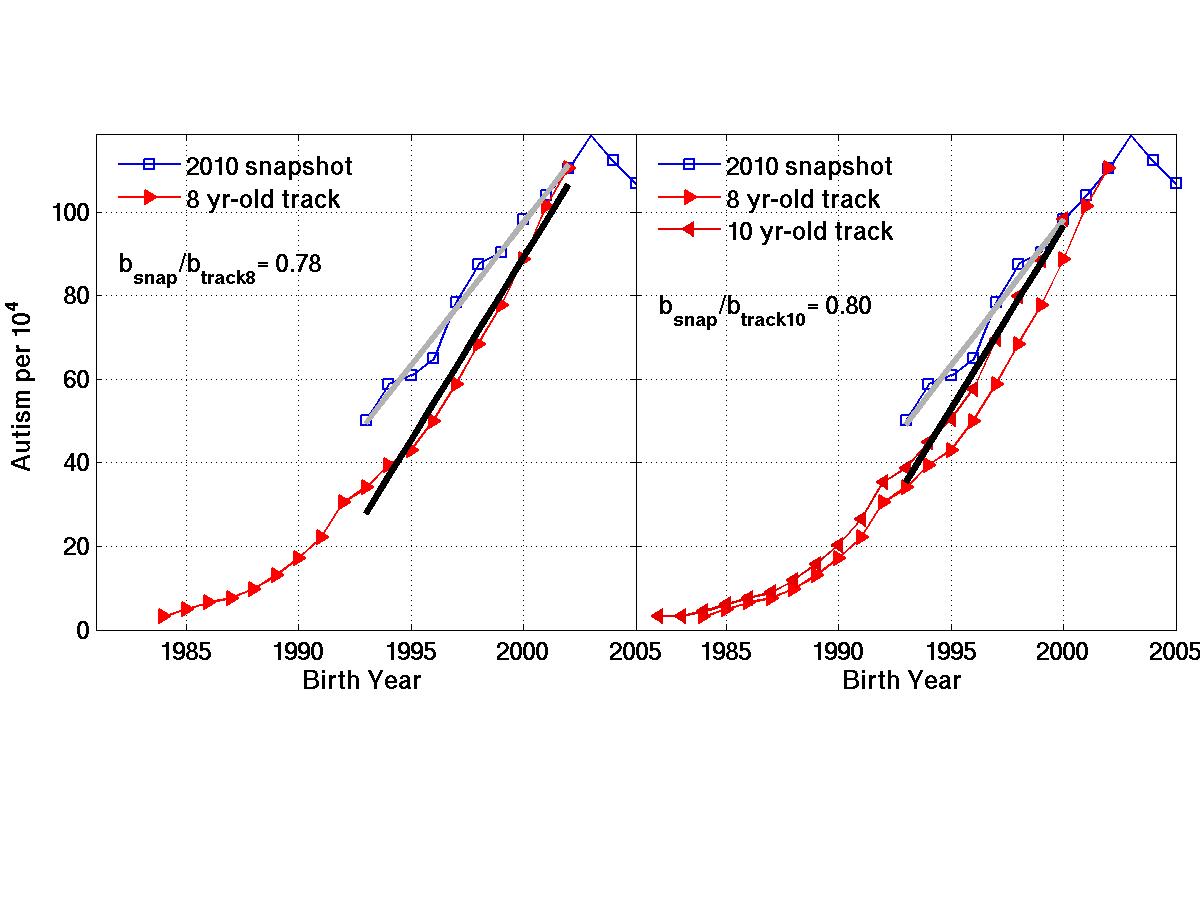
Figure S2a. Autism prevalence vs. birth year plots based on California IDEA data, as described in Figure 1, derived independently using age-resolved snapshot and constant-age tracking methods. The two panels illustrate the relatively small sensitivity of the trend lines to the choice of the constant tracking age and the snapshot start age (which are fixed at the same value to achieve exactly overlapping birth year intervals). Left panel uses age 8, fitting linear trends over birth year interval 1993-2002. Right panel uses age 10, fitting linear trends over birth year interval 1993-2000. Right panel also illustrates the difference between the 8 and 10 year-old constant-age tracking curves. Symbol b refers to slope of the linear fits.


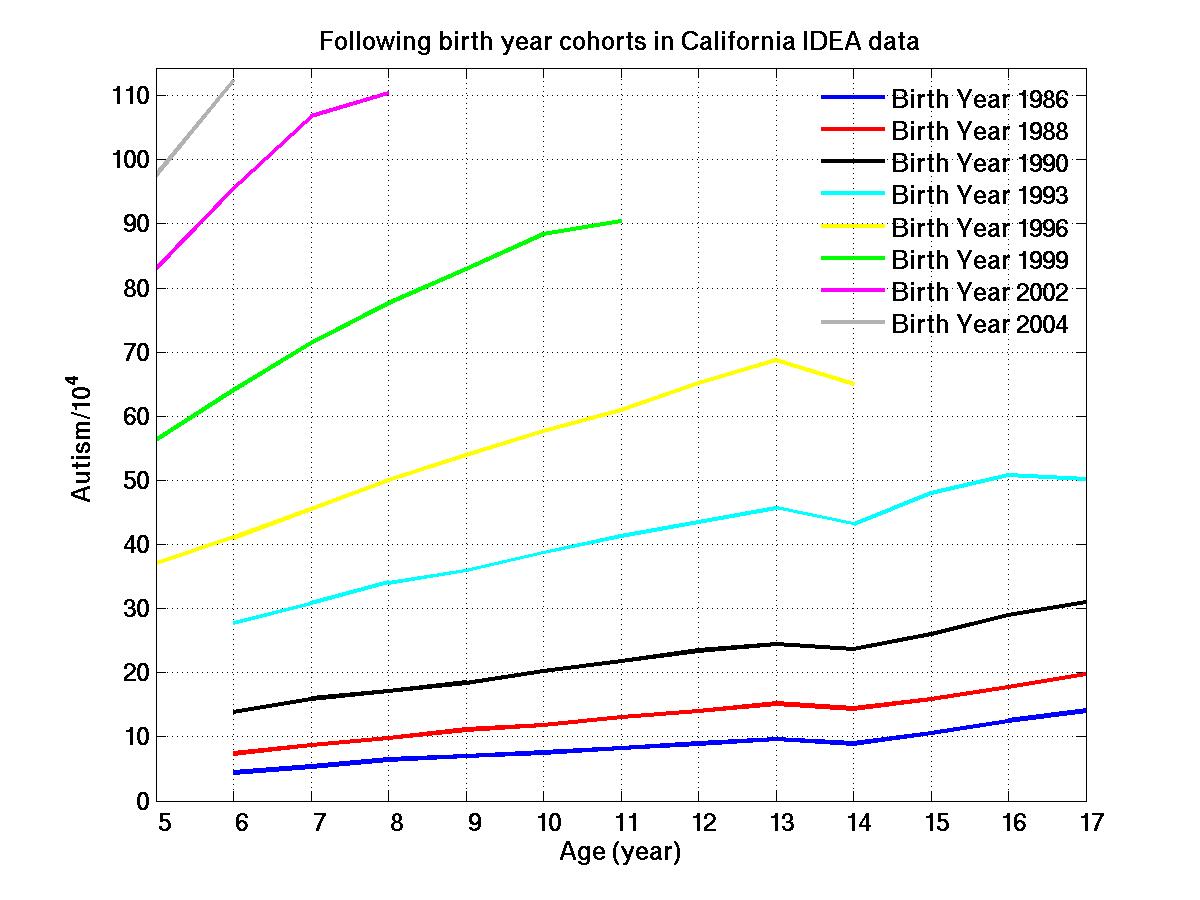


Figure S2b. Autism prevalence for California IDEA/NCES data, using annual reports from 1991-2010, following 8 different birth year cohorts as they age.
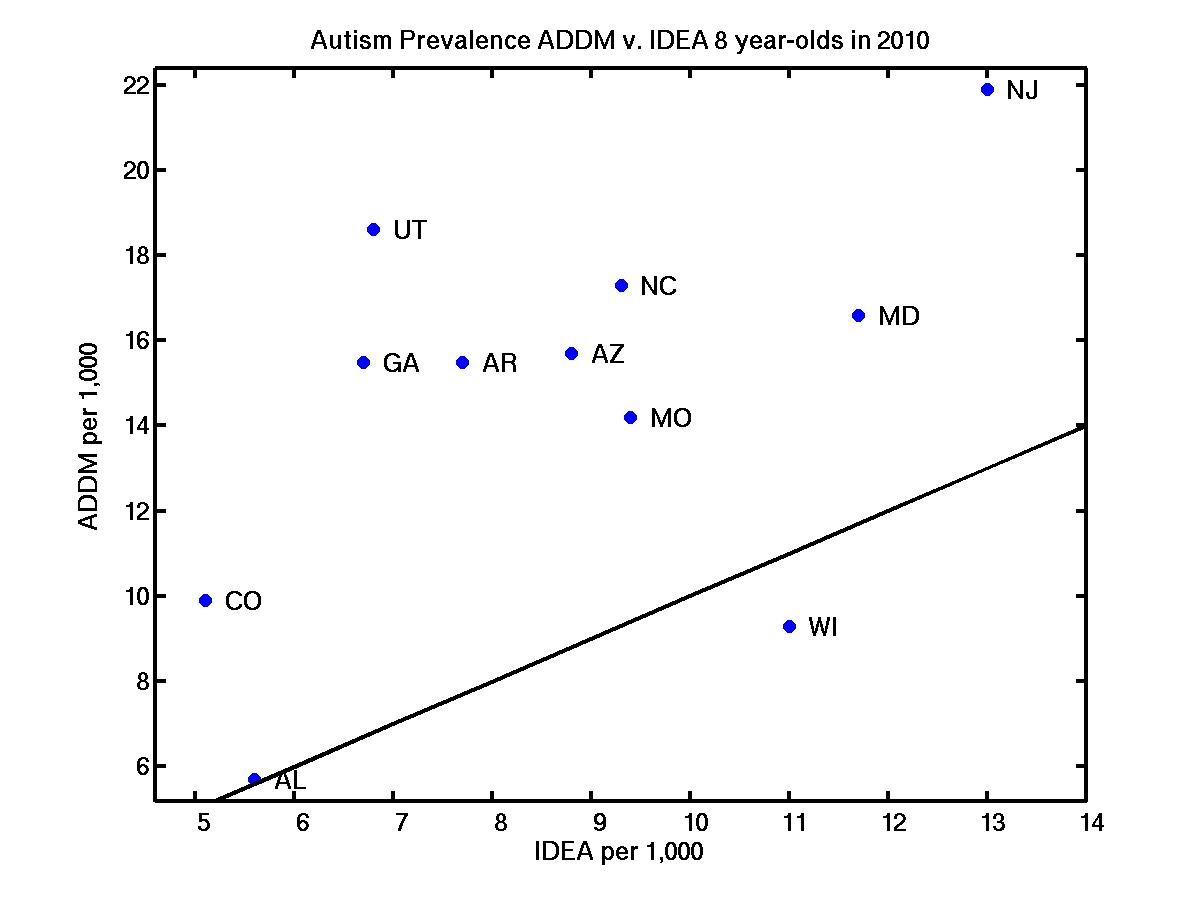
Figure S2c. Autism prevalence reported in 2010 among 8 year-olds (birth year 2002) from IDEA/NCES, calculated as described above, compared to ASD prevalence in the corresponding 11 available states from the ADDM Network (CDC, 2014). Solid black curve is 1:1 line.


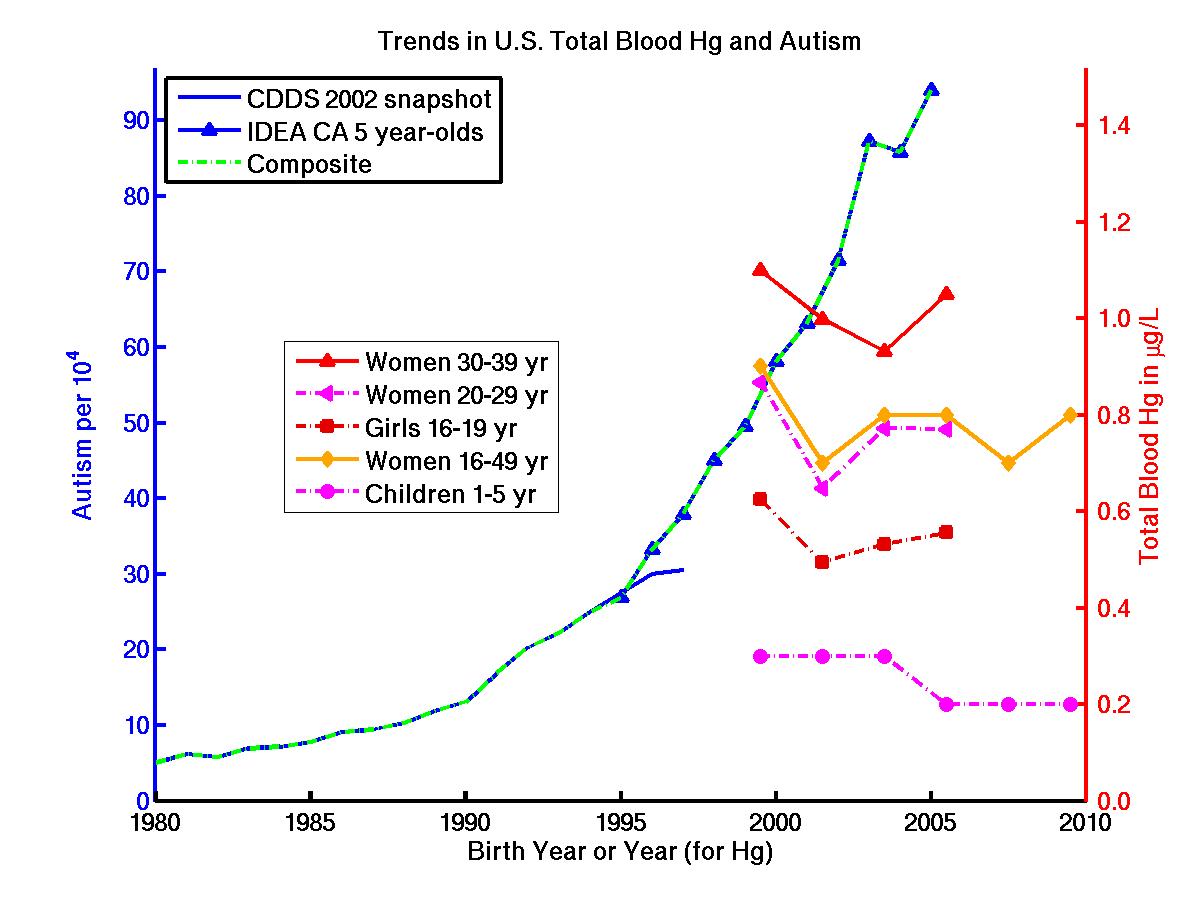


Figure S3. Temporal trend in autism compared to temporal trends in total blood mercury in U.S. women of childbearing age and young children (*Caldwell et al.*, 2009; *USEPA*, 2013). While there is no obvious trend toward increasing blood Hg with time, the data suggest a trend toward increasing blood Hg with age.


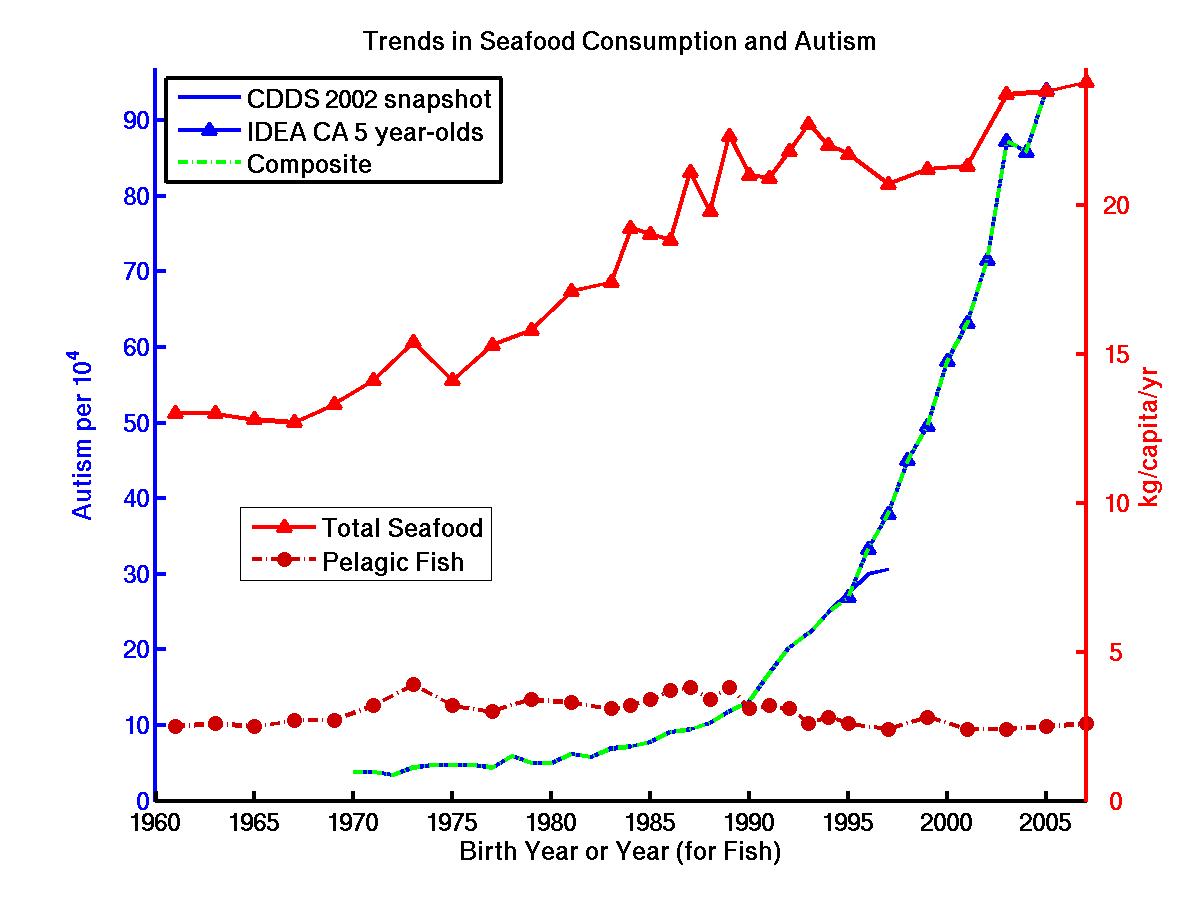


Figure S4a. Temporal trend in autism compared to temporal trends in U.S. per capita total seafood consumption and pelagic fish consumption. Data are from FAOSTAT food balance sheets.


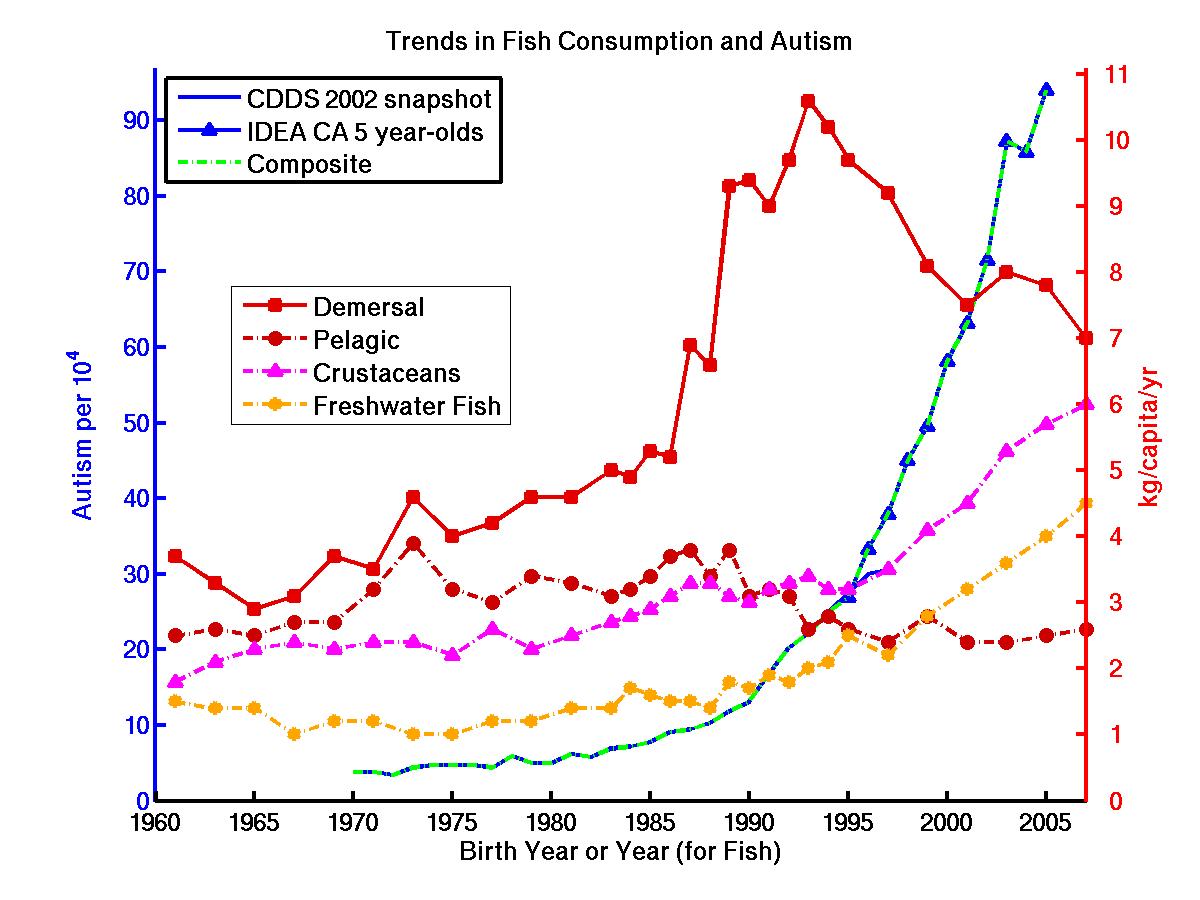


Figure S4b. Temporal trend in autism compared to temporal trends in U.S. per capita fish and seafood consumption, partitioned by type of fish or seafood. Data are from FAOSTAT food balance sheets.


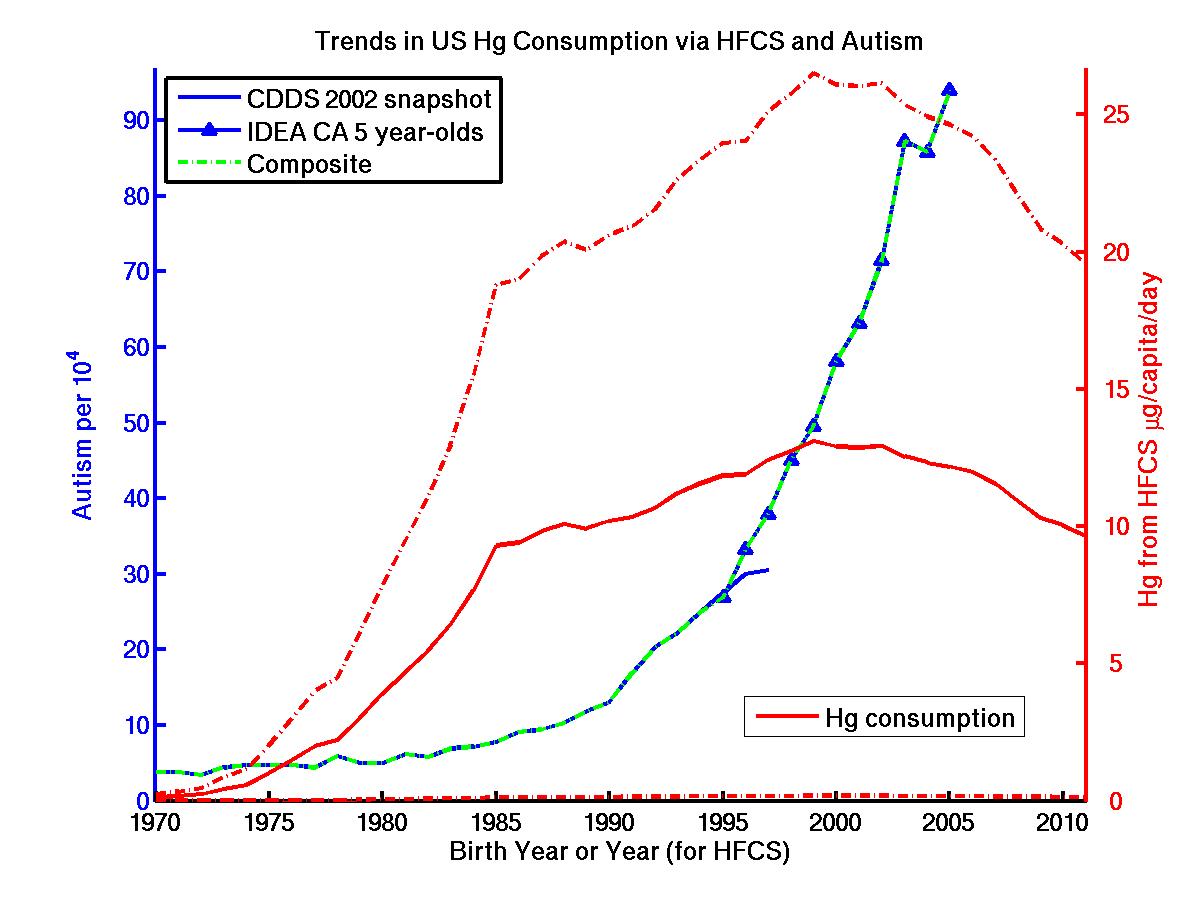


Figure S5. Temporal trend in autism compared to temporal trend in U.S. per capita Hg consumption via high fructose corn syrup (HFCS). Data are based on HFCS consumption data from the *USDA* (2013) multiplied by the mean Hg content (i.e., mean of the 9 detectible samples out of 20 total samples) per g HFCS as reported by *Dufault et al.*, 2009a. The wide range of uncertainty in the minimum and maximum exposure (dashed red lines) reflects the fact that only HFCS produced with caustic soda from mercury cell chlor-alkali plants is contaminated with Hg.


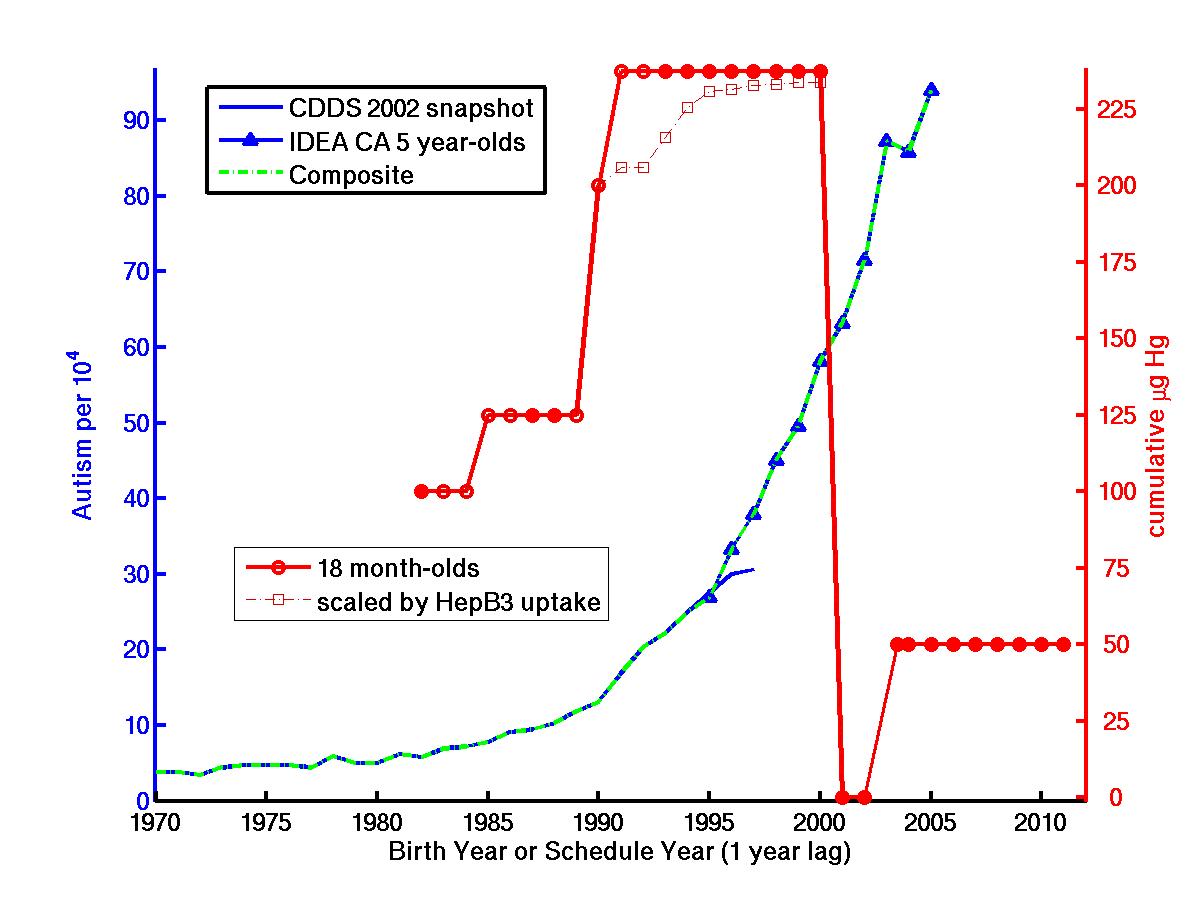


Figure S6a. Temporal trend in autism compared to temporal trend in cumulative amount of Hg as thimerosal administered post-natally to U.S. toddlers by age 18 months via immunization according to the CDC recommended schedule. The immunization curve is lagged 1 year because 18 month-olds born, e.g., in 1994 will likely follow the 1995 schedule. Large, solid red circles are years with published CDC schedules. Open red circles reflect educated guesses (see Supplementary Information for details) in gap years without published schedules. Open boxes reflect the estimated effect of the gradual uptake of the HepB vaccine, based on yearly uptake of the 3^rd^ HepB shot given in *WHO* (2012). (Uptake of the other thimerosal-containing vaccines is > 95% for all years.)


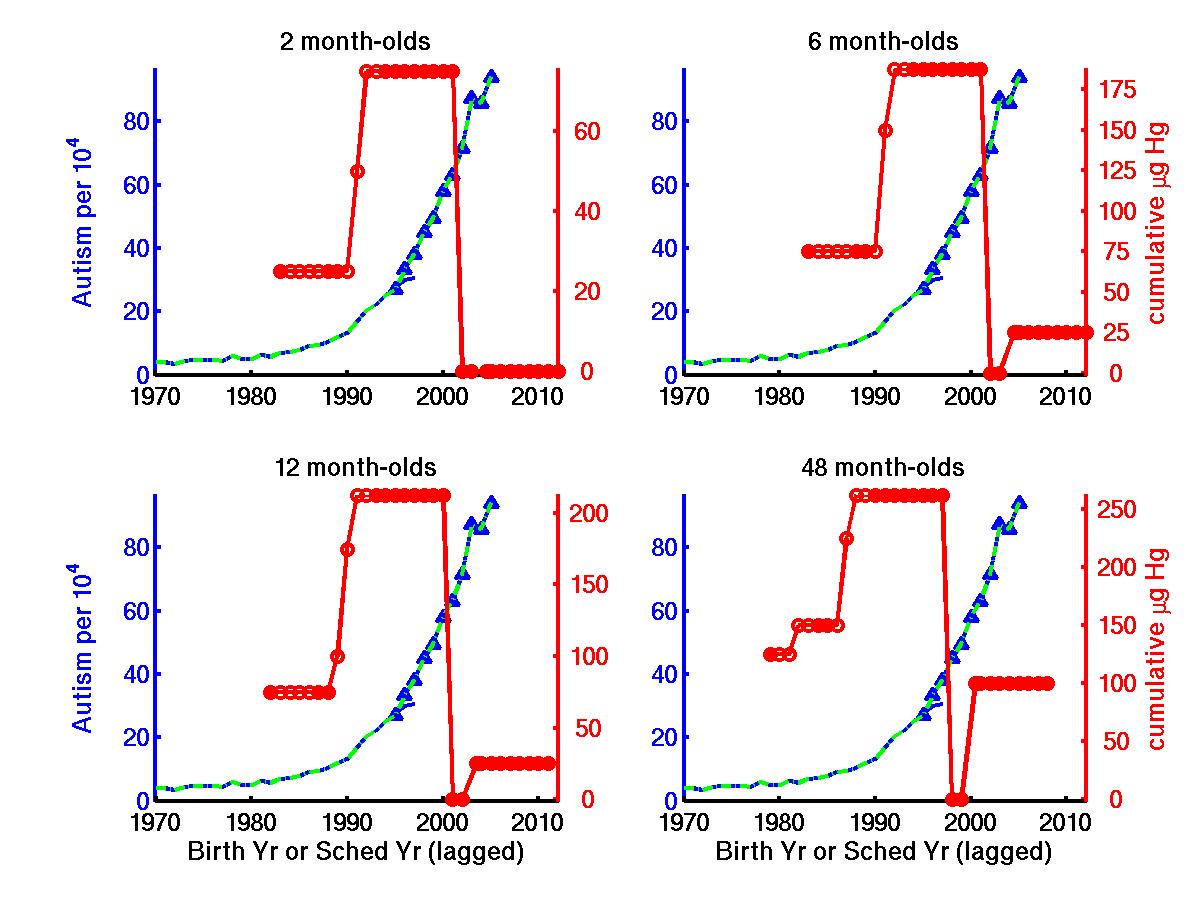


Figure S6b. Temporal trend in autism compared to temporal trend in cumulative amount of Hg as thimerosal administered post-natally to U.S. infants and toddlers by 2,6,12 and 48 months via immunization according to the CDC recommended schedule. The thimerosal curves are lagged by milestone age rounded down to nearest year (i.e., 0,0,1, and 4 years, respectively).


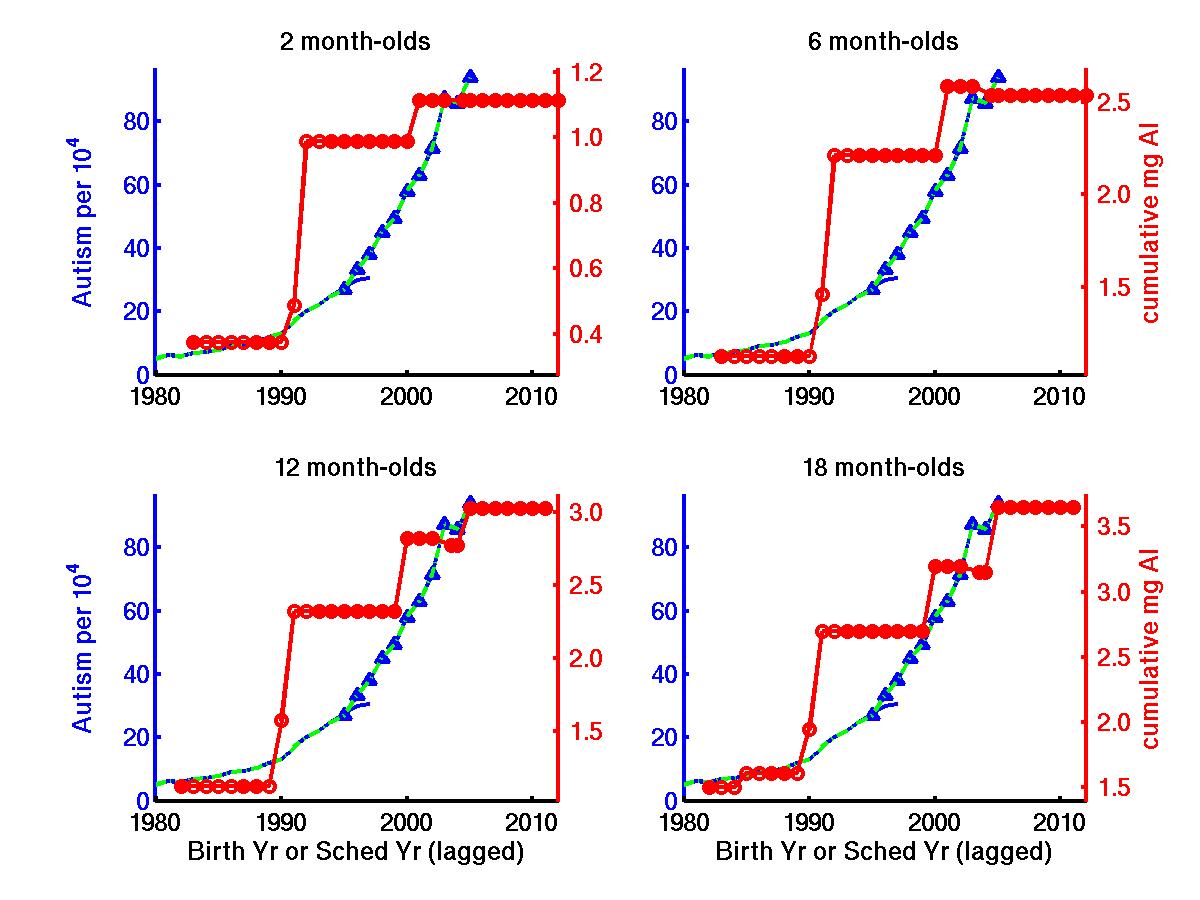


Figure S7a. Temporal trend in autism compared to temporal trend in cumulative amount of aluminum adjuvant administered post-natally to U.S. infants and toddlers by 2,6,12 and 18 months via immunization according to the CDC recommended schedule. The aluminum curves are lagged by milestone age rounded down to nearest year (i.e., 0,0,1, and 1 years, respectively).


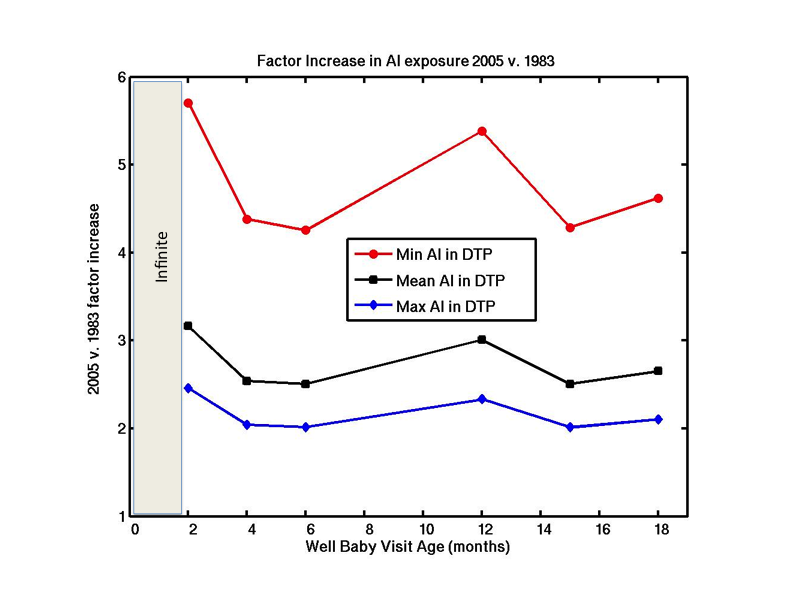


Figure S7b. Relative increase in aluminum adjuvant exposure in 2005 compared to 1983, plotted as a function of milestone age for U.S. infants and toddlers following the CDC recommended schedule. An infinite increase is shown for newborns and 1 month-olds because their exposure was 0 in 1983 prior to the addition of the HepB birth dose circa 1992. Since the relative increases at all other milestone ages depend strongly on the manufacturer and Al content assumed for DPT (170 or 600 mcg/dose) and DTaP (170, 230, 500, or 625 mcg/dose), three different curves that assume minimum, mean, and maximum Al content for DPT and DTaP are shown.


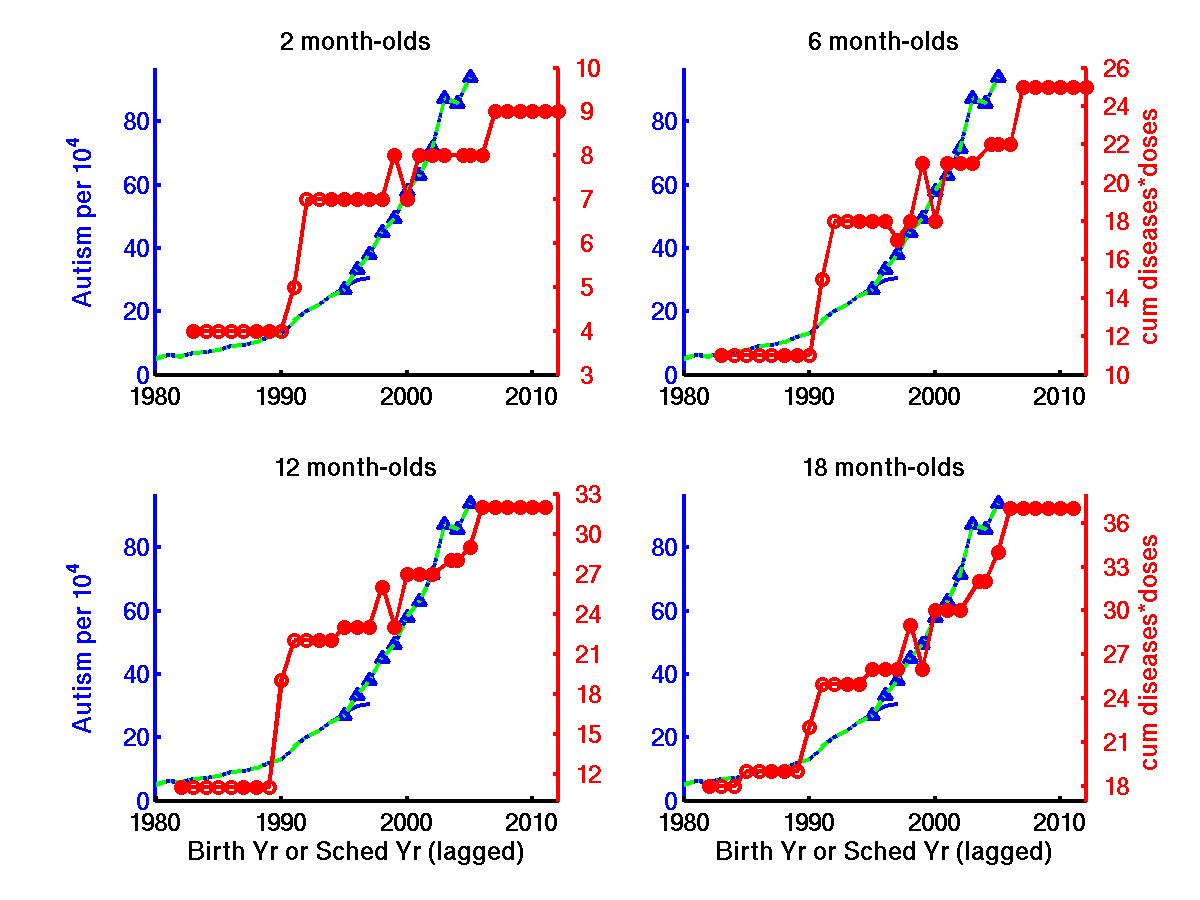


Figure S8. Temporal trend in autism compared to temporal trend in cumulative number of immunizations administered to U.S. infants and toddlers by by 2,6,12 and 18 months via immunization according to the CDC recommended schedule. The immunization curves are lagged by milestone age rounded down to nearest year (i.e., 0,0,1, and 1 years, respectively).


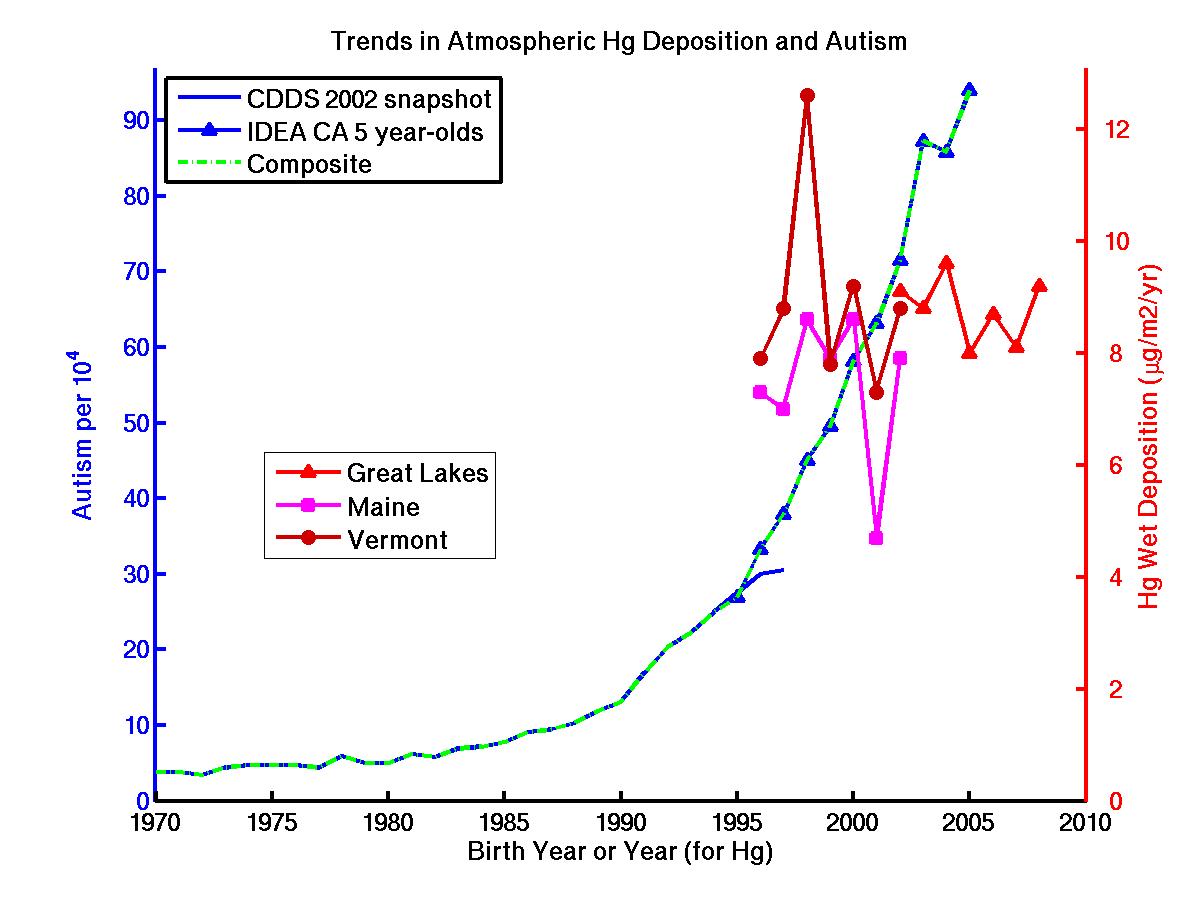


Figure S9a. Temporal trend in autism compared to temporal trend in wet deposition of atmospheric Hg observed over the Great Lakes and northeastern U.S (*Vanarsdale et al.*, 2005; *Risch et al.*, 2012).


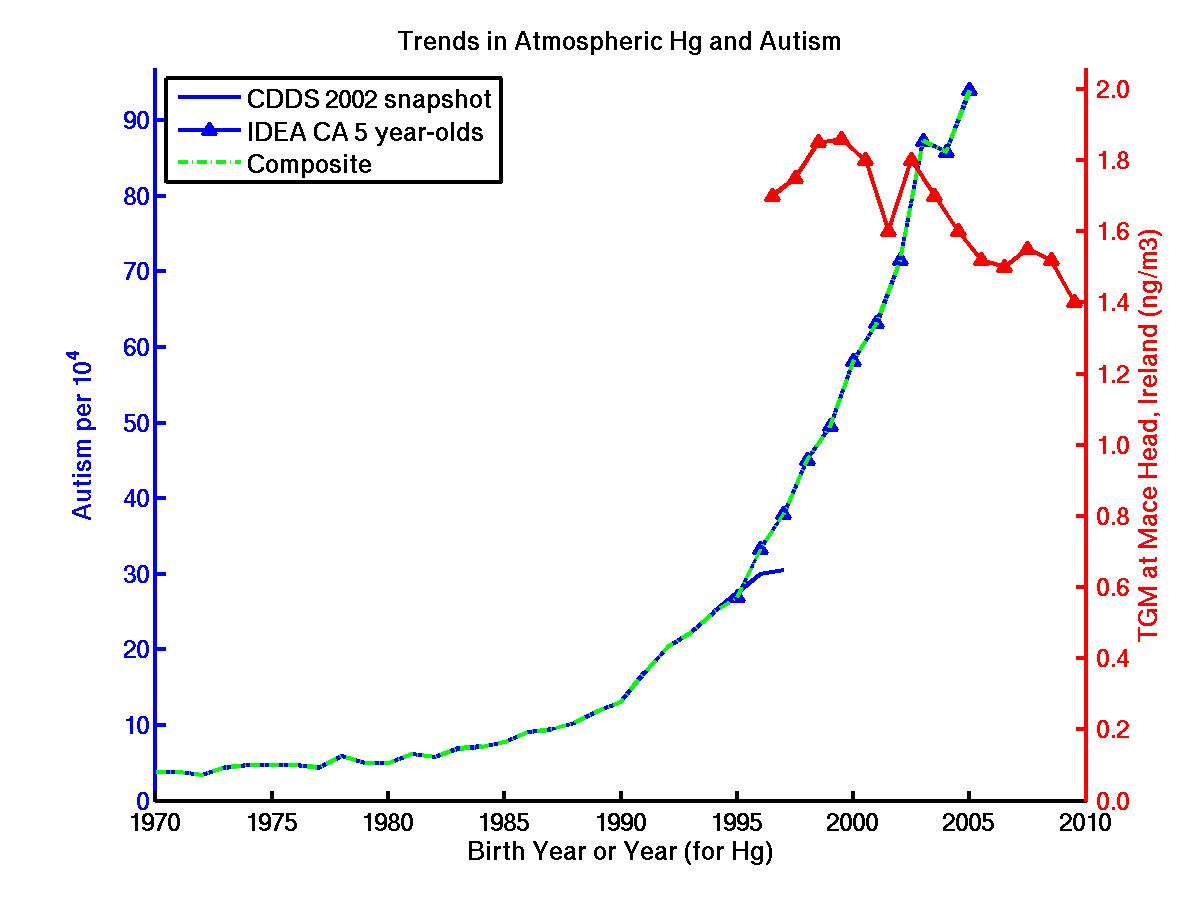


Figure S9b. Temporal trend in autism compared to temporal trend in total gaseous mercury (TGM) observed at Mace Head, Ireland (*Slemr et al.*, 2011).


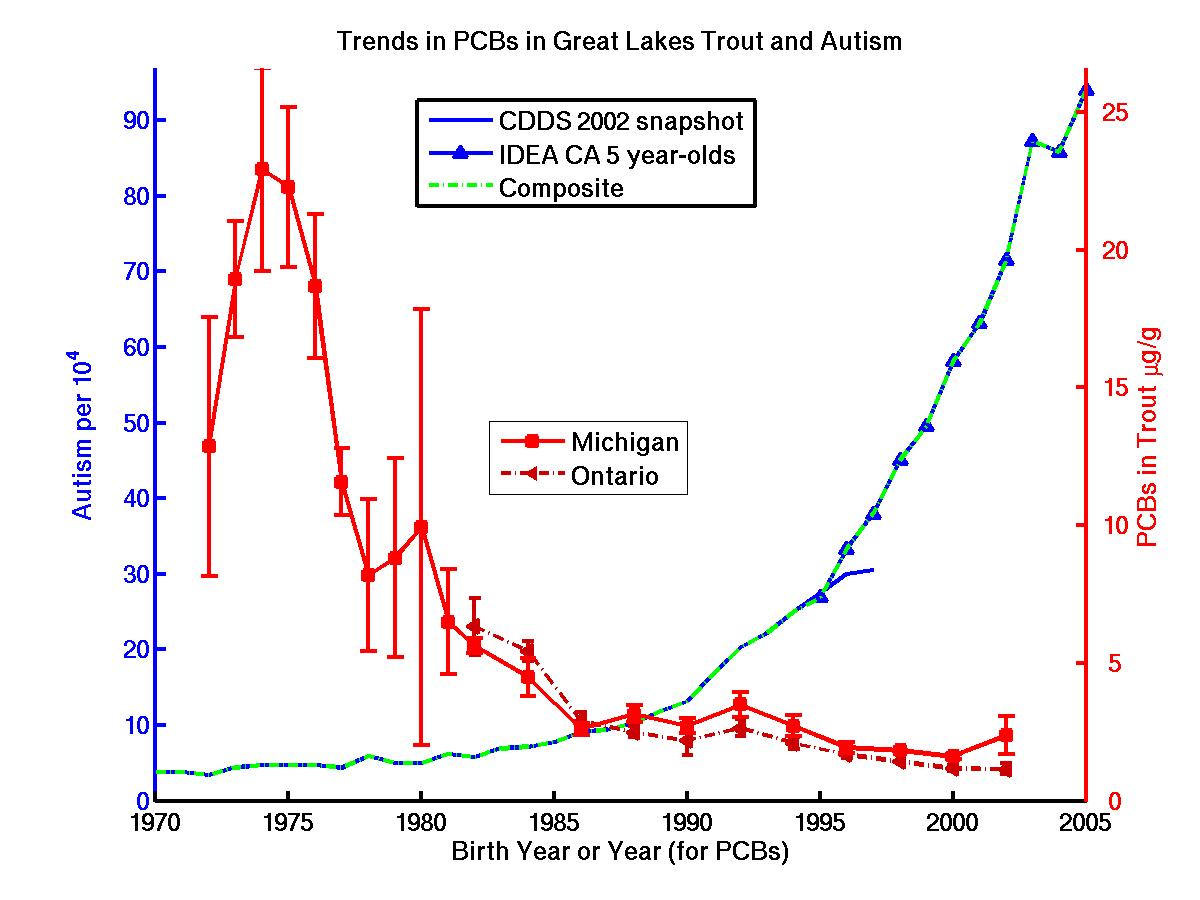


Figure S10. Temporal trend in autism compared to temporal trend in PCB concentrations in Great Lakes trout.


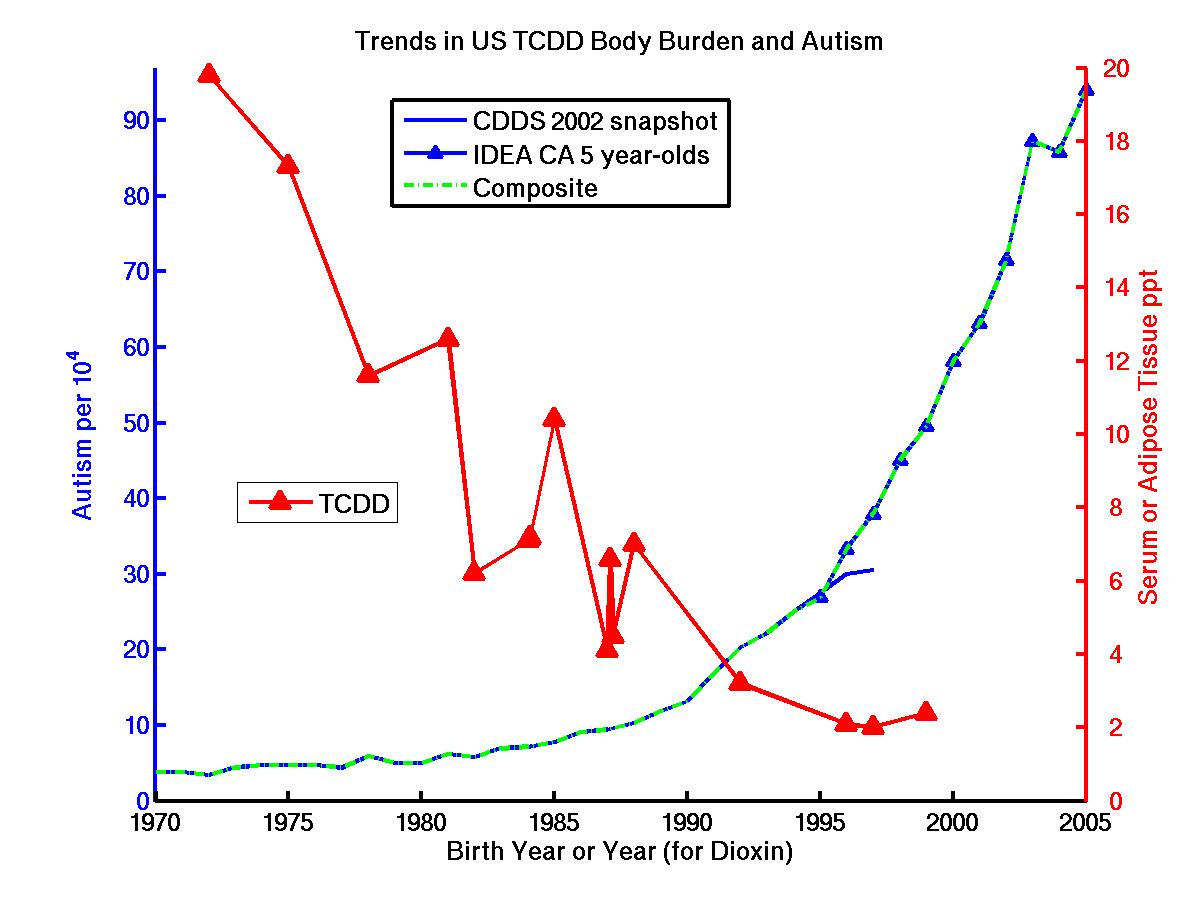


Figure S11. Temporal trend in autism compared to temporal trends in body burden of 2,3,7,8-tetrachlorodibenzo-p-dioxin (TCDD) in U.S. serum and adipose tissue (*Aylward et al.*, 2002).


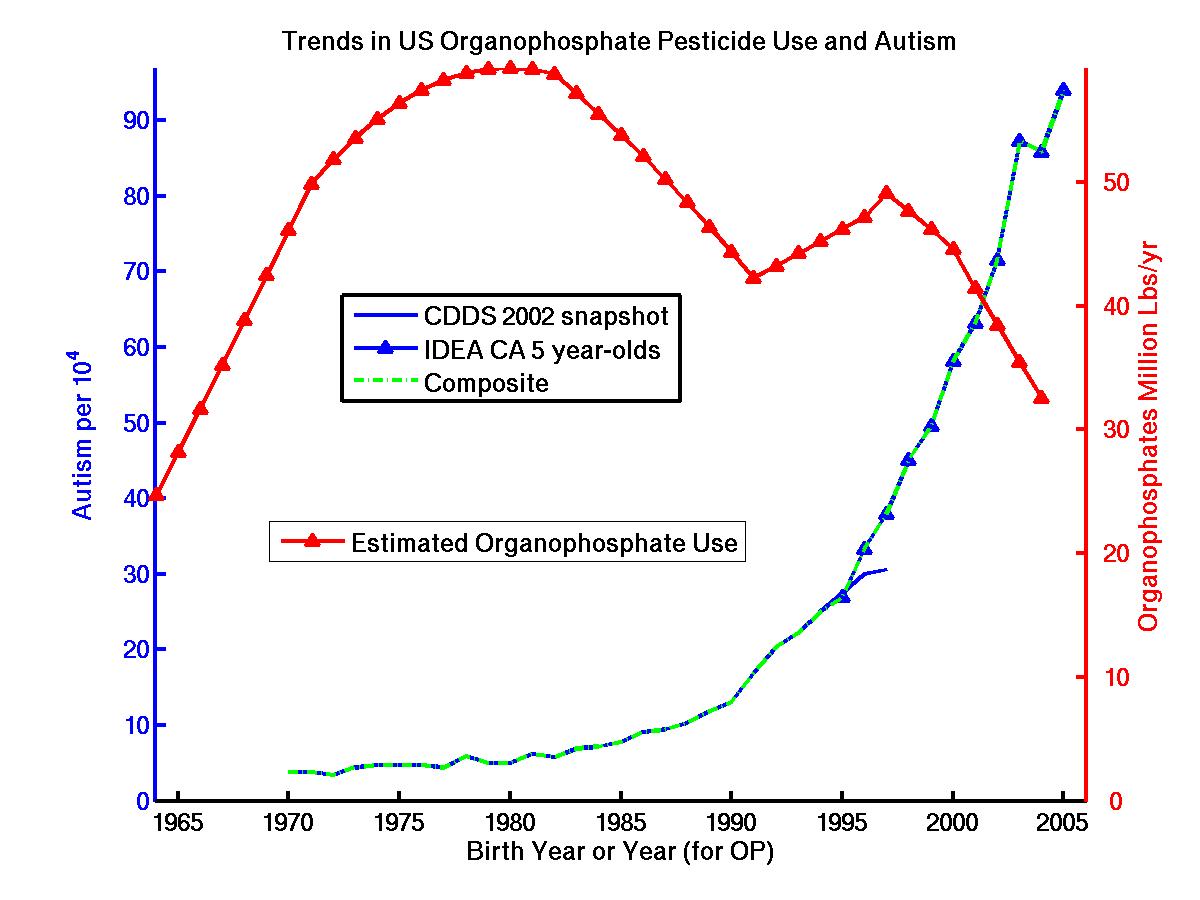


Figure S12a. Temporal trend in autism compared to temporal trend in U.S. organophosphate insecticide consumption (roughly estimated based on Tables 4.3.1 and 4.3.2 in *Osteen and Livingston*, 2006).


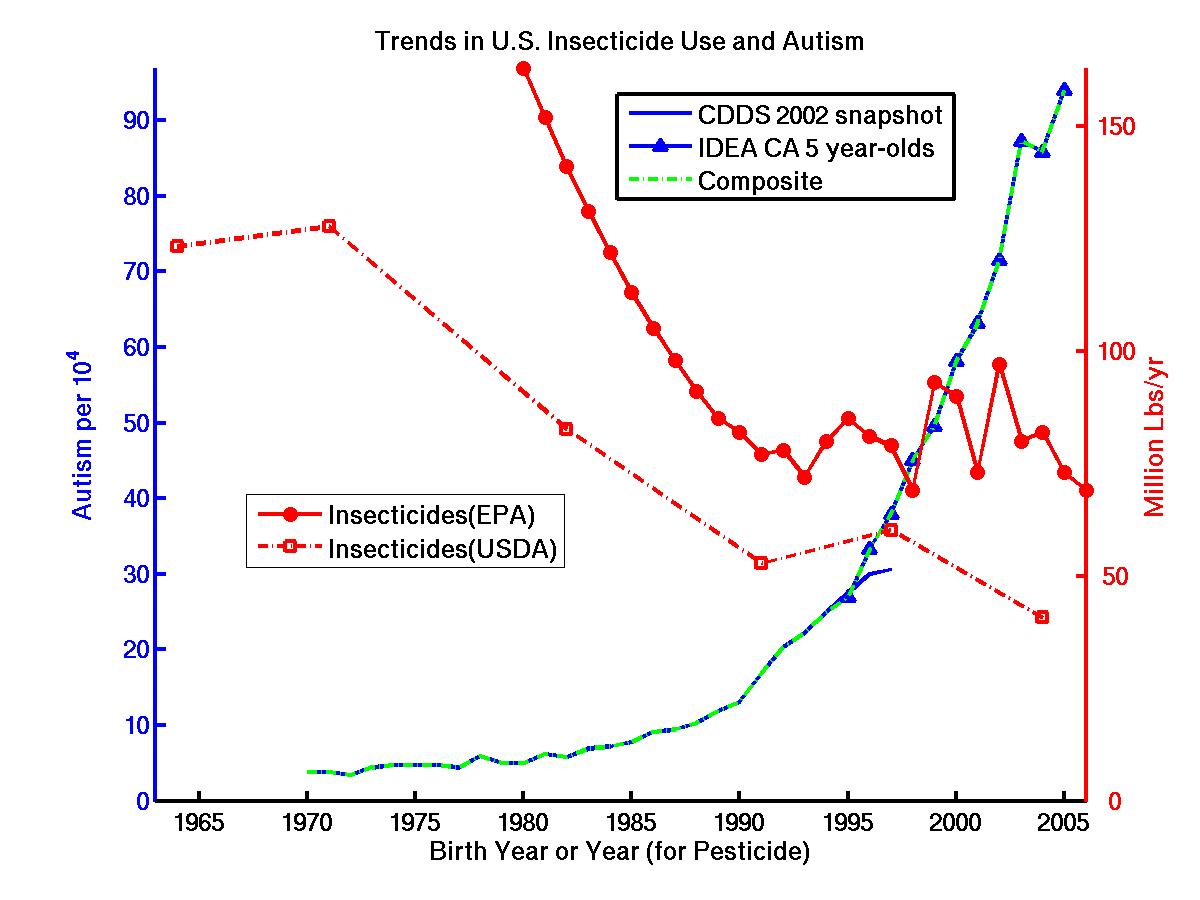


Figure S12b. Temporal trend in autism compared to temporal trend in U.S. agricultural insecticide consumption (*Osteen and Livingston*, 2006; *Grube et al.*, 2011).


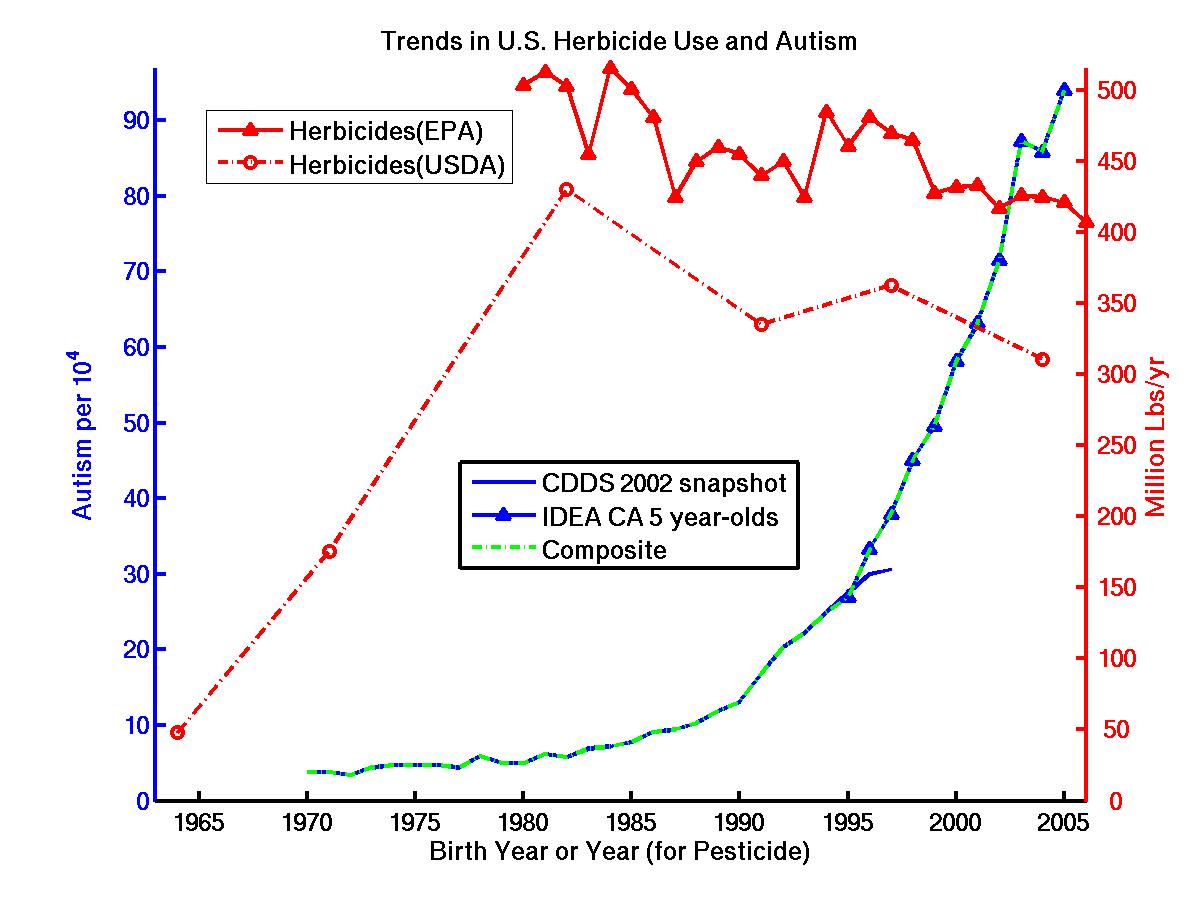


Figure S12c. Temporal trend in autism compared to temporal trend in U.S. agricultural herbicide consumption (*Osteen and Livingston*, 2006; *Grube et al.*, 2011).


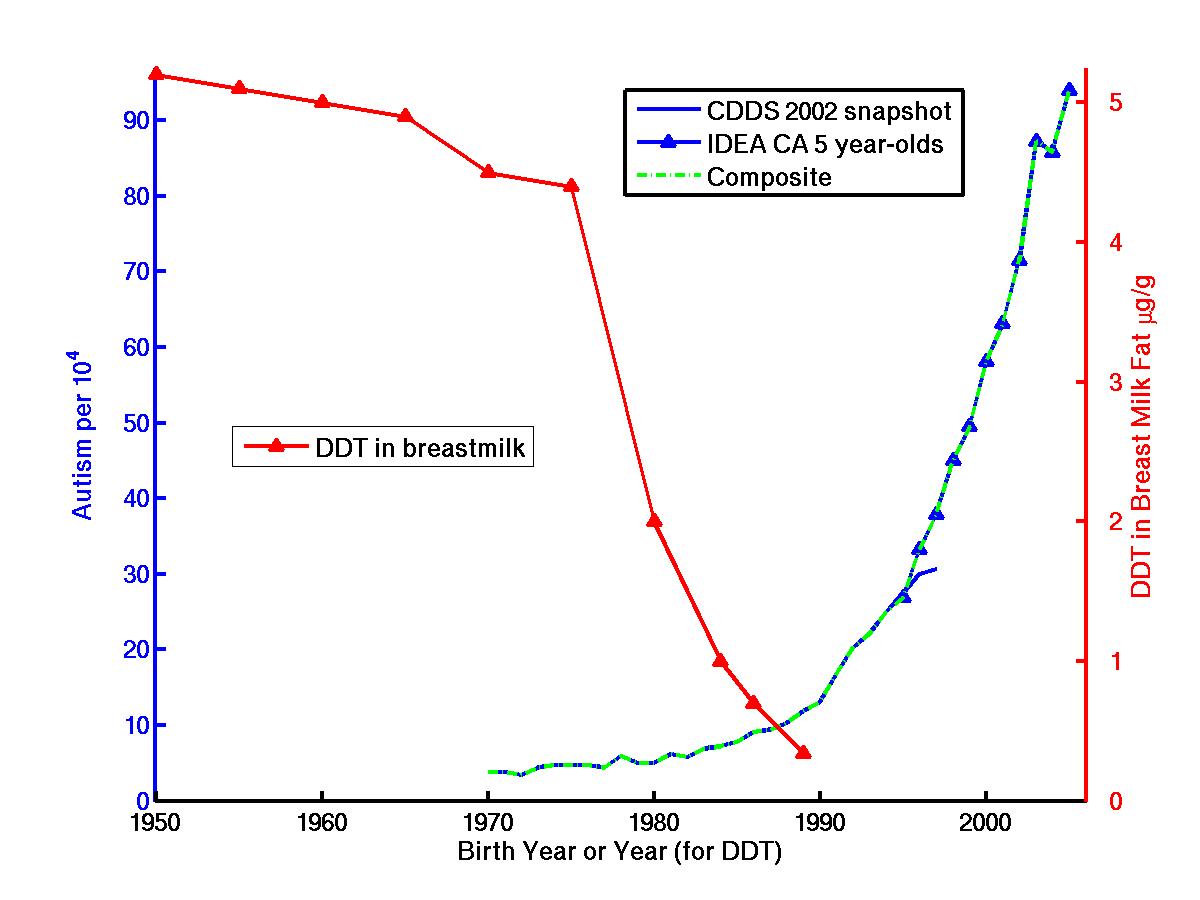


Figure S13. Temporal trend in autism compared to temporal trend in the organochlorine pesticide DDT measured in breastmilk fat from North American women. Data symbols are selected points from a spline fit to the breastmilk data (*Smith*, 1999). Note that DDT and many other organochlorines were banned by the US EPA in 1972.


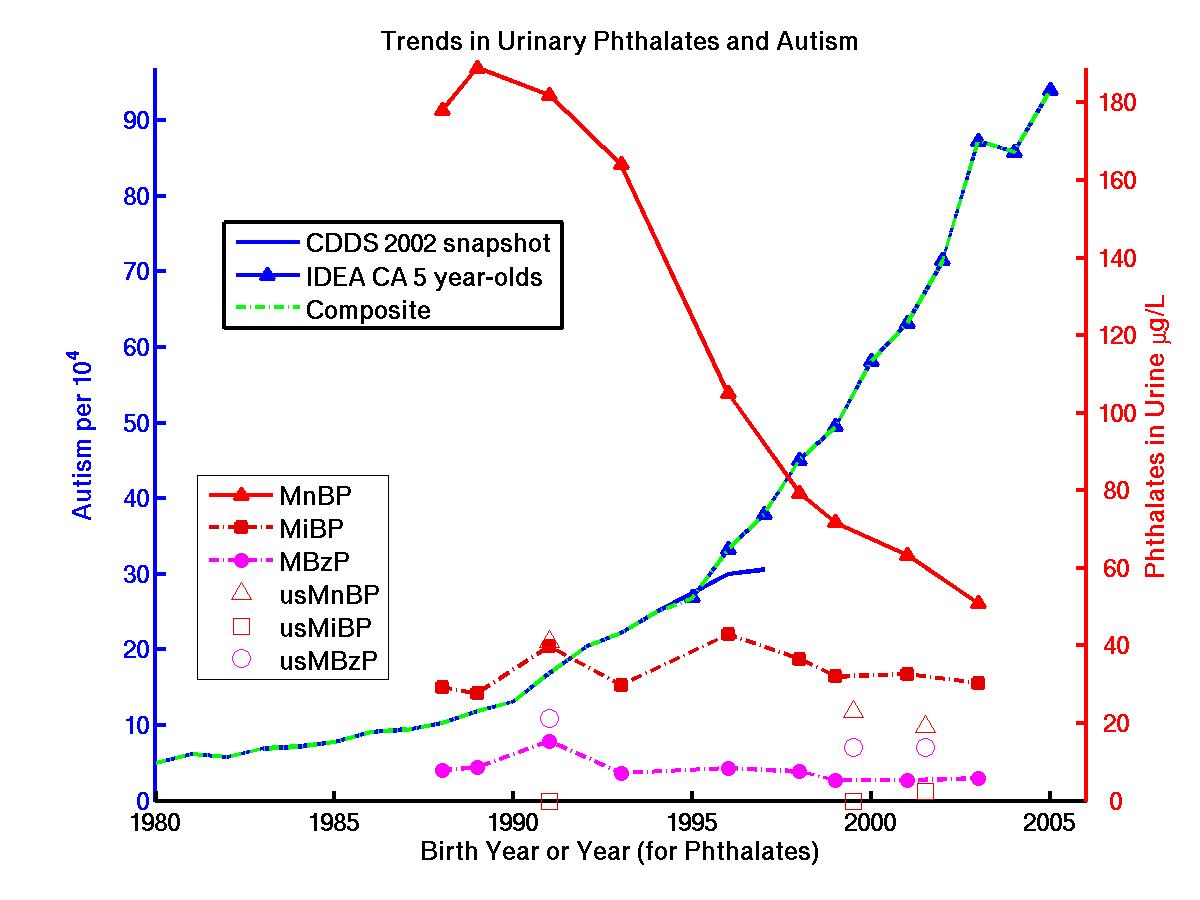


Figure S14a. Temporal trend in autism compared to temporal trends in 3 of the 10 most abundant phthalate metabolites measured in 24-hour urine samples of young adults living in Germany (*Wittassek et al.*, 2007). Available U.S. data compiled by Wittassek *et al.* are also shown as open symbols.


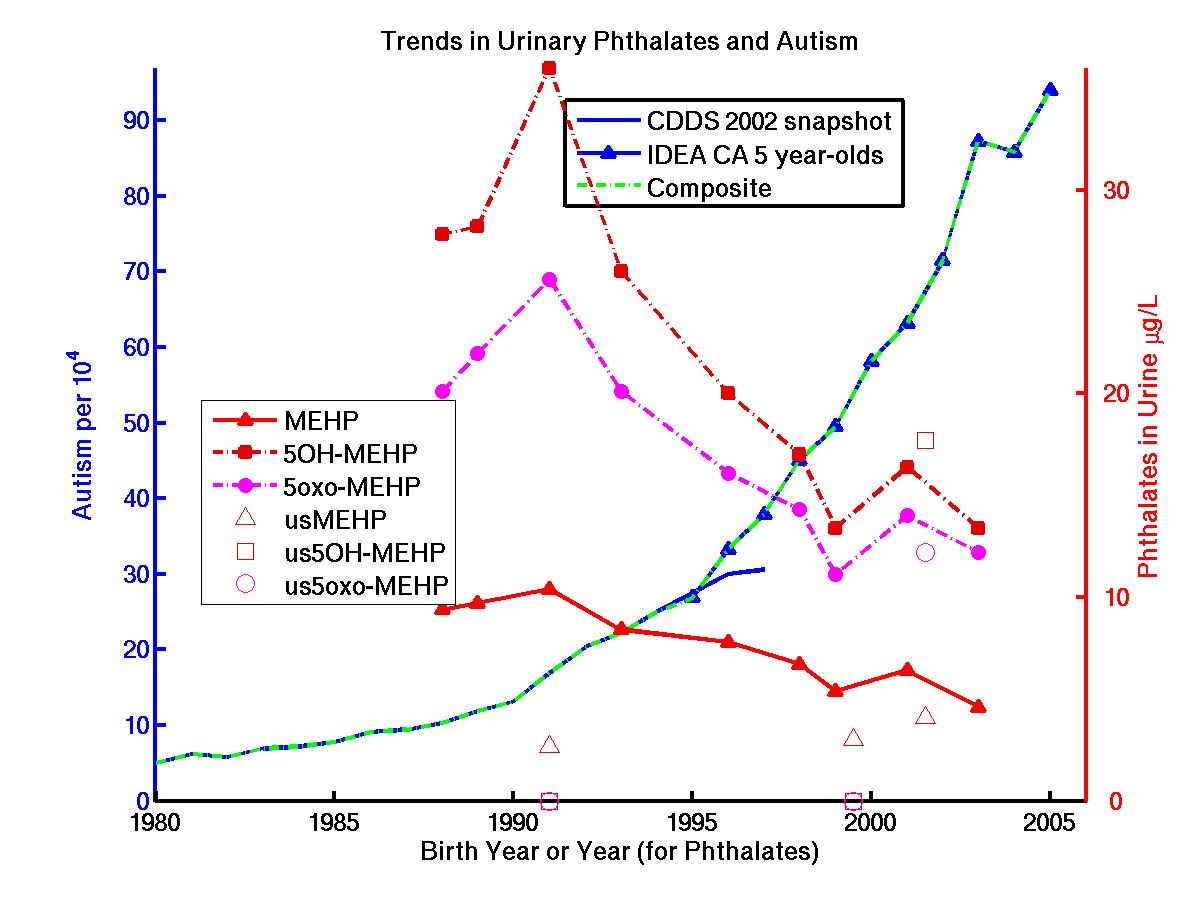


Figure S14b. Temporal trends in autism compared to temporal trends in 3 of the 5 most abundant metabolites of the phthalate DEHP measured in 24-hour urine samples of young adults living in Germany (*Wittassek et al.*, 2007). Available U.S. data compiled by Wittassek *et al.* are also shown as open symbols.


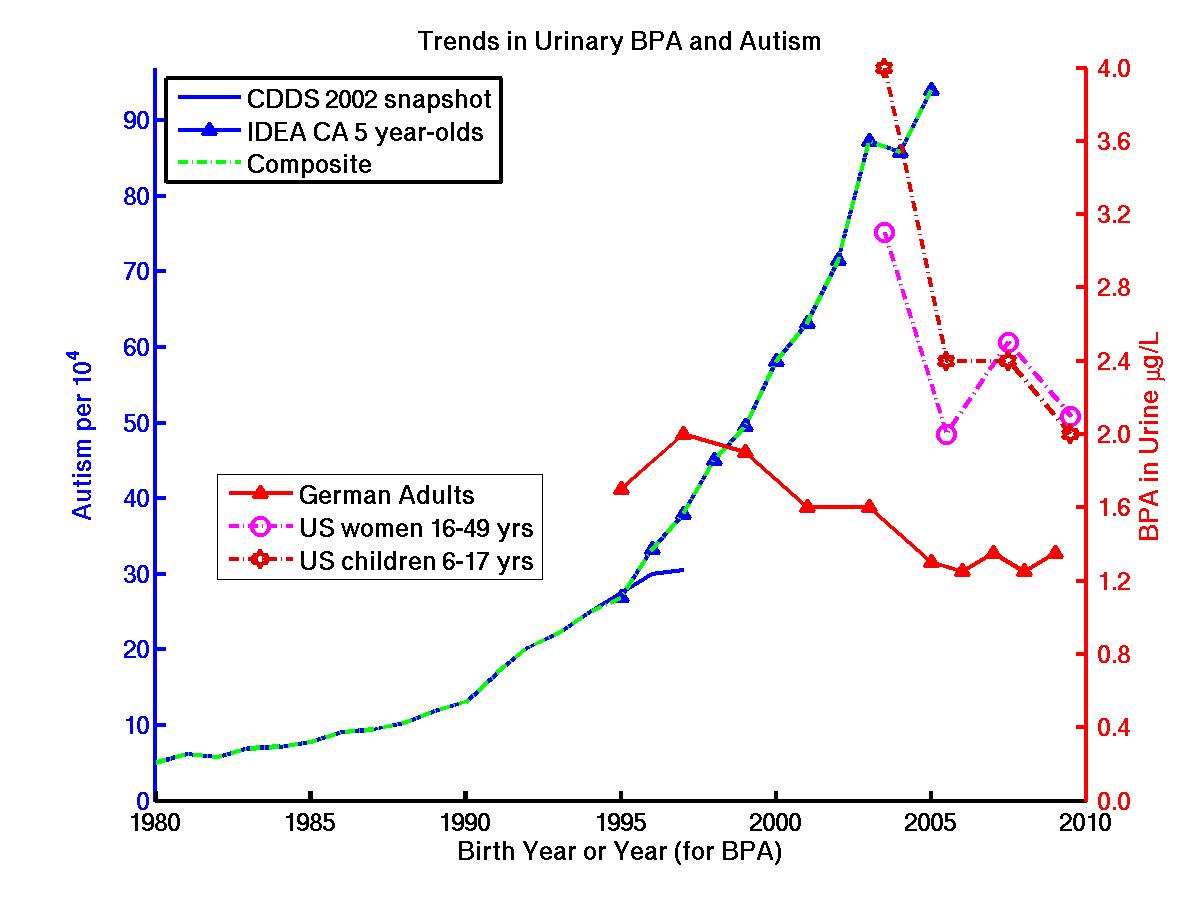


Figure S15. Temporal trend in autism compared to temporal trend in bisphenol A (BPA) measured in 24-hour urine samples of young adults living in Germany (*Kolossa-Gehring et al.*, 2012). Available U.S. data are also shown (*USEPA*, 2013).


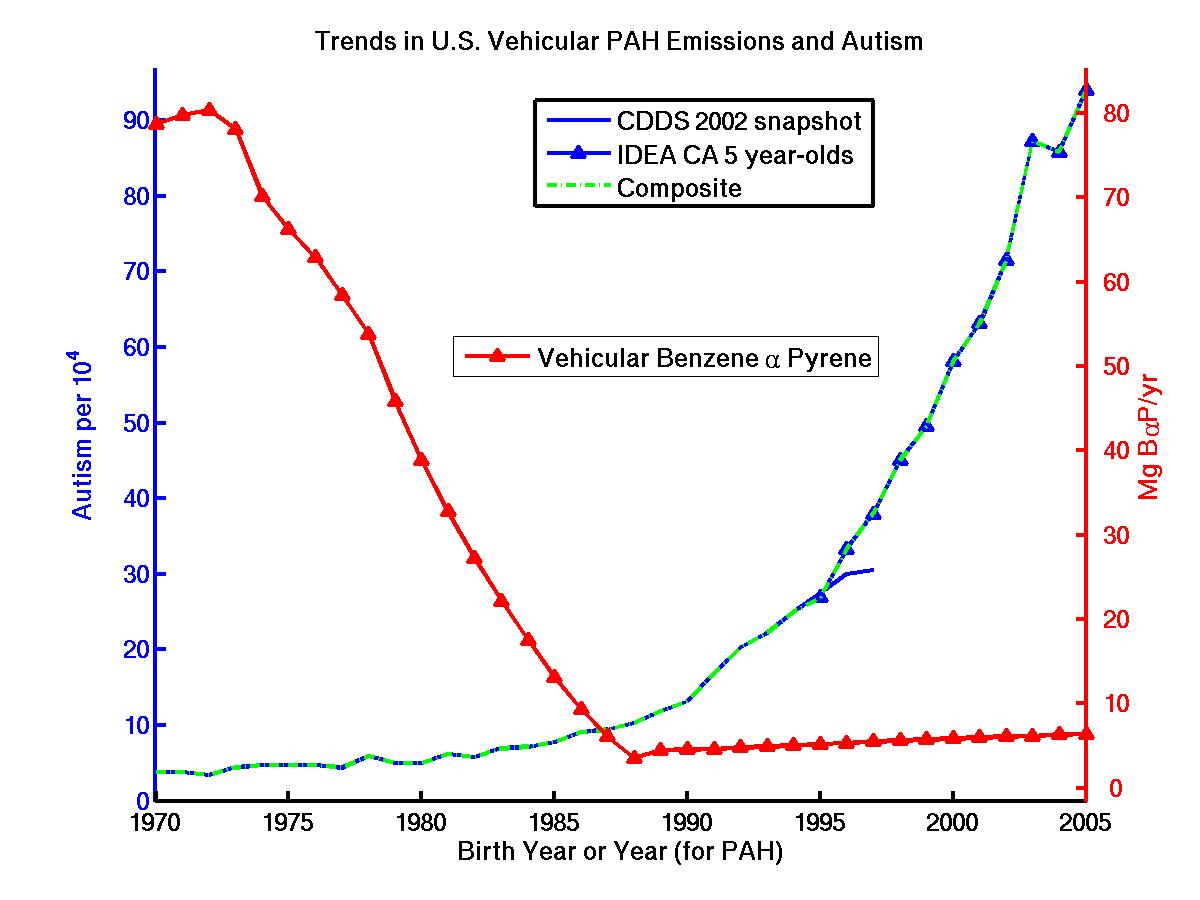


Figure S16a. Temporal trend in autism compared to temporal trend in total Benzene α Pyrene (BaP), a carcinogenic PAH, emitted by U.S. vehicular traffic. Estimated by multiplying changing U.S. fleet BaP emission factors (*Beyea et al.*, 2008) by total vehicle miles traveled from *Davis et al.* (2010).


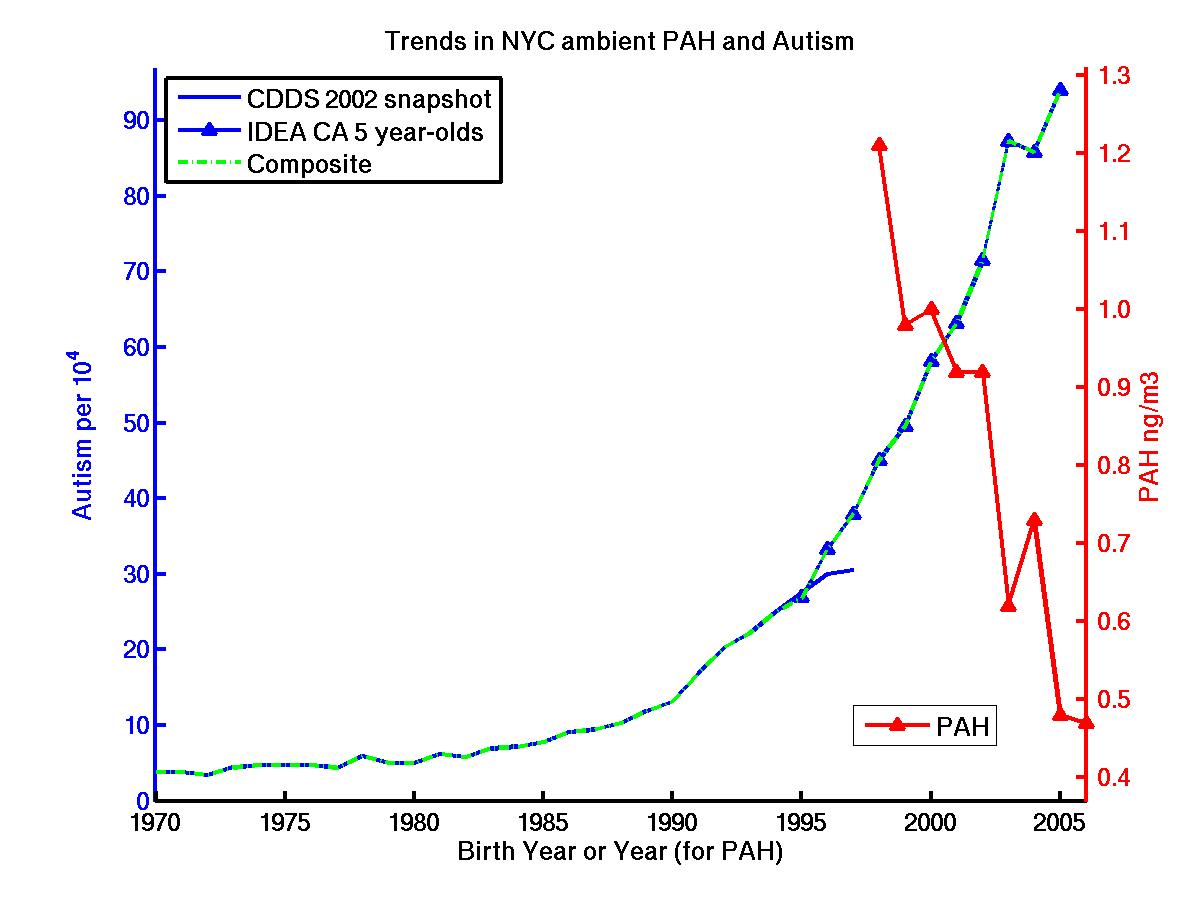


Figure S16b. Temporal trend in autism compared to temporal trend in the sum of 8 PAHs as measured using mobile personal air monitors in New York City (*Narvaez et al.*, 2008).


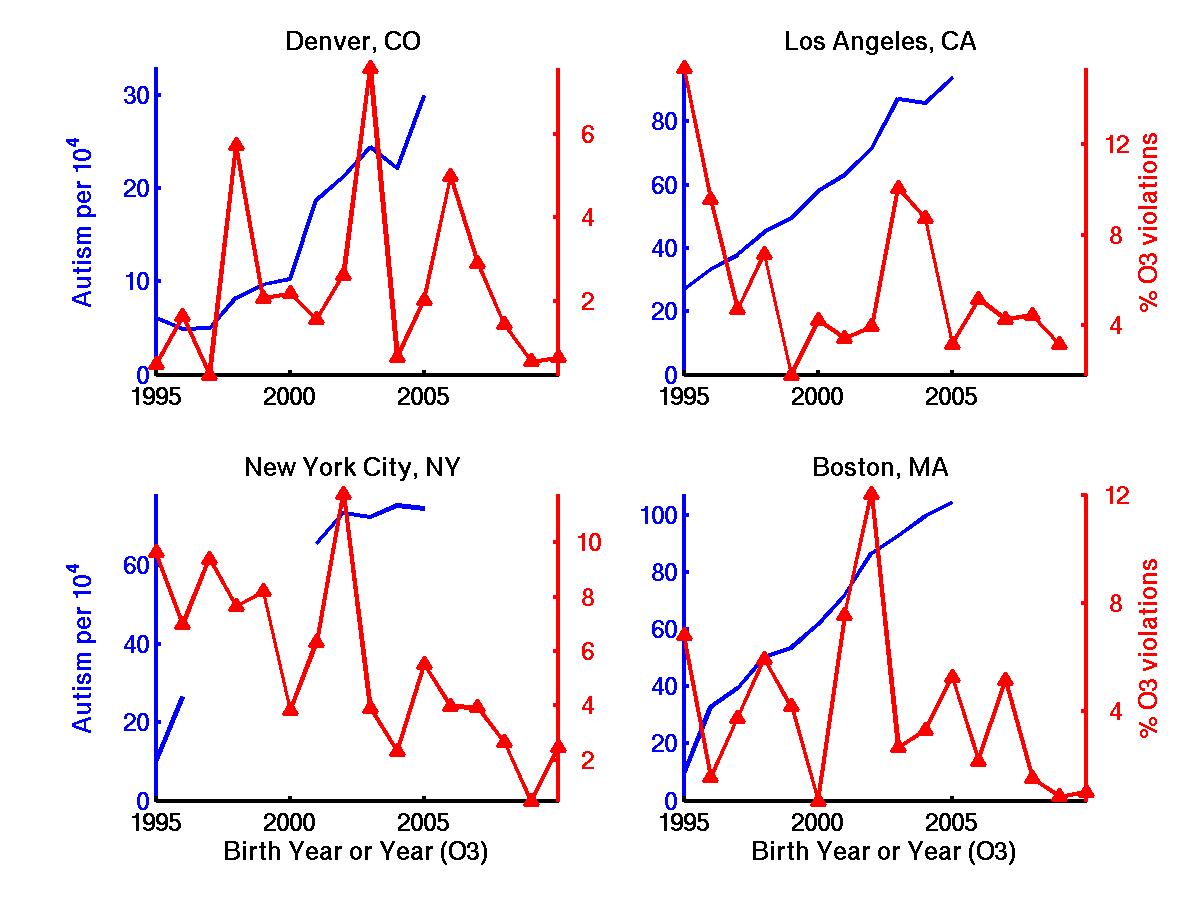


Figure S17. Temporal trends in autism compared to temporal trends in ground-level ozone violations (8-hour, 75 ppb standard) between April 1 and September 30. Autism data are state-level averages for IDEA 5 year-olds from the state in which the city is located. Ozone data are from U.S. EPA AQS (John Wong, personal communication).


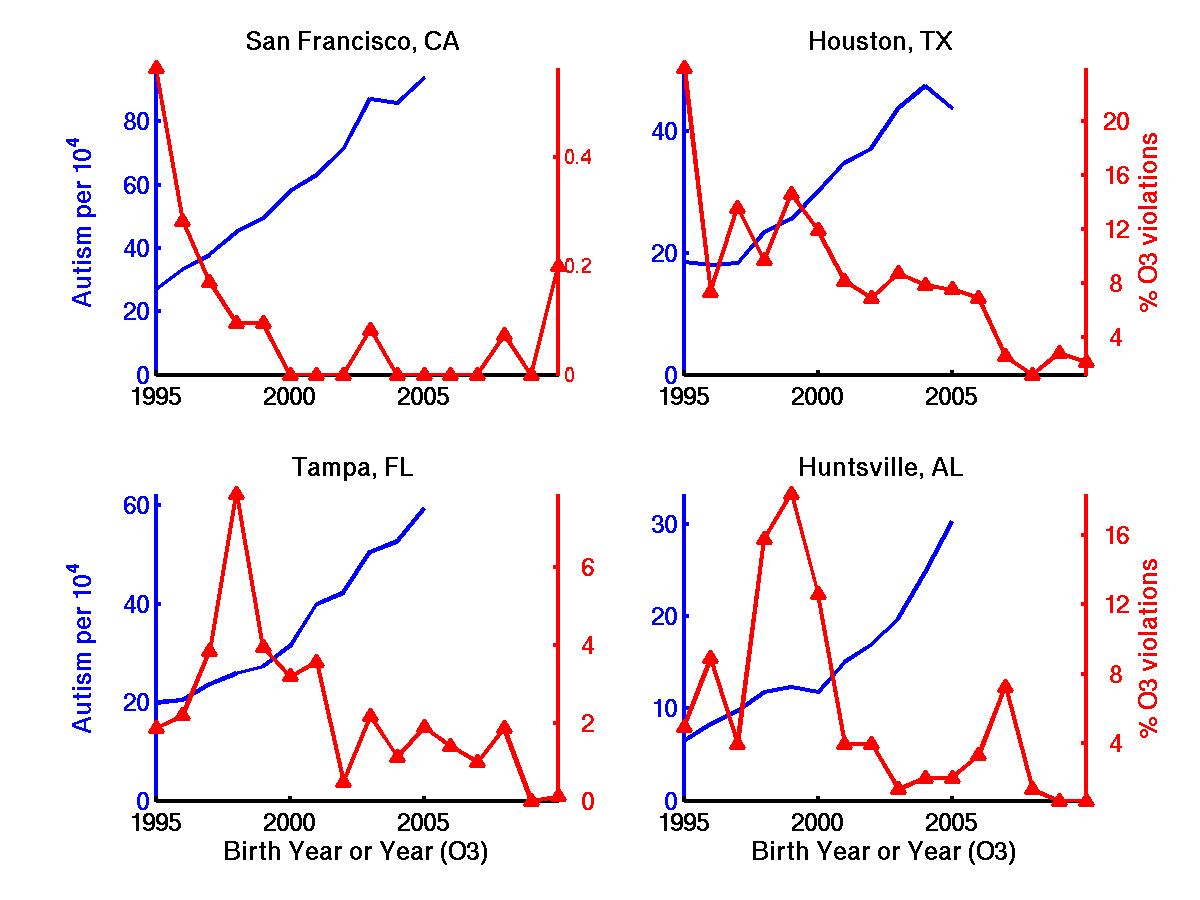


Figure S17 part 2.


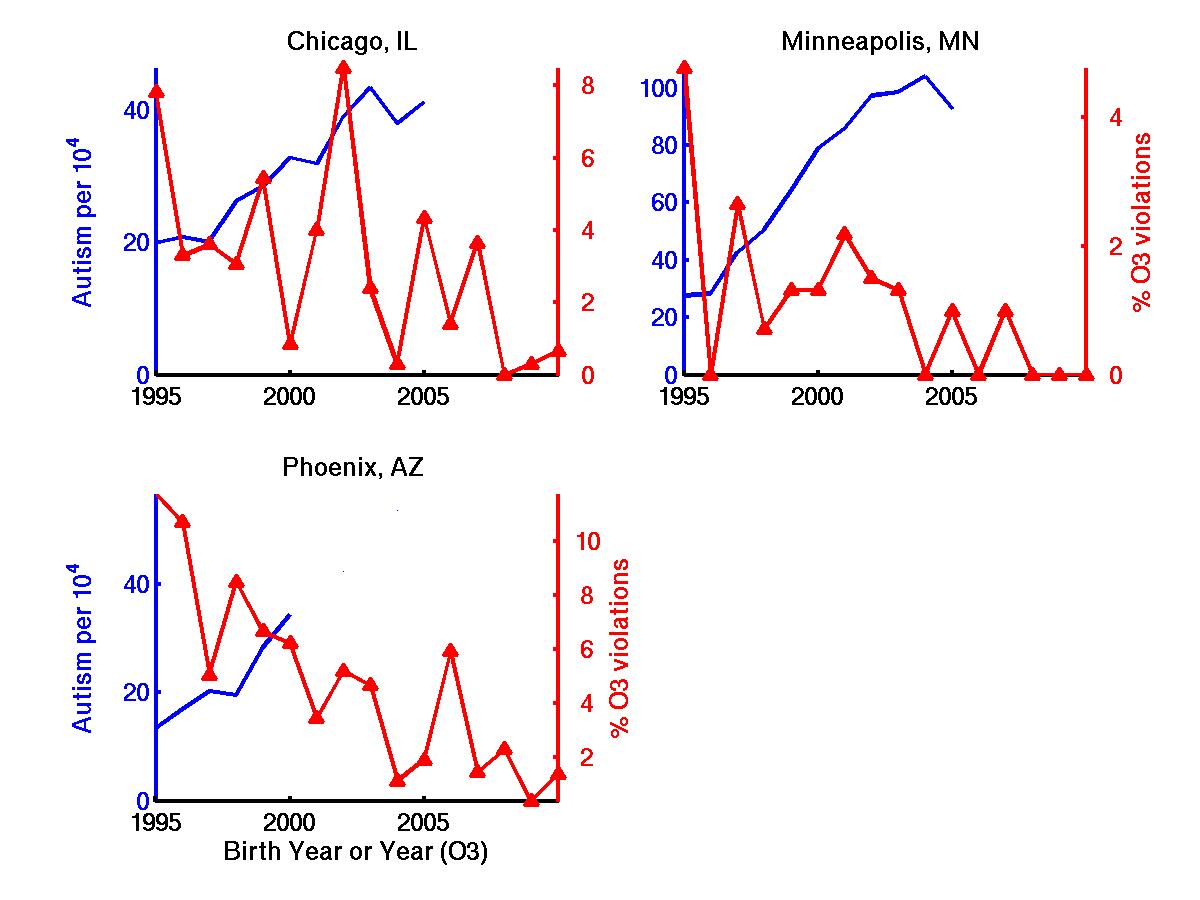


Figure S17 part 3.


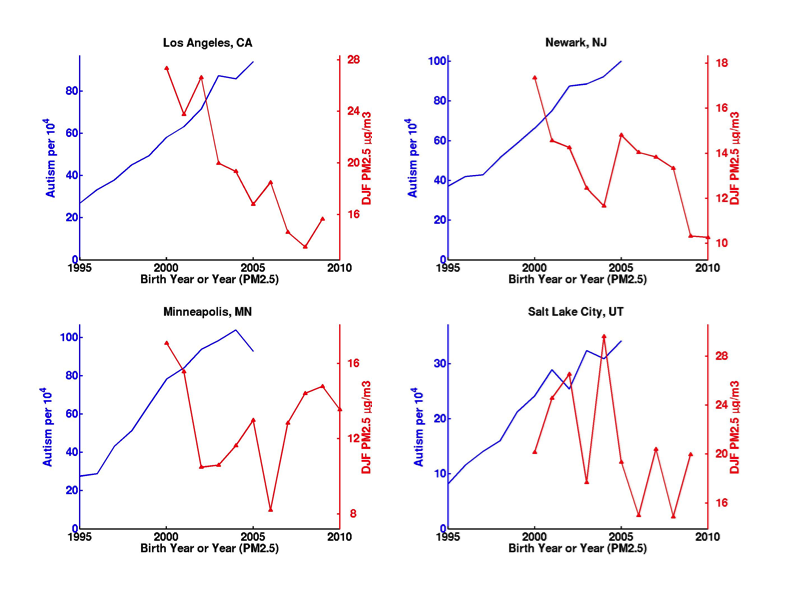


Figure S18. Temporal trends in autism compared to trends in mean winter (DJF) fine particulate matter < 2.5 microns (PM2.5) in 4 U.S. cities. Autism data are state-level averages for IDEA 5 year-olds from the state in which the city is located. PM2.5 data reflect the mean of 5 or more stations within 0.2 km of the city and are from U.S. EPA AQS (John Wong, personal communication).


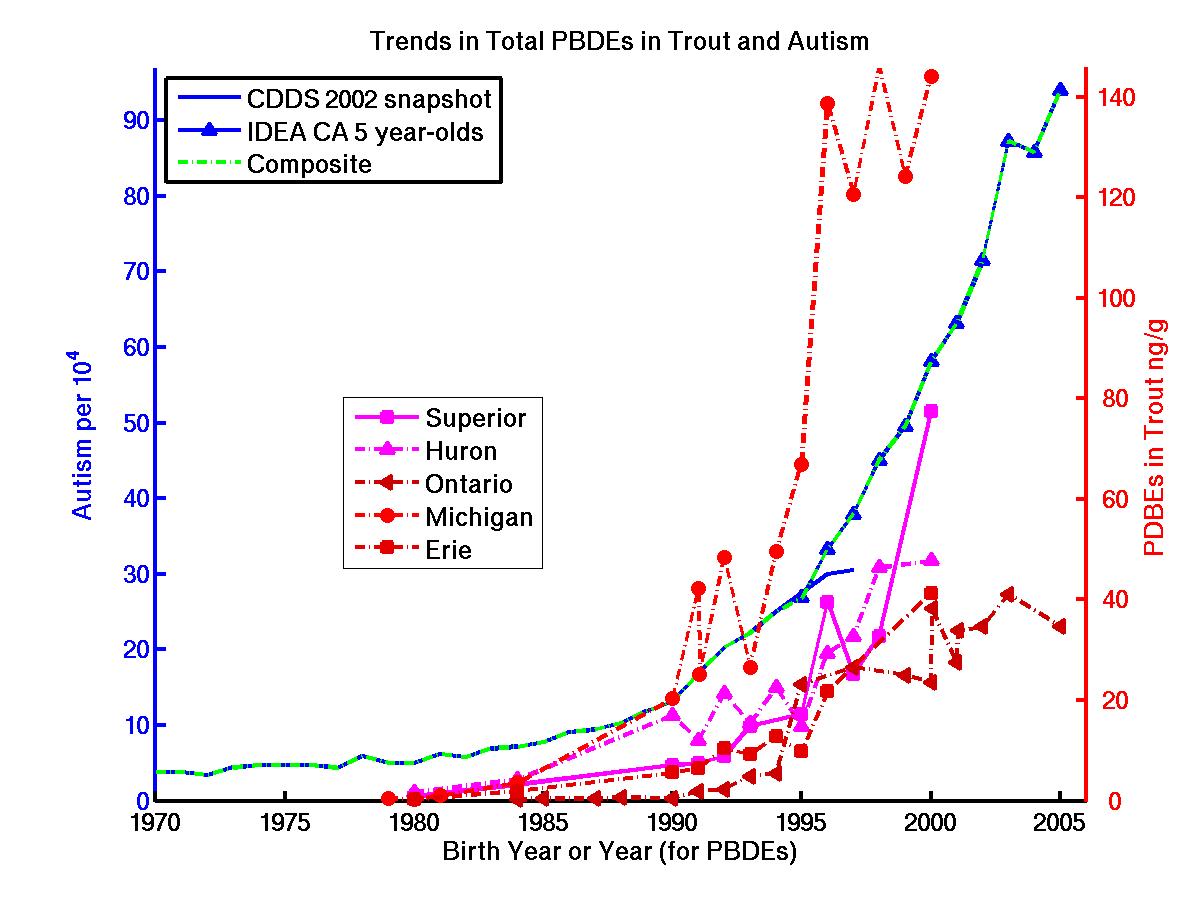


Figure S19. Temporal trend in autism compared to temporal trends in PBDE concentrations (sum of 47, 99, 100, and 153 congeners) in Great Lakes trout.


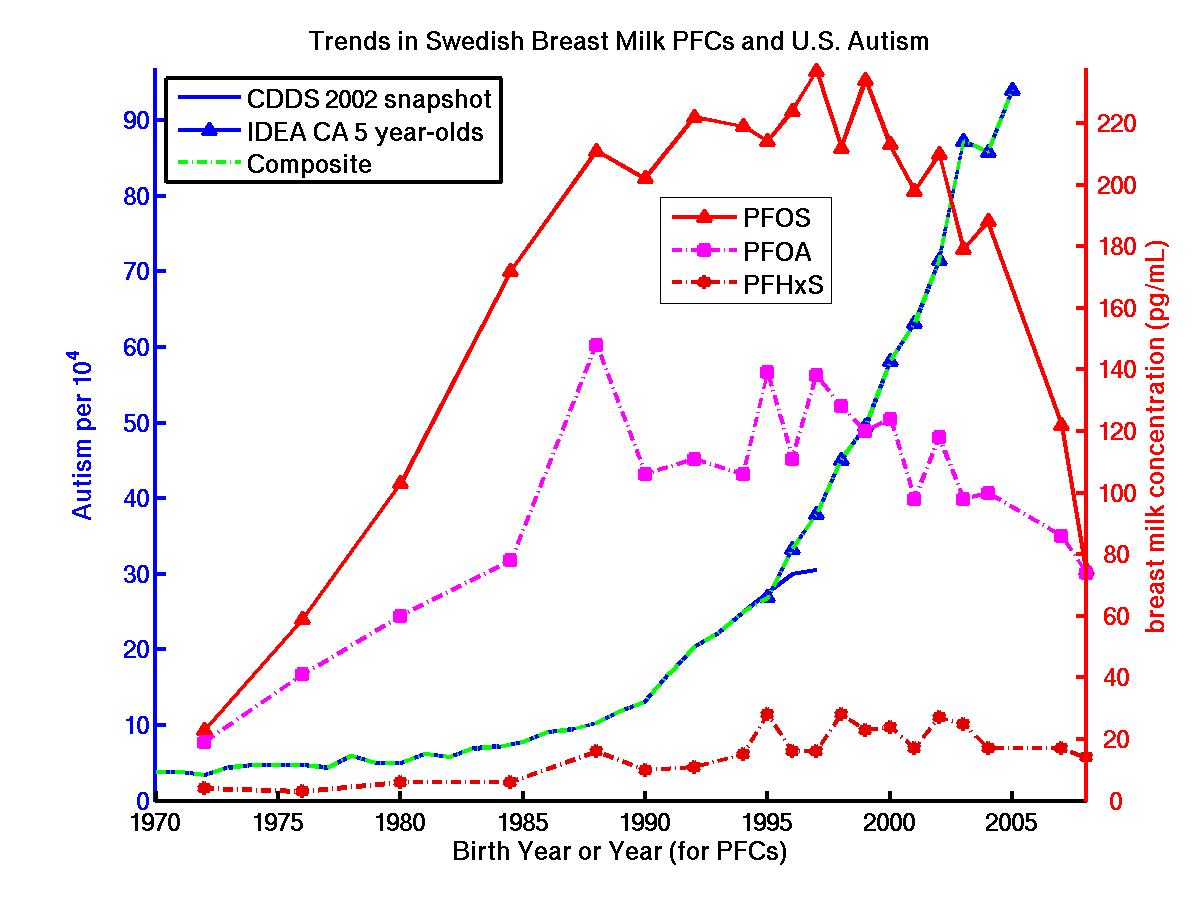


Figure S20a. Temporal trend in U.S. autism compared to temporal trends in the 3 most abundant pefluorinated compounds (PFCs) measured in Swedish breastmilk (*Sundstrom et al.*, 2011).


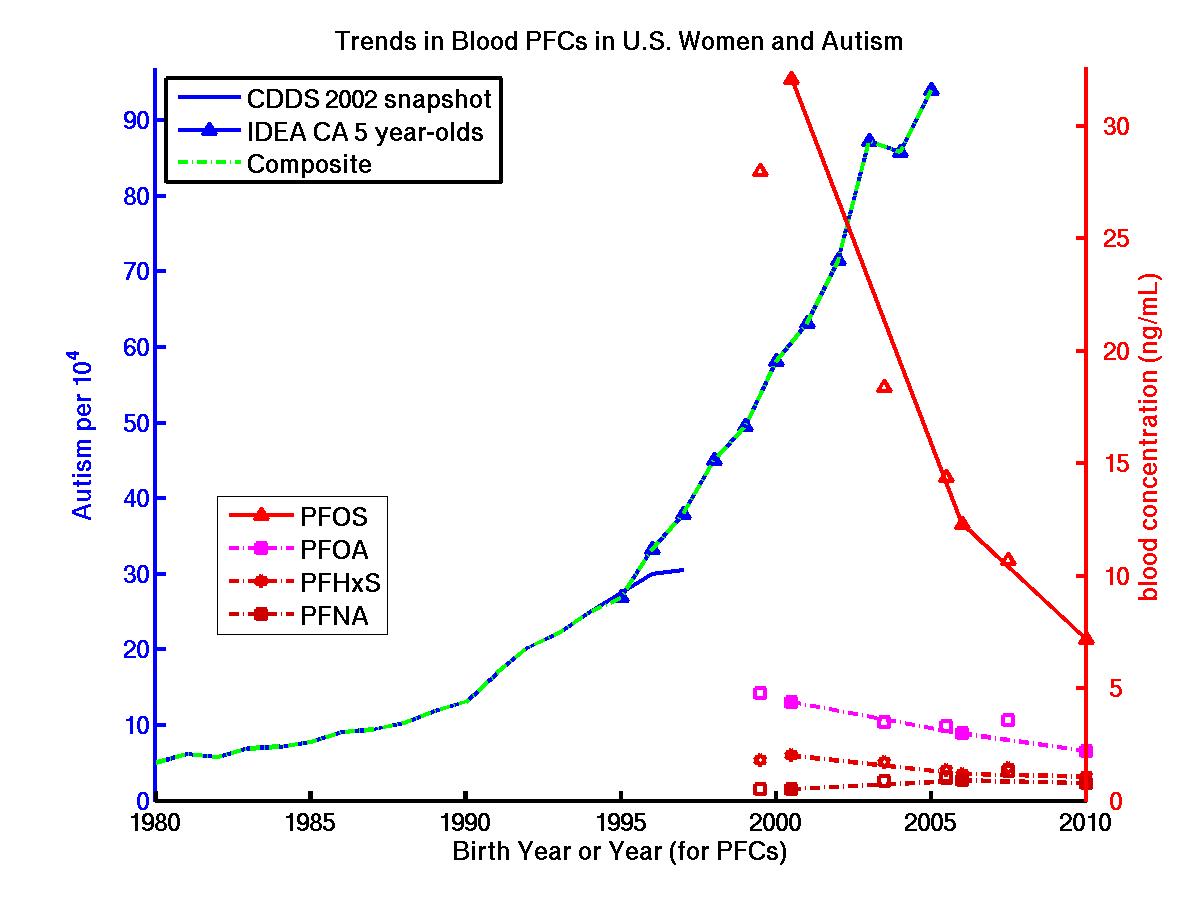


Figure S20b. Temporal trend in autism compared to temporal trends in the 4 most abundant pefluorinated compounds (PFCs) measured in U.S. women’s blood. Solid symbols are Red Cross blood donor data from *Olsen et al.* (2012). Open symbols are NHANES data from *Kato et al.* (2011).


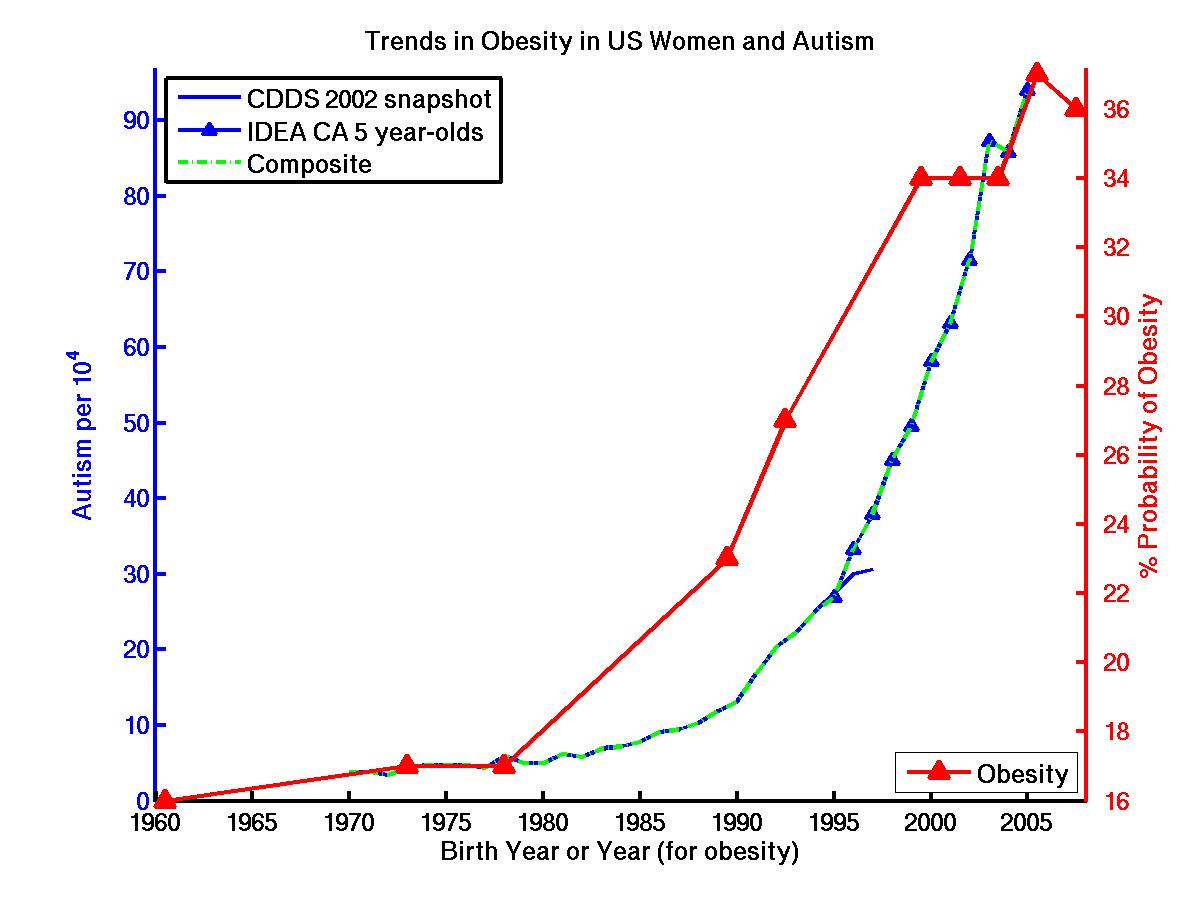


Figure S21. Temporal trend in autism compared to temporal trend in the % probability* of obesity among U.S. women, as calculated by *Ljungvall and Zimmerman* (2012) using ten NHANES surveys spanning from 1959-1962 to 2007-2008. Obesity year reflects midpoint of the NHANES survey. *The term “probability” reflects the fact that NHANES sample weights are applied to the raw survey data in a parametric equation (i.e., with coefficients and interaction terms applied to variables of race and ethnicity) in order to obtain accurate nationally representative estimates of sample statistics.

Table S1. Comparison of temporal trends in U.S. autism vs. suspected temporal drivers

| Suspected Agent | *R CDDS | **R Composite | Index | Period of Overlap | Beg/end  Ratio | Value at end of Period | Data Source |
| --- | --- | --- | --- | --- | --- | --- | --- |
| **Lead** | ***-0.81*** | ***-0.69*** | US children's blood Pb | 1970-2005 | 0.083 | 1.4 μg/dL | McCall and Land, 2004;  USEPA, 2013 |
| **Mercury** |  |  |  |  |  |  |  |
| U.S. Blood Levels | N/A | -0.53 | women age 30-39 | 1999-2005 | 0.95 | 1.02 μg/L | Caldwell et al., 2009 |
| U.S. Blood Levels | N/A | 0.07 | women age 20-29 | 1999-2005 | 0.95 | 0.77 μg/L | “ |
| U.S. Blood Levels | N/A | -0.25 | girls age 16-19 | 1999-2005 | 0.93 | 0.55 μg/L | “ |
| U.S. Blood Levels | N/A | -0.43 | children age 1-5 | 1999-2005 | 0.96 | 0.32 μg/L | “ |
| Seafood and Fish | **0.77** | **0.71** | US per capita consumed | 1970-2005 | 1.74 | 23.8 kg/yr | FAOSTAT |
| Hg in High Fructose Corn Syrup | **0.79** | **0.68** | US per capita consumed | 1970-2005 | 108 | 12 μg/day  range (0-25) | USDA, 2012 HFCS data and Dufault et al., 2009a Hg data |
| Atmospheric Mercury | N/A | -0.61 | TGM monitoring at Mace Head, Ireland | 1996-2005 | 0.9 | 1.6 ng/m^3^ | Slemr et al., 2011 |
| Atmos Hg Deposition | N/A | -0.41 | Great Lakes wet deposit. | 2002-2005 | 0.9 | 8 μg/m2/y | Risch et al., 2012 |
| *Vaccine Related* |  |  |  |  |  |  |  |
| Thimerosal | **0.85** | -0.23 | cumulative Hg by 18 months | 1983-2005 | 0.5 | 50 μg Hg | <http://www.cdc.gov/vaccines/>  schedules/past.html#prior-childhood |
| Total Doses | **0.89** | **0.89** | cumulative diseases x  doses by 18 months | 1983-2005 | 1.9 | 34 |  |
| Vaccine Aluminum | **0.87** | **0.83** | cumulative Al by 18 months | 1983-2005 | 2.4 | 3.65 mg Al | “ and Tomljenovic and Shaw, 2011 |
| **PCBs and Dioxins** |  |  |  |  |  |  |  |
| PCBs | ***-0.57*** | ***-0.51*** | trout in Lake Michigan | 1972-2002 | 0.18 | 2.4 μg/g | Carlson et al., 2012 |
| Dioxins | ***-0.75*** | ***-0.70*** | US serum, adipose tissue | 1972-1999 | 0.18 | 2.4 ppt | Aylward et al., 2002 |
| **Pesticides** |  |  |  |  |  |  |  |
| Organophosphates | -0.64 | -0.78 | US agricultural consumption (estimated) | 1970-2004 | 0.71 | 33x10^6^ lb/yr | Osteen and Livingston, 2006 |
| Total insecticides | ***-0.71*** | ***-0.50*** | US agri. consumption | 1980-2005 | 0.45 | 73x10^6^ lb/yr | Grube et al. 2011 |
| Total herbicides | -0.31 | ***-0.63*** | US agri. consumption | 1980-2005 | 0.84 | 421x10^6^ lb/y | “ |
| Suspected Agent | *R CDDS | **R Composite | Index | Period of Overlap | Beg/end  Ratio | Value at end of Period | Data Source |
| Glyphosate | **0.75** | **0.92** | US corn+soy application | 1990-2005 | 33 | 44 x10^6^ lb/y | USDA |
| Organochlorines | ***-0.83*** | N/A | DDT in North American breastmilk fat | 1970-1989 | 0.08 | 340 ng/g | Smith 1999 |
| **Endocrine Disruptors** | |  |  |  |  |  |  |
| BPA | 0.63 | -0.66 | German students’ urine | 1995-2005 | 0.76 | 1.3 μg/L | Kolossa-Gehring et al. 2012 |
| Metabolite (Phthalate) |  |  |  |  |  |  |  |
| MnBP(DnBP) | ***-0.83*** | ***-0.87*** | German students’ urine | 1988-2003 | 0.29 | 51 μg/L | Wittassek et al. 2007 |
| MiBP(DiBP) | 0.64 | -0.12 | German students’ urine | 1988-2003 | 1.04 | 30 μg/L | “ |
| MBzP(BBzP) | -0.33 | -0.63 | German students’ urine | 1988-2003 | 0.76 | 5.9 μg/L | “ |
| 50H-MEHP(DEHP) | ***-0.69*** | ***-0.81*** | German students’ urine | 1988-2003 | 0.48 | 13 μg/L | “ |
| **Automotive Exhaust** |  |  |  |  |  |  |  |
| Carbon monoxide | ***-0.93*** | ***-0.88*** | Highway emissions | 1970-2005 | 0.30 | 48 Mton/y | USEPA 2012 |
| NOx | ***-0.89*** | ***-0.88*** | Highway emissions | 1970-2005 | 0.51 | 6.4 Mton/y | “ |
| PM2.5 direct | ***-0.87*** | ***-0.89*** | Highway emissions | 1990-2005 | 0.42 | 0.14 Mton/y | “ |
| **Polycyclic Aromatic Hydrocarbons** | | |  |  |  |  |  |
| Benzene(alpha)Pyrene Vehicular Emissions | ***-0.67*** | ***-0.52*** | US fleet emission factors x vehicle miles traveled. | 1975-2005 | 0.08 | 6.4 Mg/yr | Beyea et al. 2008;  Davis et al. 2010 |
| Sum of 8 PAHs | N/A | -0.80 | air measured by mobile personal monitors | 1998-2005 | 0.4 | 0.48 ng/m^3^ | Narvaez et al. 2008 |
| **Polybrominated Biphenyl Ethers (PBDEs)** | | | |  |  |  |  |
| Total PBDEs | **0.80** | **0.89** | trout in Lake Ontario | 1984-2005 | 124 | 35 ng/g | Batterman et al. 2007 |
| Total PBDEs | **0.86** | **0.91** | trout in Lake Michigan | 1979-2000 | 343 | 144 ng/g | “ |
| **Perfluorinated Compounds (PFCs)** | | |  |  |  |  |  |
| PFOS | N/A | -0.81 | US women's blood | 1999-2005 | 0.48 | 14 ng/ml | Olsen et al. 2012; |
| PFOA | N/A | -0.81 | US women's blood | 1999-2005 | 0.72 | 3.3 ng/ml | “ and Kato et al. 2011 |
| PFHxS | N/A | -0.72 | US women's blood | 1999-2005 | 0.72 | 1.4 ng/ml | “ |
| PFNA | N/A | 0.81 | US women's blood | 1999-2005 | 1.9 | 1.0 ng/ml | “ |
| PFOS | **0.75** | **0.46** | Swedish breastmilk | 1972-2005 | 7.2 | 166 pg/ml | Sundstrom et al. 2011 |
| PFOA | **0.70** | 0.43 | Swedish breastmilk | 1972-2005 | 5.0 | 95 pg/ml | “ |
| PFHxS | **0.80** | **0.73** | Swedish breastmilk | 1972-2005 | 4.2 | 17 pg/ml | “ |

Bold = statistically significant positive correlation in temporal trends at 95% confidence level.

Bold/Italic = strong anticorrelation: statistically significant at 95% confidence level.

*Rcdds is the correlation coefficient between the temporal trend in the suspected toxin and the CDDS 2002 snapshot of autistic disorder prevalence for birth years 1970-1997.

**Rcomposite is the correlation coefficient between the temporal trend in the suspected toxin and autistic disorder prevalence for a composite of the CDDS 2002 snapshot data for birth years 1970-1994 and IDEA California 5 year-old data for birth years 1995-2005.
